# The role of GCNT1 mediated *O*-glycosylation in aggressive prostate cancer

**DOI:** 10.1038/s41598-023-43019-8

**Published:** 2023-10-09

**Authors:** Kirsty Hodgson, Margarita Orozco-Moreno, Emma Scott, Rebecca Garnham, Karen Livermore, Huw Thomas, Yuhan Zhou, Jiepei He, Abel Bermudez, Fernando Jose Garcia Marques, Kayla Bastian, Gerald Hysenaj, Emily Archer Goode, Rakesh Heer, Sharon Pitteri, Ning Wang, David J. Elliott, Jennifer Munkley

**Affiliations:** 1 Newcastle University Centre for Cancer, Newcastle University Institute of Biosciences, Newcastle, NE1 3BZ UK; 2 Newcastle University Centre for Cancer, Translational and Clinical Research Institute, Newcastle University, Paul O’Gorman Building, Newcastle upon Tyne, NE2 4HH UK; 3 Department of Oncology and Metabolism, The Mellanby Centre for Musculoskeletal Research, The University of Sheffield, Sheffield, UK; 4 Department of Radiology, Canary Center at Stanford for Cancer Early Detection, Stanford University, Palo Alto, CA 94304 USA; 5 Department of Urology, Freeman Hospital, The Newcastle Upon Tyne Hospitals NHS Foundation Trust, Newcastle upon Tyne, NE7 7DN UK

**Keywords:** Prostate cancer, Cancer models

## Abstract

Prostate cancer is the most common cancer in men and a major cause of cancer related deaths worldwide. Nearly all affected men develop resistance to current therapies and there is an urgent need to develop new treatments for advanced disease. Aberrant glycosylation is a common feature of cancer cells implicated in all of the hallmarks of cancer. A major driver of aberrant glycosylation in cancer is the altered expression of glycosylation enzymes. Here, we show that GCNT1, an enzyme that plays an essential role in the formation of core 2 branched *O*-glycans and is crucial to the final definition of *O*-glycan structure, is upregulated in aggressive prostate cancer. Using in vitro and in vivo models, we show GCNT1 promotes the growth of prostate tumours and can modify the glycome of prostate cancer cells, including upregulation of core 2 *O*-glycans and modifying the *O*-glycosylation of secreted glycoproteins. Furthermore, using RNA sequencing, we find upregulation of GCNT1 in prostate cancer cells can alter oncogenic gene expression pathways important in tumour growth and metastasis. Our study highlights the important role of aberrant *O*-glycosylation in prostate cancer progression and provides novel insights regarding the mechanisms involved.

## Introduction

Prostate cancer is a major cause of male cancer-related deaths around the world^1^. The progression of prostate cancer is driven by the androgen receptor (AR) and first line treatments for advanced prostate cancer include AR-targeted therapies^2^. However, in a significant number of cases, tumours progress to an aggressive state for which AR-targeted therapies become ineffective, known as metastatic castrate resistant prostate cancer (mCRPC)^3–5^. Several second-generation agents targeting AR signalling, including enzalutamide^6^, abiraterone^7^, and darolutamide^8^, can be used to treat mCRPC, but nearly all affected men will also develop resistance to these agents^3–5^ and there is an urgent need to discover new ways to treat prostate cancer.

Glycosylation is the most common, complex, and dynamic post-translational modification of lipids and proteins and is essential for every biological process^9^. Aberrant glycosylation is an established hallmark of cancer cells^10,11^ and glycans hold huge potential for cancer research. In prostate cancer, glycan alterations are common and typically include increased tumour sialylation, truncated *O*-glycans, increased core fucosylation, and changes to PSA glycosylation^12,13^. A key driver of aberrant glycosylation in cancer is the dysregulated expression of glycosyltransferase enzymes^14^. We previously revealed glycosylation is an androgen regulated process in prostate cancer cells and identified a set of glycosylation enzymes that are upregulated in clinical prostate tumour tissue^15,16^. In this study, we focus on GCNT1 (also known as C2GNT1), a glycosyltransferase enzyme that plays an essential role in the formation of core 2 branched *O*-glycans, an early step in *O*-glycan synthesis that promotes chain branching and elongation and is crucial to the final definition of *O*-glycan structure^17–19^.

Aberrant expression of GCNT1 has been related to aggressive disease in different types of cancers, including breast cancer^20^, prostate cancer^12,15,16,21–23^, chronic lymphocytic leukaemia (CLL)^24^, endometrial cancer^25^, colon cancer^26^, testicular germ cell cancer^27^ and bladder cancer^28^. In prostate cancer, GCNT1 is upregulated at both the gene and protein level^12,16,21–23^, and has been linked to recurrence after surgery^23^ and the spread of cancer cells outside of the prostate gland^21^. Findings show that upregulation of GCNT1 enhances the growth of orthotopic prostate tumours^29^, correlates with higher levels of core 2 *O*-SLe^X^ on PSA, PAP, and MUC1 proteins^22^, and can promote resistance to NK cell immunity^30^. However, the impact of GCNT1 on the global glycosylation of prostate cancer cells and the molecular mechanisms underlying the role GCNT1 in prostate cancer are largely unknown.

Here, we analyse four independent cohorts of patient RNA samples and further confirm upregulation of *GCNT1* gene expression in aggressive prostate cancer. Our findings reveal GCNT1 can alter the glycome of prostate cancer cells, specifically by upregulating the expression of core 2 *O*-glycan structures (including the cancer-associated glycan SLe^X^) and modifying the *O*-glycosylation of secreted glycoproteins. Using both in vitro and in vivo studies, we show that GCNT1 promotes the growth of prostate tumours and regulates oncogenic gene expression pathways that are important for disease progression. Our study highlights the role of GCNT1 in aggressive prostate cancer and provides novel insights into the role of aberrant glycosylation in disease pathology.

## Results

### *GCNT1* gene expression levels are upregulated in aggressive prostate cancer tissue

**Figure 1:**
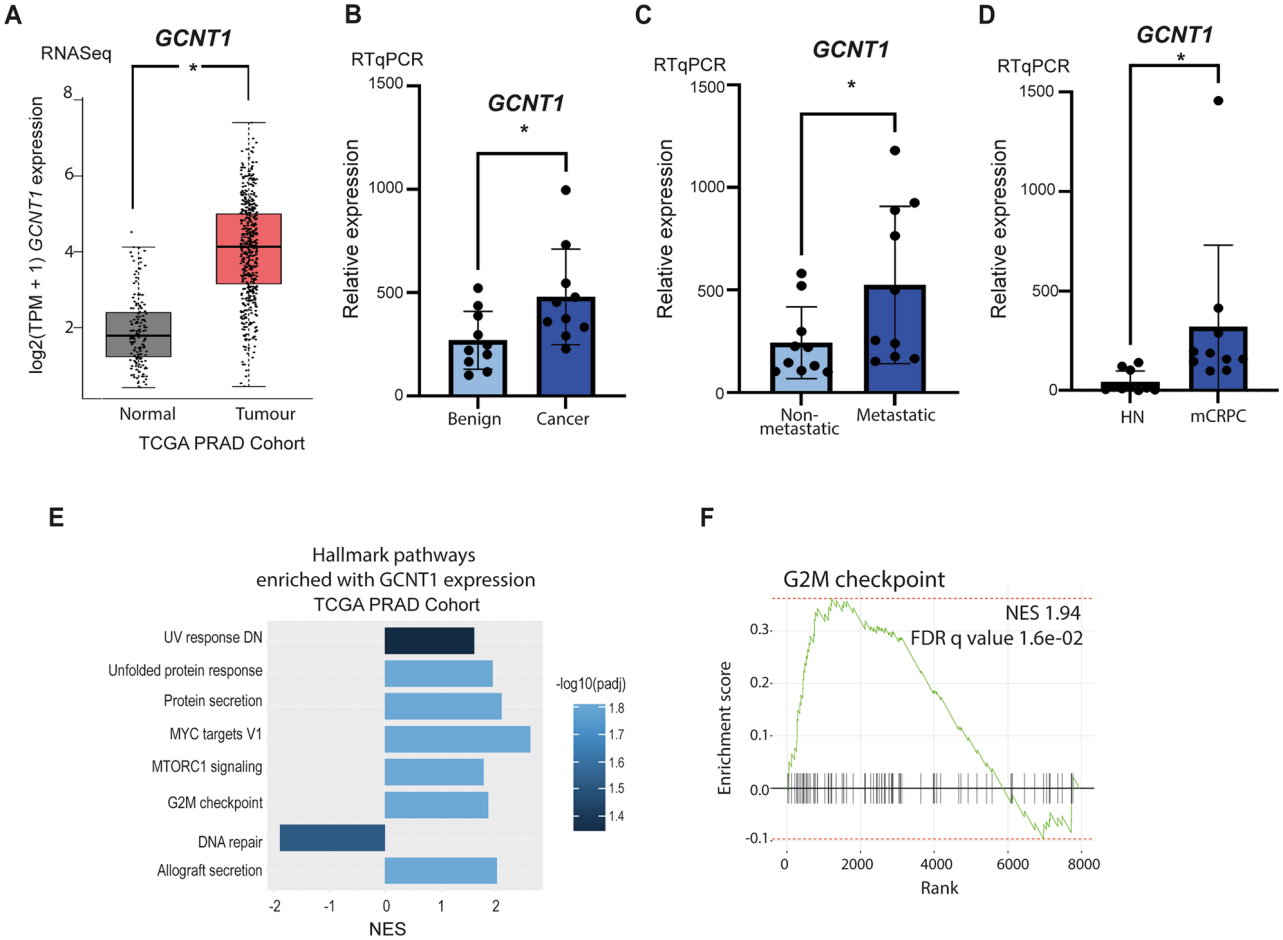
GCNT1 is upregulated in aggressive prostate cancer tissue. Analysis of *GCNT1* gene levels in clinical prostate tissue. (**A**) *GCNT1* mRNA levels in the TCGA PRAD cohort^31^ are significantly higher in prostate cancer tissue relative to normal prostate tissue (n = 544, p = 0.021). (**B**) Real-time PCR analysis of *GCNT1* in RNA samples extracted from FFPE prostate tissue. *GCNT1* is significantly upregulated in cancer relative to benign tissue (n = 20, unpaired t test, p = 0.0248). (**C**) Analysis of *GCNT1* mRNA levels in samples from the Walker et al.^33^ cohort. *GCNT1* is significantly upregulated in patients presenting with a ‘metastatic’ phenotype relative to patients with a ‘non-metastatic’ phenotype (n = 20, unpaired t test, p = 0.0494). (**D**) Real-time PCR analysis of *GCNT1* mRNA in hormone naïve prostate cancer and mCRPC tissue samples. *GCNT1* is significantly upregulated in mCRPC tissue samples relative to hormone naïve prostate cancer (n = 20, unpaired t test, p = 0.0481). (**E,F**) In the TCGA PRAD cohort (n = 493)^31^, *GCNT1* mRNA levels correlate with 8 hallmark response pathways, including ‘G2M checkpoint’ (NES 1.94, FDR q value 1.6e−02).

The glycosyltransferase GCNT1 has been previously identified as upregulated in prostate cancer and correlates with aggressive disease at both the gene and protein level^12,15,16,21–23^. Here, we monitor *GCNT1* gene expression in four additional prostate cancer clinical cohorts. Analysis of RNA sequencing data from The Cancer Genome Atlas Prostate Adenocarcinoma (TCGA PRAD) cohort^31,32^ reveals *GCNT1* gene expression levels are 2.3-fold higher in prostate tumours relative to normal prostate tissue (n = 544, p = 0.021) (Fig. 1A). Using quantitative PCR, we further show the *GCNT1* gene is 1.8-fold upregulated in prostate cancer tissue relative to benign prostate hyperplasia in an additional patient cohort (n = 20, p = 0.0248) (Fig. 1B). Next, we monitored *GCNT1* gene levels in a molecular subgroup of prostate cancer patients with metastatic potential at presentation (previously published by^33,34^). Within this dataset, *GCNT1* is 2.6-fold upregulated in the ‘metastatic subgroup’ compared to the ‘non-metastatic’ sub-group, suggesting upregulation of the *GCNT1* gene in primary prostate cancer patients presenting with metastatic biology (n = 20, p = 0.0494) (Fig. 1C). Furthermore, *GCNT1* gene levels are 7.5-fold higher in metastatic CRPC compared to hormone naïve disease (n = 20, p = 0.0481) (Fig. 1D). Our findings confirm upregulation of *GCNT1* gene levels in prostate cancer tissue compared to normal or benign prostate tissue, and suggest *GCNT1* may be upregulated in patients with increased risk of metastasis and those developing relapse to castrate resistant disease. Next, we analysed *GCNT1* gene expression in primary prostate cancer tissue samples from the TCGA PRAD clinical cohort (n = 493), and identified tumours with high (n = 124) and low (n = 123) *GCNT1* expression^31,35,36^. Fast gene set enrichment analysis (fgsea)^37^ of genes differentially expressed by tumours with high *GCNT1* expression vs low *GCNT1* expression identified significant enrichment of 8 hallmark pathways in tumours with high *GCNT1* expression (FDR q value < 0.05) (Fig. 1E). Of particular interest, when *GCNT1* is highly expressed in primary prostate cancer tissue there is significant enrichment of the hallmark pathway ‘G2M checkpoint’, which is associated with the trajectory of prostate cancer progression^38^ (NES 1.94, FDR q value 1.6e−02) (Fig. 1F).

### Upregulation of GCNT1 modifies the prostate cancer glycome

**Figure 2:**
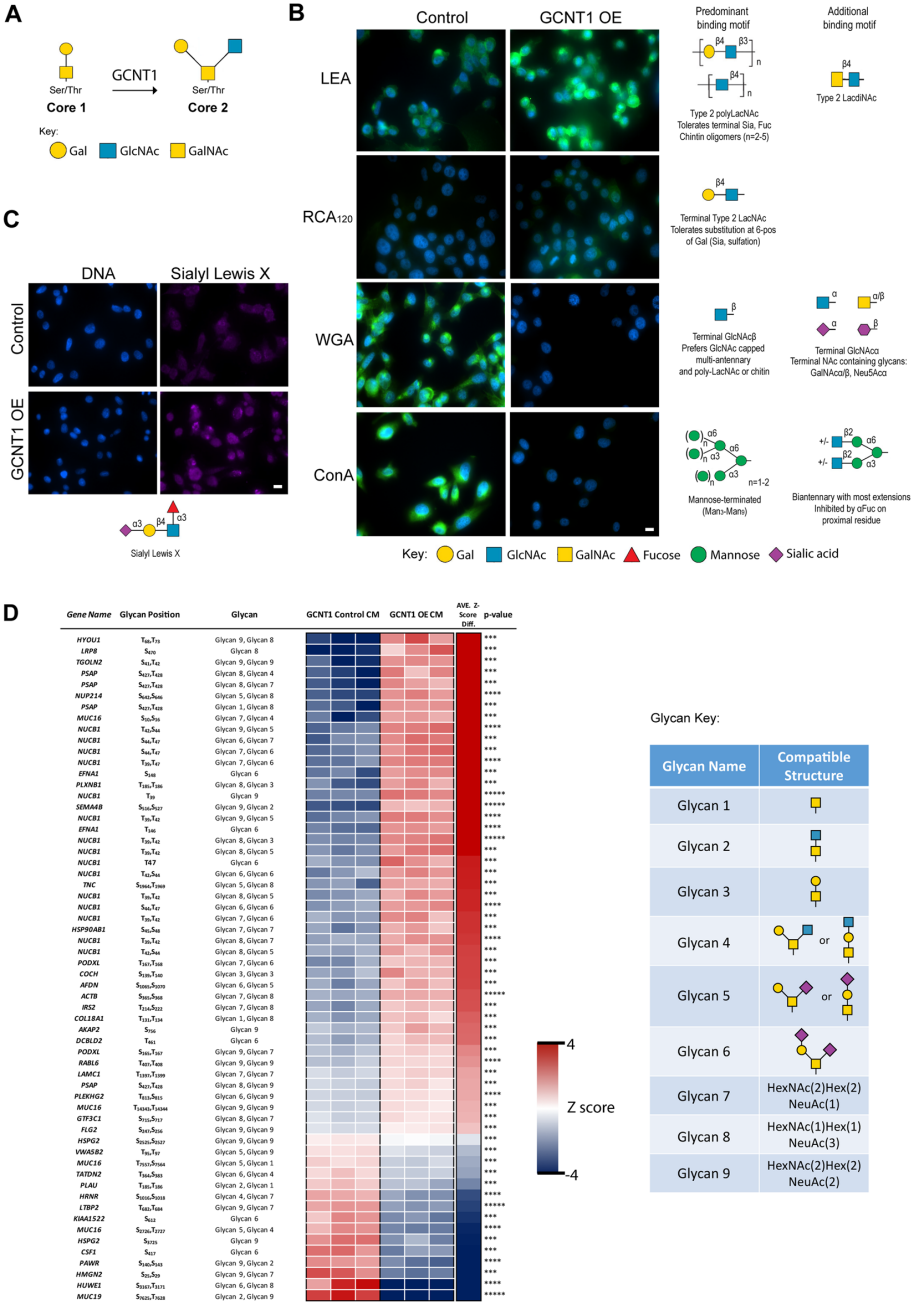
GCNT1 modifies the prostate cancer glycome. (**A**) GCNT1 is a key enzyme for the formation of core 2 linked *O*-glycans. In *O*-glycan synthesis GalNAc is transferred to Ser and Thr residues in the polypeptide, which can then be further extended with various carbohydrates. Four common *O*-glycan structures are expressed in mammalian tissues, core 1 through core 4. GCNT1 adds GlcNAc to GalNAc to form the core 2 branch. The core 2 branch is a scaffold for the production of lactosamine disaccharide repeats (specifically poly-*N*-acetyllactosamine) on *O*-glycans. (**B**) Lectin immunofluorescence shows PC3 cells with upregulated GCNT1 have increased levels of core 2 branched *O*-glycans (detected by enhanced binding of LEA and RCA_120_ lectins^39,40^), reduced levels of mannose terminated and biantennary structures respectively (detected using ConA lectin^40^) and reduced levels of terminal GlcNAc (detected using WGA lectin^40^). (**C**) Detection of SLe^X^ in PC3 cells using a glycan specific binding antibody^41^ indicates upregulation of GCNT1 promotes increased expression of the SLe^X^ antigen in prostate cancer cells. (**D**) Glycoproteomics to map GCNT1 glycosylation sites on specific proteins in DU145 prostate cancer cells with upregulated GCNT1 identified 60 glycopeptides as statistically significant (p < 0.001) specific substrates of GCNT1. Putative glycan structures in this figure were created using the DrawGlycan-SNFG tool^85^.

GCNT1 catalyses the formation of core 2 branched *O*-glycan structures on polypeptides^19^ (Fig. 2A). In prostate cancer, GCNT1 has previously been linked to higher levels of core 2 *O*-linked Sialyl Lewis X (sLeX) on PSA, PAP, and MUC1 proteins^22^, however how GCNT1 impacts global glycosylation in prostate cancer cells has not been previously studied. To test if upregulation of GCNT1 alters the cell surface glycosylation of prostate cancer cells, we monitored the recognition by lectins and glycan-binding antibodies in PC3 prostate cancer cells overexpressing GCNT1 (PC3 cells were chosen for these experiments as they have low levels of endogenous GCNT1 expression, Supplementary Fig. 1). Consistent with increased levels of core 2 branched *O*-glycans, lectin immunofluorescence revealed overexpression of GCNT1 correlates with enhanced binding of LEA lectin (which binds GlcNAc oligomers, polyLacNAc, and/or chitin) and RCA_120_ lectin (which predominantly recognises terminal type 2 LacNAc)^39,40^. However, in line with previous studies^19^ in addition to detecting changes to core 2 *O*-glycans, we also observed additional changes in the repertoire of glycans detected in GCNT1 overexpressing cells. GCNT1 overexpression also correlated with decreased binding of WGA lectin (which recognises terminal GlcNAc), and decreased binding of ConA lectin (which recognises mannose terminated and biantennary structures respectively)^40^ (Fig. 2B and Supplementary Fig. 2). The formation of the core 2 branch is critical for the biosynthesis of sLe^X^ in *O*-glycans. To monitor expression of the sLe^X^ structure, we used a specific glycan binding antibody^41^ to assess prostate cancer cells overexpressing GCNT1^42^. Our findings suggest increased GCNT1 expression correlates with upregulation of the cancer-associated sLe^X^ glycan on the cell surface, which is a functional ligand for Selectins^43–45^ (Fig. 2C and Supplementary Fig. 2). Next, we analysed the *O*-glycoproteome in secretomes of DU145 prostate cancer cells with upregulated GCNT1 using mass spectrometry. This identified 60 glycopeptides as potential substrates for GCNT1 in prostate cancer cells, including a potential increase in core 2 *O*-glycan structures on PSAP and MUC16 (Fig. 2D). Together, these experiments suggest that GCNT1 may impact the phenotype of prostate cancer cells by modifying both cell surface glycosylation and the *O*-glycosylation of proteins secreted by prostate cancer cells.

### GCNT1 promotes prostate tumour growth in vivo

**Figure 3:**
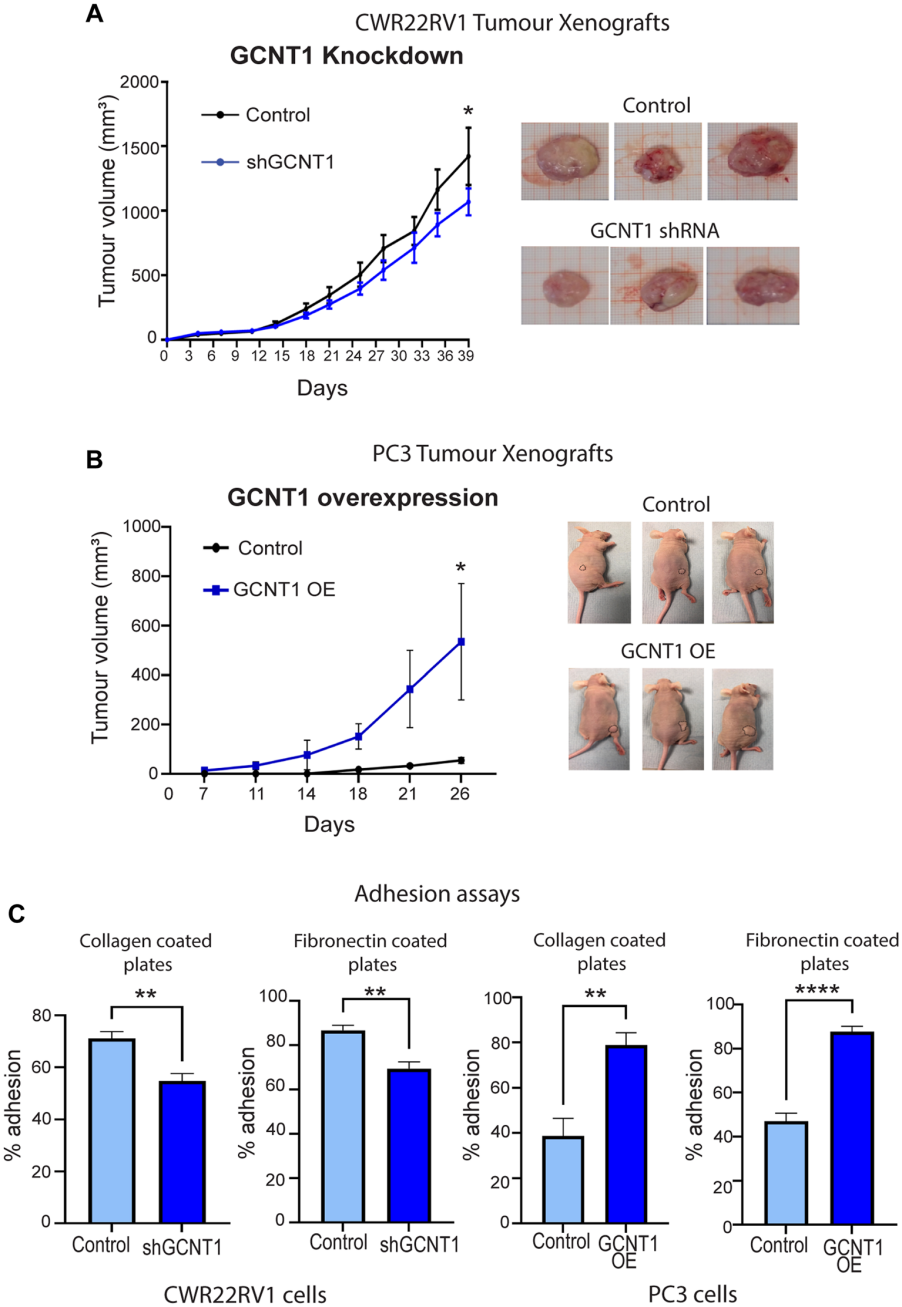
GCNT1 promotes prostate tumour growth in vivo. (**A**) Knockdown of GCNT1 using shRNA significantly reduces the growth of CWR22RV1 tumours xenografts in a subcutaneous xenograft model. (**B**) Upregulation of GCNT1 in PC3 cells significantly increases the growth of subcutaneous xenograft tumours. (**C**) Knockdown of GCNT1 significantly reduces the adhesion of CWR22RV1 cells to collagen and fibronectin coated plates, whereas overexpression of GCNT1 in PC3 cells has the opposite effect.

We next investigated the impact of GCNT1 expression on the biology of prostate cancer cells using both in vitro and in vivo assays. Our in vitro studies show knockdown of GCNT1 suppresses prostate cancer cell proliferation and colony formation, whereas overexpression of GCNT1 had the opposite effect (Supplementary Fig. 3). Consistent with this, knockdown of GCNT1 using sub-cutaneous in vivo mouse models significantly reduces the growth of CWR22RV1 tumours (Fig. 3A), whereas overexpression of GCNT1 significantly increases the growth of PC3 tumours (Fig. 3B). These findings are consistent with previous studies showing that upregulation of GCNT1 enhances the growth of orthotopic LNCaP tumours^29^. The effect of GCNT1 levels on prostate cancer cell adhesion was also investigated using cell adhesion assays. The results show that knockdown of GCNT1 in CWR22RV1 cells significantly decreased cell adhesion to both collagen and fibronectin coated plates, whereas overexpression of GCNT1 in PC3 cells significantly increased cell adhesion (Fig. 3C).

### Upregulation of GCNT1 alters oncogenic signalling pathways in prostate cancer cells

**Figure 4:**
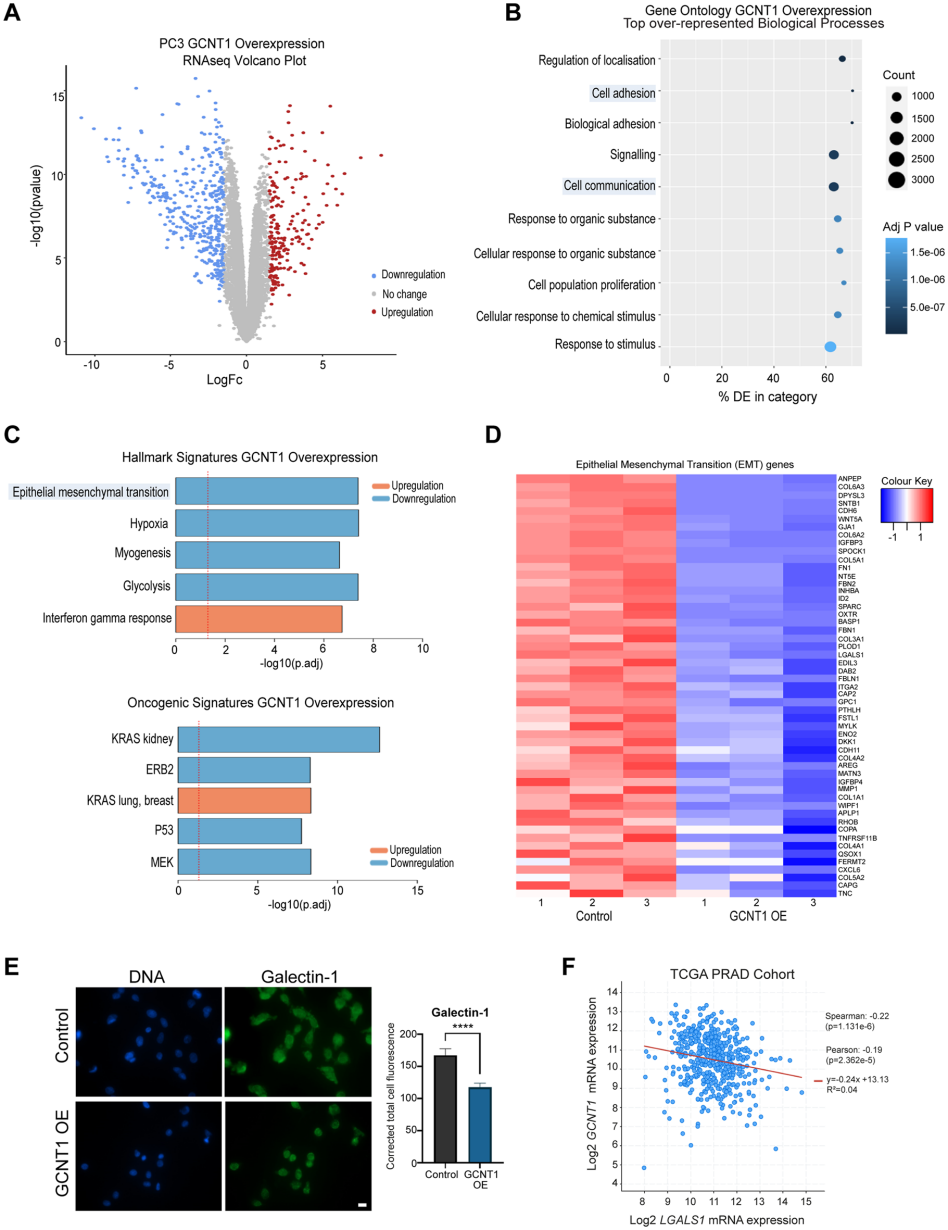
Upregulation of GCNT1 alters oncogenic gene expression pathways in prostate cancer cells. (**A**) RNA-seq analysis of PC3 cells with upregulated GCNT1 identified 2445 genes that are differentially expressed in response to GCNT1. (**B**) Gene Ontology analysis of genes regulated by GCNT1 shows an enrichment of genes with roles in ‘signalling’ and ‘cell communication’. (**C**) Ensemble Gene Set Enrichment Analysis of genes regulated by GCNT1 reveals downregulation in the ‘epithelial mesenchymal transition’ hallmark signature, and downregulation in ‘P53’ and ‘MEK’ oncogenic signatures. (**D**) Heatmap to illustrate 53 genes with roles in EMT that are significantly downregulated in GCNT1 overexpressing cells. (**E**) Validation at the protein level shows Galectin-1, which is encoded by the *LGALS1* gene, is downregulated when GCNT1 is overexpressed. (**F**) Analysis of the TCGA PRAD cohort (n = 493)^31,35,36^ shows a significant correlation between *GCNT1* and *LGALS1* mRNA levels in primary prostate cancer clinical tissue.

To further investigate the role of GCNT1 in prostate cancer cells, we used RNA-sequencing to search for gene expression pathways that change with upregulation of GCNT1. Bioinformatic analyses identified 2445 differentially expressed genes when GCNT1 is overexpressed in PC3 cells (adjusted p value < 0.05, Log_2_FC 0.58) (Fig. 4A and Supplementary Table 1) with enrichment in ‘cell adhesion’ and ‘cell communication’ biological processes (Fig. 4B) and downregulation in the ‘epithelial mesenchymal transition (EMT)’ hallmark signature (Fig. 4C). Visualisation of significantly differentially expressed EMT hallmark genes in a heatmap confirmed that GCNT1 overexpression results in downregulation of the EMT pathway (Fig. 4D). Of particular interest in the EMT gene set, the *LGALS1* gene, which encodes Galectin-1 (a carbohydrate binding protein implicated in modulating cell–cell and cell–matrix interactions^46–53^), was significantly downregulated when GCNT1 is overexpressed (adjusted p value < 0.01, Log_2_FC − 0.7). Validation at the protein level confirmed that upregulation of GCNT1 in prostate cancer cells promotes loss of Galectin-1 (Fig. 4E) and analysis of The Cancer Genome Atlas Prostate Adenocarcinoma (TCGA PRAD) cohort^31,35,36^ revealed a correlation between the *GCNT1* and *LGALS1* genes in clinical prostate cancer tissue (Fig. 4F). Our data also reveals potential links between GCNT1 and the alteration of several other pathways important in prostate cancer, including MEK signalling which is a therapeutic target for mCRPC^54^, ERBB2 signalling which is also believed to contribute to prostate cancer progression^55,56^, and altered KRAS signatures which are a major player in promoting and maintaining tumorigenesis^57^. Together, these findings reveal GCNT1 overexpression can alter oncogenic gene expression pathways in prostate cancer that are important in disease progression.

## Discussion

In prostate cancer, aberrant glycosylation is closely linked to a malignant phenotype, but the mechanisms behind how altered glycosylation contributes to disease pathology are poorly understood. Here, we show that GCNT1, which encodes a key glycosyltransferase enzyme controlling *O*-glycan branching, is upregulated in aggressive prostate cancer tissue. In line with previous reports, our study shows that increased expression of GCNT1 promotes the growth of prostate tumours, and building on these findings we also reveal GCNT1 can increase cell adhesion. Furthermore, we find that upregulation of GCNT1 can alter the glycome of prostate cancer cells to increase levels of core 2 *O*-glycans and upregulate expression of sLe^X^, and can impact oncogenic gene expression patterns.

Previous studies show that upregulation of GCNT1 correlates with higher levels of core 2 *O*-sLe^X^ on PSA, PAP, and MUC1 proteins^22^, but how GCNT1 modifies global glycosylation in prostate cancer cells has not been studied. This study shows overexpression of GCNT1 in prostate cancer cells modifies cell surface glycosylation and leads to increased levels of core 2 *O*-glycan structures, including upregulation of the poly-*N*-acetyllactosamine chain (which serves as a scaffold for terminal modifications such as the tumour-associated glycan sLe^X^). We also find GCNT1 can upregulate the sLe^X^ structure, a Selectin ligand with a key role in metastasis^43–45,58,59^. Using site specific *O*-glycoproteomics, we identified 60 potential substrates for GCNT1 in the prostate cancer secretome. It is interesting to speculate that the role of GCNT1 in prostate cancer tumour growth and control of gene expression patterns could be due to some of its protein substrates’ roles or combined functions. Taken together, these studies provide insights into how upregulation of GCNT1 can modify the prostate cancer glycome, however additional studies will be needed to further identify and characterise the polypeptide substrates of GCNT1.

Several reports have correlated *GCNT1* expression in prostate cancer with aggressive disease, including recurrence after surgery^23^ and the spread of cancer cells outside of the prostate gland^21^. It has also been demonstrated that GCNT1 enhances the growth of orthotopic prostate tumours^29^. Consistent with previously published studies, the findings reported here also correlate *GCNT1* gene expression levels with more aggressive prostate cancer and show upregulation of GCNT1 increases the growth of prostate tumours. Our findings indicating upregulation of GCNT1 correlates with downregulation of the EMT hallmark pathway may provide insight into how GCNT1 can alter the adhesive properties of prostate cancer cells and potentially lead to a reduction in the seeding potential of prostate cancer cells during the metastatic process. Indeed, using an intra-cardiac injection model, we find that upregulation of GCNT1 in PC3 cells significantly reduces the number of metastatic tumours formed (p = 0.0295) (Supplementary Fig. 4). The complex biological process of EMT has been heralded as a key hallmark of cancer that confers metastatic properties upon cancer cells^60^. There is considerable evidence to suggest that the transition of epithelial cells to a mesenchymal state via EMT promotes an invasive phenotype that contributes to prostate cancer metastasis^61–63^. However, studies using pre-clinical models suggest the transition between epithelial and mesenchymal phenotypes represents a dynamic and complex set of events that play important roles in metastatic dissemination and colonisation^64^ and that the transient induction of EMT and the subsequent reversion to an epithelial phenotype (known as mesenchymal to epithelial transition or MET) may be necessary for the formation of overt metastasis^64–68^. Our findings indicating upregulation of GCNT1 correlates with downregulation of the EMT hallmark pathway may provide insight into how GCNT1 can alter the adhesive properties of prostate cancer cells and lead to a reduction in the seeding potential of prostate cancer cells during the metastatic process. Therefore, it is important to consider our data in the context of the complex and dynamic role EMT likely plays in aggressive prostate cancer.

Taken together, our findings reveal potential mechanisms to explain how upregulation of GCNT1 might contribute to the prostate cancer pathology, however further studies will be needed to further unpick the mechanisms involved. In particular, while the data presented here examines the role of GCNT1 in the formation of metastatic tumours by disseminated prostate tumour cells, to fully study the in vivo role of GCNT1 in prostate cancer metastasis it will be necessary to utilise a repertoire of mouse models encompassing different stages in the metastatic cascade. In addition, while the in vivo studies reported here were carried out using immunocompromised mouse models, moving forward, and as GCNT1 can upregulate sLe^X^ (a Selectin ligand linked to tumour metastasis which is recognised by endothelial cells and lymphocytes^43–45,58,59^), it will be also important to study the role of GCNT1 in prostate cancer metastasis in the context of a functional immune system.

## Methods

### RT-qPCR

RNA extraction, cDNA synthesis and real-time PCR were as described previously^34^.

### Clinical RNA cohorts

The clinical RNA samples shown in Fig. 1B were collected from Castle Hill Hospital (Cottingham, Hull) and used with ethical approval by the local Research Ethics Committee (Ethics Number: 07/H1304/121). Patients gave informed consent and all patient samples were anonymised. The clinical RNA samples shown in Fig. 1C and D were obtained via the Prostate Cancer Biorepository Network (PCBN). Our study was peer reviewed and approved by PCBN. Samples were prepared and stored using standard protocols and all patients gave informed consent. All methods were performed in accordance with the relevant guidelines and regulations.

### Cell culture and creation of stable cell lines

Cell culture and the cell lines used were as described previously^34^. The stable cell lines used in the study were created using lentiviral transduction. For knockdown of GCNT1 the following shRNA lentiviral particles were purchased from Santa Cruz (GCNT1 shRNA sc-92945-V and Control shRNA sc-108080). Transductions were carried out according to the manufacturer’s instructions using an MOI of 5. For overexpression of GCNT1 the following Lentifect Purified lentiviral particles were purchased from Tebu-Bio (GCNT1 LPP-Z6088-Lv242-050-S and Negative Control 217LPP-NEG-Lv242-025-C). Transductions were carried out according to the manufacturer’s instructions using an MOI of 5. For the metastasis study, PC3 cells were transduced with Firefly Luciferase Lentivirus (BPS Bioscience, 79692-H) at a MOI of 1 in media containing 5 µg/ml polybrene. Stable cell lines were selected with 200 μg/ml hygromycin, and then further selected for GCNT1 overexpression using the methods described above.

### Colony formation assays

Cells were plated at a density of 100 cells per 100 mm dish and maintained until colonies of more than 50 cells/colony had formed. Cells were fixed in 10% formalin for 10 min and stained with 0.5% crystal violet for 10 min at room temperature. Colony numbers were counted manually. Three biological repeats were conducted per cell line.

### Adhesion assays

Cell adhesion assays were performed using the Vybrant Cell Adhesion Assay Kit (Fisher, V-13181) using both collagen coated plates (Sigma, CC304) and fibronectin coated plates (Sigma, S3815). 50,000 cells per well were allowed to adhere for two hours and assays were performed as per the manufacturer’s instructions. Percentage adhesion was calculated by comparing the absorbance with that of an identical unwashed plate. Three biological repeats were conducted per cell line.

### Mouse models

#### CWR22RV1 tumour xenografts

Male NMRI mice (Charles Rivers) were inoculated at 8 weeks of age with 1 × 10^7^ CWR22RV1 cells with GCNT1 knockdown by unilateral subcutaneous injection into the flank. Cells were injected in a volume of 100 µL and Matrigel in a 1:1 mixture. Animals were weighed and tumour volumes monitored by caliper measurement three times a week until the first animal met a humane endpoint.

#### PC3 tumour xenografts

Male CD-1 Nude mice (Charles Rivers) were inoculated at 8 weeks of age with 1 × 10^7^ PC3 cells with GCNT1 overexpression. Cells were injected in a volume of 50 µL of cell culture media and Matrigel in a 1:1 mixture. Animals were weighed and tumour volumes monitored by caliper measurement three times a week until the first animal met a humane endpoint.

### PC3 tumour engraftment study

Six-week old male BALB/cAnNCrl immunocompromised (athymic nude) mice were purchased from Charles River (Kent, UK) and housed in a controlled environment in Optimice cages (Animal Care Systems, Colorado, USA) with a 12 h light/dark cycle at 22 °C with ad libitum water and 2018 Teklad Global 18% protein rodent diet containing 1.01% Calcium (Harlan Laboratories, UK). Mice were randomised into two groups to receive single-cell suspensions of 1 × 10^5^ PC3 (Control or GCNT1 OE) cells/100 μL PBS via injection into the left cardiac ventricle of mice (intracardiac injection). Tumour progression was monitored weekly based on bioluminescence using the in vivo imaging systems (IVIS, PerkinElmer, Cambridge, UK) for 6 weeks and the images were blinded for data analysis.

Mice were euthanized through exsanguination under general anaesthesia, followed by cervical dislocation. All procedures complied with the UK Animals (Scientific Procedures) Act 1986 and were reviewed and approved by the local Research Ethics Committees of the University of Sheffield under Home Office project licence (PP3267943). The animal research performed in this study is reported in accordance with ARRIVE guidelines.

### Immunofluorescence

Cells were cultured in Lab-Tek™II Chamber Slides (ThermoScientific, 154453) for 48 h. Cells were washed with PBS before permeabilization and fixation with ice-cold absolute methanol for 10 min at – 20 °C. Next, slides were washed with PBS and blocked with Carbo-Free™ Blocking solution (Vector Laboratories, SP-5040) for 1 h at room temperature. Slides were incubated overnight at 4 °C with FITC-conjugated LEA (Lectin from *Lycopersicon esculentum*, Sigma, L0401), RCA_120_ Fluorescein (*Ricinus Communis* Agglutinin 120, Vector Laboratories, FLK-2100), WGA fluorescein (Wheat Grain Agglutinin, Vector Laboratories, FLK-2100), ConA fluorescein (Concanavalin A, Vector Laboratories, FLK-2100), CoraLite^®^488-conjugated Galectin-1 Monoclonal antibody (Proteintech, CL488-60223) at 1:200, and Sialyl Lewis X (Alexa Fluor 647 Rat Anti-Human Cutaneous lymphocyte antigen, BD Pharmagen, 563533) at 1:100. Finally, slides were washed with PBS and stained with Hoechst (ThermoScientific, 62249) for 15 min at room temperature. Images were acquired and processed with the ZEISS Axio Imager 3 microscope. The fluorescent images were analysed using NIH ImageJ software^69^ (Version t1.51) by measuring the area, integrated density and mean grey value of one cell at a time (*n* = 50). The fluorescence intensity was calculated in Excel using the formula for corrected total cell fluorescence (CTCF) = integrated density – (area of selected cell × mean fluorescence of background readings).

### Mass spectrometry-based glycoproteomics

Cell pellets were lysed using 500 µL of 1.5% SDS lysis solution containing 1X protease inhibitors (Sigma Aldrich) followed by sonication using a Branson probe sonicator (Fisher Scientific). Conditioned cell media was concentrated to a final volume of 500 μL using 10 kDa 15 mL spin filters (Amicon). Protein concentration was quantified with using a standard BCA protein assay (Thermo Fisher Scientific) following the manufacturer’s protocol. 300 µg of proteins from the cell lysates and media were aliquoted out into a new 1.5 mL tube (Eppendorf). The sample volume was brought up to 100 µL using 50 mM ammonium bicarbonate (Sigma Aldrich) followed by vortexing for 15 s. Samples were incubated for 1 h at 65 °C with 10 mM Tris(2-carboxyethyl) phosphine hydrochloride (TCEP, Sigma Aldrich) to reduce the cysteine disulfide bonds. Then, thiolate groups on cysteines were alkylated with 15 mM of iodoacetamide (Across Organics) in the dark for 45 min at room temperature. Proteins were precipitated with 1 mL of cold acetone and stored at − 20 °C overnight. Precipitated proteins were pelleted down via centrifugation at 14,000*g* for 10 min at 4 °C and acetone was removed. Pelleted proteins were dried at room temperature and 50 µL of 50 mM ammonium bicarbonate. Proteins were digested with sequencing grade trypsin (Thermo Fisher Scientific) at a 1:30 enzyme to protein ration and incubated at 37 °C overnight without shaking.

Glycopeptides were enriched using the strong anion exchange and electrostatic repulsion hydrophilic interaction chromatography (SAX-ERLIC)^70,71^. SOLA SAX SPE (Thermo Fisher Scientific) columns were washed with 3 mL of acetonitrile with a flow rate 1 mL/min followed by 3 mL of 100 mM triethylammonium acetate in water, and 3 mL of 1% TFA in water. The column was equilibrated with 3 mL of 95% acetonitrile with 1% TFA in water. The organic solvent in the tryptic peptide solution was adjusted by adding 3 mL of 95% acetonitrile with 1% TFA in water. Samples were loaded on the SOLA-SAX SPE column and passed through with a flow rate of 0.5 mL/min. The SOLA-SAX SPE column was then washed with 6 mL of 95% acetonitrile 1% formic acid in water. Glycopeptides were eluted in a 2.0 mL Eppendorf vials by adding 850 µL aliquots of 50% acetonitrile 0.1% TFA in water twice, followed by another two 850 µL aliquots of 5% acetonitrile 0.1% TFA in water. The two glycopeptide fractions were concentrated, combined, and dried down using a speed vacuum (LabConco) prior to LC-MSMS analysis.

3 µL of the glycopeptides were loaded into a 20 µL sample loop using a Dionex Ultimate Rapid Separation liquid chromatography system (Thermo Fisher Scientific) and subsequently loading glycopeptides onto a C18 PepMap trap column (Thermo Fisher Scientific) at a flow rate of 5 µL/min for 10 min. A reversed phase liquid chromatography gradient consisting of mobile phase A (0.1% formic acid in water) and mobile phase B (0.1% formic acid in acetonitrile) was used to separate glycopeptides on a 25 cm long analytical column packed with BEH C18, 130 Å, and 1.7 µm particle size (Waters) encapsulated in a column heater (MSWILL) with a temperature set at 60 °C. Eluted peptides were subject to MS/MS analysis on an Orbitrap Eclipse Tribrid mass spectrometer (Themo Fisher Scientific). The gradient program was set to hold mobile phase B at 2% for the first 6 min, steadily ramped to 35% B over the next 80 min followed by an increase to 85% B over 5 min with a 5 min hold at a flow rate of 0.3 µL/min and a 15 min equilibration time. Each sample was analysed in triplicate. The MS program consisted of setting the cycle time at Top-speed at 1 s with an MS mass scan range of 450–2000 m/z and mass resolution of 50,000. The most abundant precursor ions were fragmented with Higher Energy Collisional Dissociation (HCD) with a collisional energy set to 38%. Dynamic exclusion was enabled for 60 s with a mass tolerance of 10 ppm and the normalized AGC target set to 100%. MS2 fragments were detected in the Orbitrap detector with mass scan range set to auto and a mass resolution of 30,000. The injection time set to custom with the maximum injection time of 54 ms. The raw data files were searched using Byonic v3.9.6 software against a focus protein database identified from the shotgun analysis from the same sample set (2020; 4419 entries) and a glycan database of 9 common O-glycans provided by Byonic software. The glycopeptide search included parameters of trypsin digestion with maximum two missed cleavages, MS1 precursor mass tolerance of 10 ppm, and fragment mass tolerance 0.5 Da with fixed cysteine carbamidomethylation, variable methionine oxidation and asparagine deamination. To further validate glycopeptide identifications, the following process was applied. Only glycopeptides with > 6 amino acids were retained. A theoretical MS/MS spectrum was generated for each of the remaining glycopeptides. Spectral matching was then performed so that peptides with 6–7, 8–16, and 17–20 amino acids needed to have 80, 70, and 50% sequence coverage in the measured MS/MS fragmentation spectra respectively to be considered for further analysis.

After aligning the chromatographic runs, quantitative data was extracted from the MS1 spectra of identified peptides using an R script based on the MSnbase package^72^. This approach allowed for the retrieval of precise measurements of peptide abundance. The area under the curve (AUC) of the extracted ion current (XIC) was calculated for all detected peptides, providing quantitative measurements. To analyse changes in protein abundance, the Generic Integration Algorithm was employed. This algorithm utilized the AUC values of the peptides to assess variations in protein expression between two groups: the GCNT1 overexpression group and the control DU145 group. Following a previously described approach^73^, all quantitative data was expressed as Z-scores at the protein or peptide level. Log2 ratios were calculated by comparing the AUC of peptides in the GCNT1 overexpression group with the control DU145 group, enabling the determination of fold changes in protein expression. The WSPP model was applied at the spectrum level to calculate the corresponding statistical weight. The weight was rescaled and standardized to a normal distribution N(0,1), facilitating comparisons and enabling the identification of statistically significant changes. Cumulative distribution plots were generated at each level (spectrum, peptide, and protein) to assess the validity of the null hypothesis and examine the statistical significance of observed differences. Initial analysis focused on unmodified peptides to determine variations at the scan, peptide, and protein levels, as well as changes in protein expression. Subsequently, glycan-containing peptides were included in the analysis to identify any peptides that deviated significantly from the behaviour of other non-modified peptides belonging to the same protein^73^. The final statistical comparison was performed using Student's t-test, with a significance level below 0.01 considered statistically significant. Only peptides meeting this criterion were selected for further analysis.

### RNA sequencing analysis

RNA sequencing data can be access on GEO repository (GSE224036). RNA was extracted from 3 PC3 cell lines with stable GCNT1 overexpression and 3 PC3 cell lines transduced with negative-control lentiviral particles. Samples were prepared with the Illumina TruSeq Stranded mRNA Library Prep Kit and sequenced using an Illumina NextSeq 500, giving 13 million 75 bp single reads per sample. All data analyses were performed in Galaxy version 22.01. Quality control was performed with FastQC (http://www.bioinformatics.babraham.ac.uk/projects/fastqc/) and reads were trimmed with Cutadapt^74^. Reads were mapped to hg38 using HISAT2^75^ and quantified with featureCounts^76^. Differential gene expression analysis was performed using limma-voom^77^ and a volcano plot was generated with ggplot2^78^. Gene ontology (GO) analysis was performed with goseq^79^ applying a significance threshold of adjusted p value < 0.05 for differentially expressed genes. Gene Set Enrichment Analysis (GSEA) was performed with the package EGSEA^80^. Normalised count matrix values were used to create a heatmap with gplots^81^.

### Bioinformatic analysis of TCGA cohort

To compare *GCNT1* gene expression levels in normal prostate and prostate tumour tissue samples, data from The Cancer Genome Atlas Prostate Adenocarcinoma (TCGA PRAD) cohort was analysed using GEPIA^31,32^. To profile gene expression patterns in the TCGA PRAD cohort, RNA-Seq data from prostate tumours (n = 493) was analysed in cBioPortal^31,35,36^. Tumours were ranked by *GCNT1* expression levels to delineate two groups from the top and bottom quartiles. Group comparison of 124 tumours with high *GCNT1* expression vs 123 tumours with low *GCNT1* expression revealed 8110 differentially expressed genes (q-value < 0.05). Genes were pre-ranked using the t statistic and GSEA was performed using the fgsea^37^ package in Galaxy version 22.05. Annotated gene sets were obtained from the Molecular Signatures Database (MSigDB)^82,83^ and a bar chart was generated from fgsea data in R version 4.2.1^84^ with ggplot2.

### Supplementary Information

Supplementary Information.

## Data Availability

The data that support the findings of this study are openly available and can be accessed on GEO repository (GSE224036).
