# Supplementary material for: The role of GCNT1 mediated O-glycosylation in aggressive prostate cancer
Source: Sci Rep. 2023 Oct 9;13:17031. doi: 10.1038/s41598-023-43019-8 (PMC10562493; doi:10.1038/s41598-023-43019-8)
Supplement: Supplementary file 1 — Supplementary Information. [file 41598_2023_43019_MOESM1_ESM.pdf]

Supplementary Figure 1. Validation of prostate cancer stable cell line models with knockdown or overexpression of GCNT1. (**A**) Real-time PCR analysis of *GCNT1* gene expression levels in LNCaP, CWR22RV1, PC3 and DU145 prostate cancer cells. (**B**) Real-time PCR and sandwich ELISA analysis of GCNT1 mRNA and protein levels in CWR22Rv1 cells with shRNA mediated knockdown of GCNT1. (**C**) Real-time PCR and sandwich ELISA analysis of DU145 cells with upregulation of GCNT1. (**D**) Real-time PCR, sandwich ELISA, and immunocytochemistry analysis of PC3 cells with overexpression of GCNT1. For real time PCR, the expression levels of *GCNT1* mRNA is shown relative to the expression of three housekeeping genes, *GAPDH*, *β–tubulin*, and *actin*.

Supplementary Figure 2. Quantification of lectin and antibody immunofluorescence images from Figure 2.

Supplementary Figure 3. (**A**) WST-1 cell proliferation assays show knockdown of GCNT1 in CWR22RV1 cells significantly reduces cell proliferation. Conversely, overexpression of GCNT1 in PC3 cells significantly increases cell proliferation. (**B**) Overexpression and knockdown of GCNT1 also alters prostate cancer cell colony formation.

Supplementary Figure 4. (**A**) Upregulation of GCNT1 in PC3 cells significantly reduced the number of metastatic tumours formed following intra-cardiac injection (p=0.0295).

Supplementary Table 1: List of differentially expressed genes in PC3 prostate cancer cells with upregulation of GCNT1

Supplementary Figure 1
Validation of GCNT1 knockdown and overexpressing stable cell lines

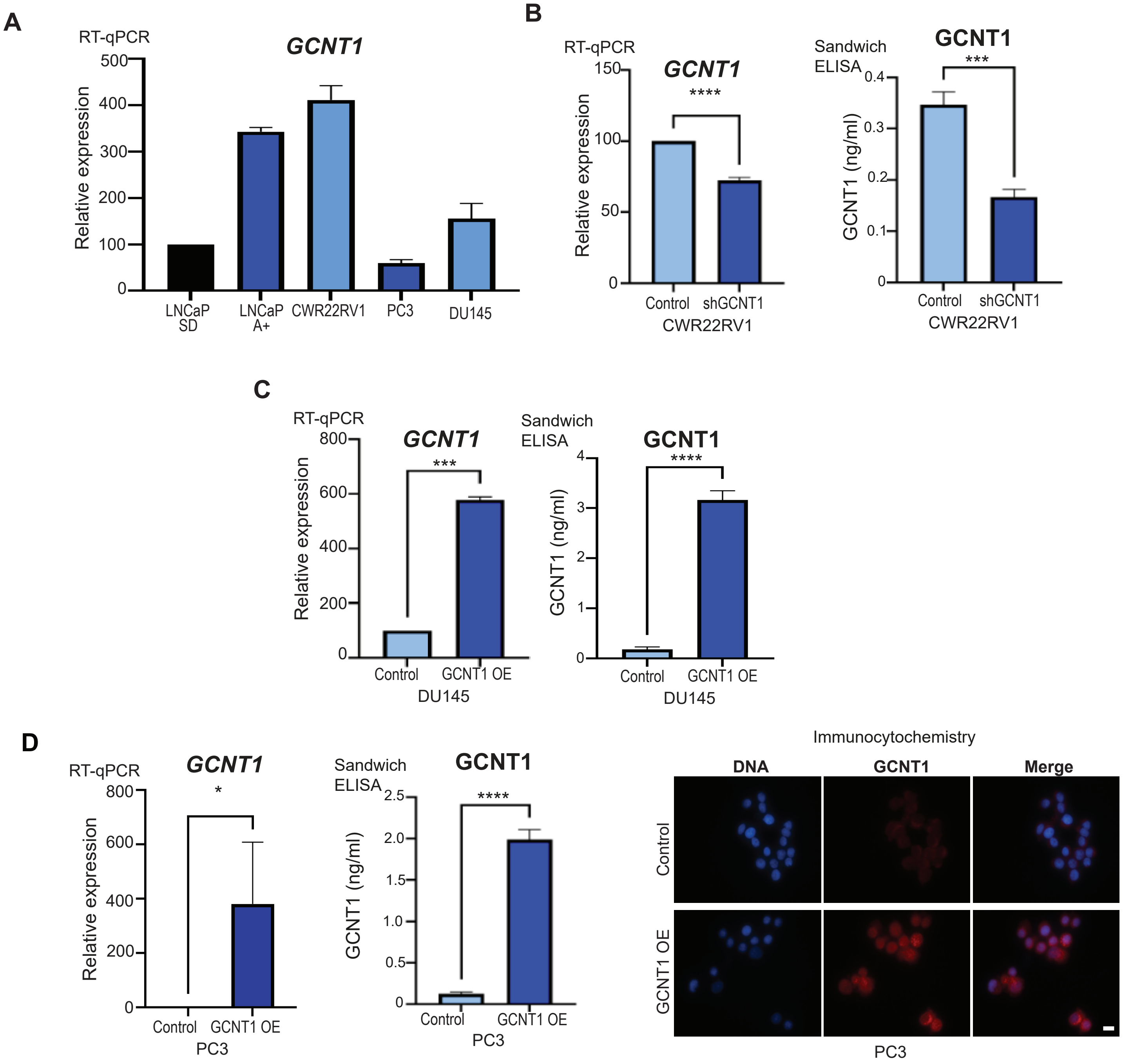

Supplementary Figure 2
Quantification of lectin and antibody immunofluorescence assays

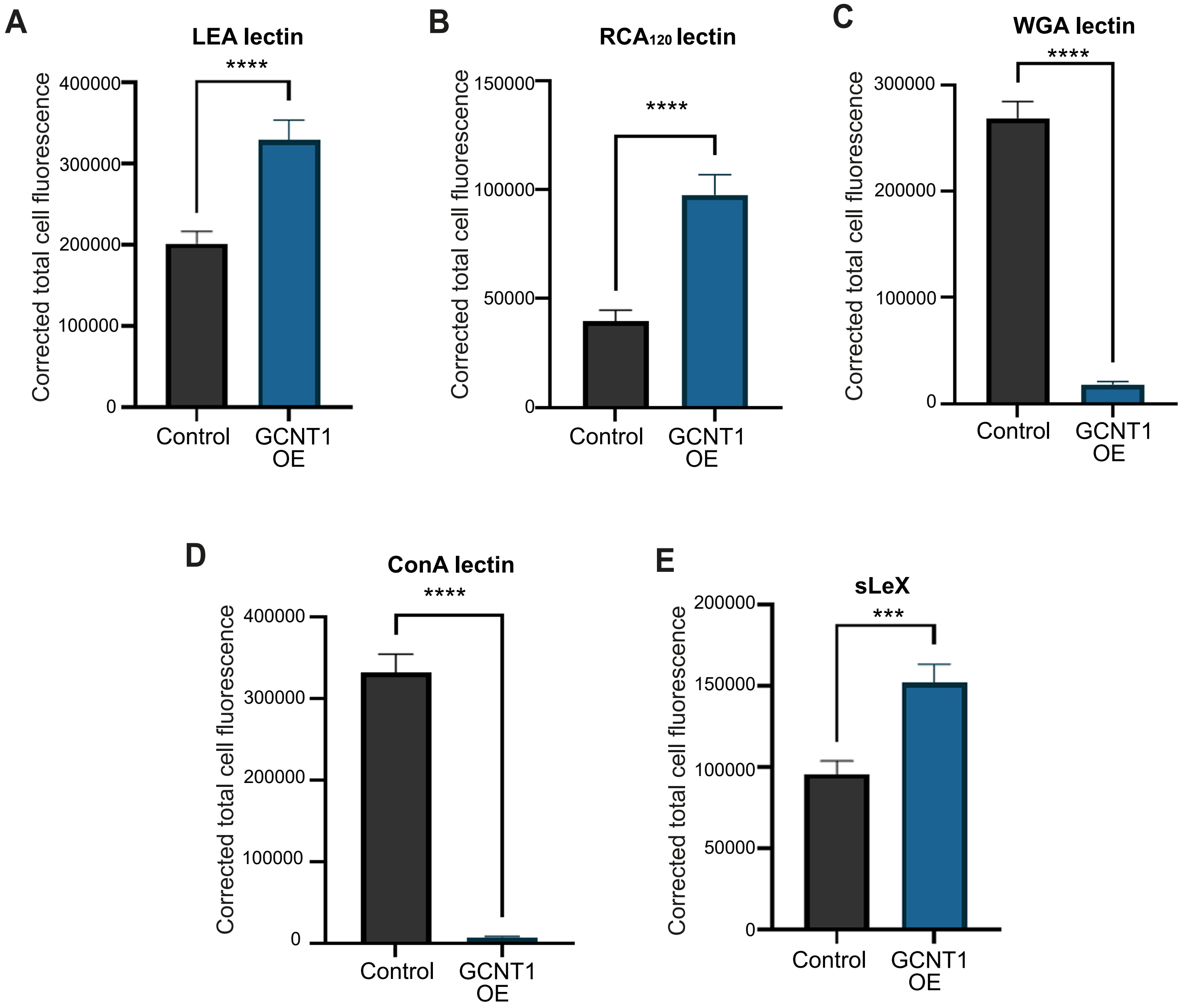

Supplementary Figure 3
GCNT1 promotes prostate cancer cell proliferation in vitro

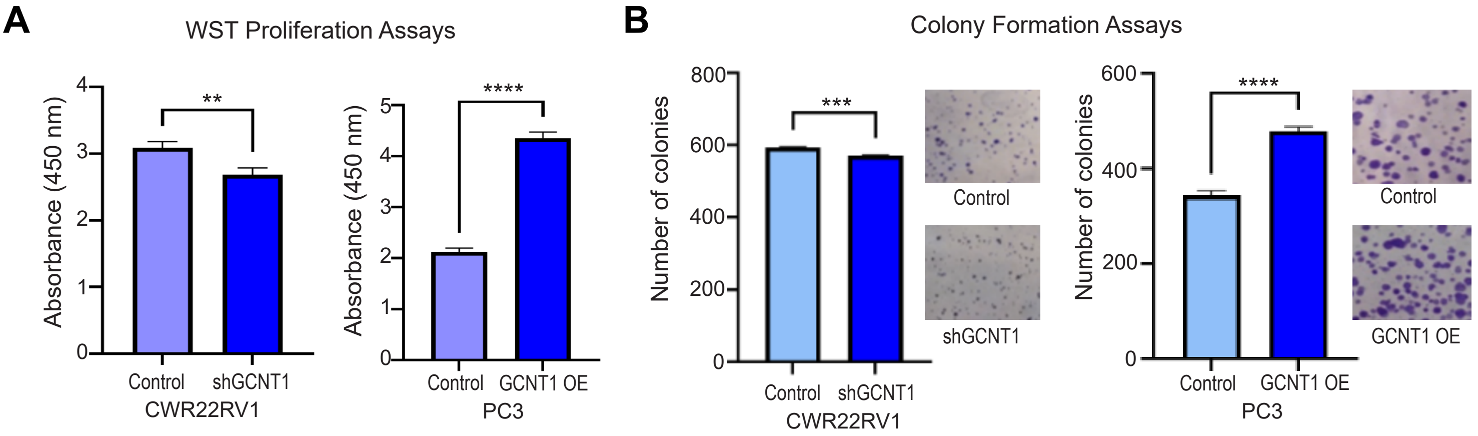

Supplementary Figure 4

Intra-cardiac injection study suggests upregulation of GCNT1 reduces the seeding potential of PC3 prostate cancer cells

A

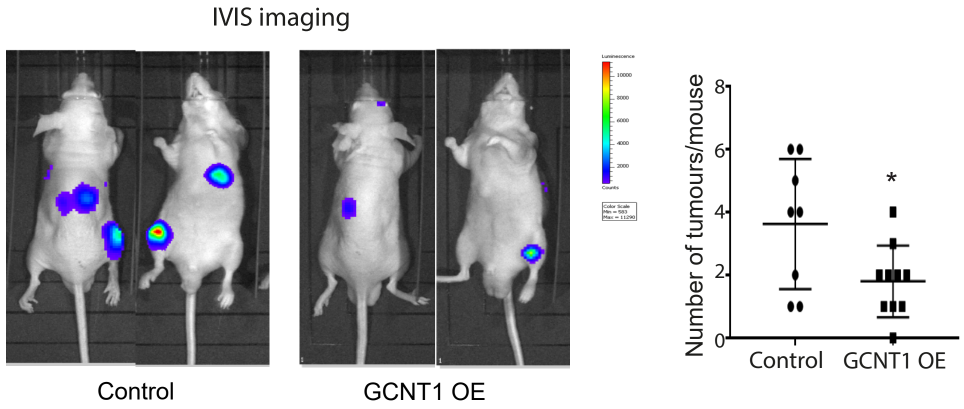

TableS1: List of differentially expressed genes in PC3 cells with upregulation of GCNT1

ENTREZID	SYMBOL	logFC	AveExpr	t	P.Value	adj.P.Val	B
55959	SULF2	-3.3604	7.09012	-53.78	1.85E-16	2.35E-12	28.2813
4642	MYO1D	-7.2701	4.86978	-48.3787	7.12E-16	4.44E-12	25.06
4747	NEFL	-2.4423	6.92021	-46.9298	1.05E-15	4.44E-12	26.5863
3339	HSPG2	-3.1981	7.06358	-44.2787	2.20E-15	6.98E-12	25.8488
6482	ST3GAL1	-2.0432	8.44341	-41.2402	5.43E-15	1.18E-11	24.8885
5950	RBP4	-4.5656	4.26406	-40.88	6.07E-15	1.18E-11	24.1343
55304	SPTLC3	-4.4744	4.95892	-40.663	6.49E-15	1.18E-11	24.4694
3914	LAMB3	2.85073	6.42342	40.06294	7.84E-15	1.19E-11	24.5785
2321	FLT1	5.50806	4.16823	39.83073	8.44E-15	1.19E-11	23.3875
290	ANPEP	-2.0348	7.84072	-38.4256	1.33E-14	1.69E-11	23.9863
6879	TAF7	2.74067	5.11857	37.59776	1.76E-14	2.03E-11	23.7327
222663	SCUBE3	-5.5387	3.58309	-36.3444	2.70E-14	2.86E-11	22.076
1293	COL6A3	-2.8264	5.33788	-35.3196	3.88E-14	3.75E-11	22.9765
26022	TMEM98	-10.879	0.72913	-35.1444	4.13E-14	3.75E-11	18.0291
140828	LINC00261	-2.0296	7.58539	-34.373	5.47E-14	4.63E-11	22.5368
3625	INHBB	-1.8283	8.05289	-33.4349	7.77E-14	6.16E-11	22.1432
399979	SNX19	2.75712	4.76552	33.27693	8.25E-14	6.16E-11	22.1993
3691	ITGB4	-1.668	8.33258	-32.0942	1.30E-13	9.20E-11	21.5853
645369	TMEM200C	-1.9479	5.70609	-31.7479	1.50E-13	1.00E-10	21.6103
1809	DPYSL3	-8.3716	2.62337	-31.3509	1.75E-13	1.11E-10	18.3402
23708	GSPT2	-10.051	0.31482	-31.1833	1.88E-13	1.14E-10	17.3823
3936	LCP1	-6.9493	2.59778	-30.4804	2.50E-13	1.45E-10	18.9304
3488	IGFBP5	1.48616	7.65357	30.05967	2.98E-13	1.55E-10	20.7589
157638	LRATD2	-6.9099	3.12461	-29.8885	3.21E-13	1.55E-10	19.3122
6778	STAT6	4.98562	3.17458	29.85973	3.25E-13	1.55E-10	20.004
50486	G0S2	3.73146	5.6055	30.38227	3.27E-13	1.55E-10	20.8627
56172	ANKH	-2.9576	4.30902	-29.8216	3.30E-13	1.55E-10	20.8099
6649	SOD3	-2.4016	6.33399	-29.4936	3.79E-13	1.72E-10	20.6198
134111	UBE2QL1	-1.9866	5.8936	-29.3402	4.05E-13	1.72E-10	20.5773
1469	CST1	-2.7694	5.27032	-29.3387	4.05E-13	1.72E-10	20.6303
55783	CMTR2	2.9968	4.20792	29.05383	4.58E-13	1.88E-10	20.4692
10550	ARL6IP5	1.58726	6.58434	28.51631	5.80E-13	2.30E-10	20.1437
9715	FAM131B	-1.7894	6.48943	-28.3856	6.15E-13	2.37E-10	20.0917
54704	PDP1	1.66628	6.37122	28.23612	6.57E-13	2.46E-10	20.0339
133418	EMB	-9.2551	-0.08233	-27.8585	7.79E-13	2.83E-10	16.6824
25791	NGEF	-1.5479	6.5122	-27.7037	8.35E-13	2.93E-10	19.7654
389206	BEND4	-8.7584	-0.33175	-27.6574	8.53E-13	2.93E-10	16.6119
55258	THNSL2	-8.4266	-0.49672	-27.5891	8.80E-13	2.94E-10	16.5744
22948	CCT5	-1.2196	8.88625	-27.4738	9.28E-13	3.02E-10	19.488
8638	OASL	2.08567	5.10578	27.263	1.02E-12	3.25E-10	19.6908
4651	MYO10	-1.3805	7.31544	-27.0734	1.12E-12	3.46E-10	19.3944
6563	SLC14A1	-2.0563	4.84483	-26.9384	1.19E-12	3.60E-10	19.549
8613	PLPP3	-1.5354	6.42282	-26.6862	1.34E-12	3.95E-10	19.2812
5865	RAB3B	2.43458	4.57933	26.09452	1.77E-12	5.12E-10	19.1635
8673	VAMP8	2.22756	4.45903	25.89515	1.95E-12	5.52E-10	19.068
1756	DMD	-3.8793	2.95222	-25.6383	2.21E-12	6.12E-10	18.5966
6781	STC1	-5.5629	2.69034	-25.5678	2.29E-12	6.19E-10	18.029

100505817	LINC02582	-1.2787	7.38939	-25.5266	2.34E-12	6.19E-10	18.6083
134147	CMBL	-1.2892	6.68107	-25.3681	2.53E-12	6.56E-10	18.5856
5834	PYGB	-1.0176	9.64489	-25.2915	2.63E-12	6.63E-10	18.3451
131583	FAM43A	-2.7457	4.48351	-25.2416	2.69E-12	6.63E-10	18.7474
3383	ICAM1	1.9237	5.99934	25.19192	2.76E-12	6.63E-10	18.5806
134265	AFAP1L1	-1.7761	5.29752	-25.1538	2.81E-12	6.63E-10	18.6232
3428	IFI16	2.98176	3.79628	25.1503	2.82E-12	6.63E-10	18.6686
5454	POU3F2	-3.8906	2.83531	-25.0309	2.99E-12	6.82E-10	18.2818
104	ADARB1	1.51045	5.78391	24.96159	3.10E-12	6.82E-10	18.472
387914	SHISA2	-7.8249	-0.79739	-24.9436	3.13E-12	6.82E-10	15.8539
6389	SDHA	-1.2849	8.05809	-24.9253	3.16E-12	6.82E-10	18.2428
6641	SNTB1	-2.5681	4.2	-24.9181	3.17E-12	6.82E-10	18.5884
1824	DSC2	-1.9638	5.87867	-24.7818	3.39E-12	7.18E-10	18.3743
1004	CDH6	-2.6894	4.3543	-24.5879	3.75E-12	7.78E-10	18.4194
4842	NOS1	-8.4094	-0.50646	-24.5607	3.80E-12	7.78E-10	15.7945
4756	NEO1	3.84278	3.80321	24.34331	4.25E-12	8.56E-10	18.2257
7474	WNT5A	-1.7543	5.06216	-24.1288	4.74E-12	9.26E-10	18.1066
2697	GJA1	-1.6958	5.75688	-24.1175	4.77E-12	9.26E-10	18.025
4163	MCC	-4.3386	3.09188	-24.1007	4.81E-12	9.26E-10	17.8883
2049	EPHB3	-2.2161	4.91907	-24.001	5.07E-12	9.61E-10	18.0644
1292	COL6A2	-1.3971	7.99031	-23.9465	5.22E-12	9.75E-10	17.7167
54491	OTULINL	-1.6743	5.74157	-23.8601	5.46E-12	1.00E-09	17.8856
687	KLF9	-4.271	2.76707	-23.7337	5.83E-12	1.06E-09	17.6057
114569	MAL2	-1.1524	8.92959	-23.686	5.98E-12	1.07E-09	17.5108
200407	CREG2	-1.6934	5.01728	-23.6006	6.26E-12	1.10E-09	17.8228
8905	AP1S2	-1.3467	6.23754	-23.5556	6.41E-12	1.12E-09	17.6545
3037	HAS2	-2.4692	3.79052	-23.4756	6.69E-12	1.15E-09	17.8412
5327	PLAT	1.03504	10.4631	23.44211	6.81E-12	1.15E-09	17.2931
23136	EPB41L3	8.85962	2.04905	23.30524	7.33E-12	1.23E-09	15.4916
63895	PIEZO2	-7.2238	-1.09826	-23.2286	7.64E-12	1.26E-09	15.278
1917	EEF1A2	-1.3644	8.37488	-23.1949	7.78E-12	1.27E-09	17.2667
114800	CCDC85A	-7.2503	-1.08503	-23.1072	8.16E-12	1.31E-09	15.2434
9173	IL1RL1	-2.4143	3.95972	-23.0294	8.51E-12	1.35E-09	17.6028
57214	CEMIP	-9.1758	0.40558	-22.9245	9.01E-12	1.41E-09	15.3432
23199	GSE1	1.19619	6.46369	22.84219	9.42E-12	1.46E-09	17.2256
1058	CENPA	-1.6435	4.73753	-22.7905	9.69E-12	1.48E-09	17.3996
9435	CHST2	-2.9197	3.51944	-22.7637	9.84E-12	1.49E-09	17.4394
55841	WWC3	7.51302	1.3768	22.6929	1.02E-11	1.53E-09	15.2713
257019	FRMD3	-7.1306	-1.14498	-22.5368	1.12E-11	1.63E-09	15.0428
2108	ETFA	1.38796	5.93679	22.51983	1.13E-11	1.63E-09	17.103
154	ADRB2	-1.1807	6.38508	-22.5141	1.13E-11	1.63E-09	17.04
65980	BRD9	-1.3011	5.70763	-22.4695	1.16E-11	1.63E-09	17.0942
55784	MCTP2	-8.0464	-0.68907	-22.4474	1.17E-11	1.63E-09	15.1086
375057	STUM	-8.4632	-0.48021	-22.4327	1.18E-11	1.63E-09	15.1315
5029	P2RY2	1.30384	5.7328	22.43164	1.18E-11	1.63E-09	17.0736
3486	IGFBP3	-1.4171	7.54382	-22.3916	1.21E-11	1.65E-09	16.8619
10076	PTPRU	-3.5358	3.5508	-22.2418	1.31E-11	1.78E-09	17.137
9537	TP53I11	-5.5845	1.96449	-22.1536	1.38E-11	1.84E-09	16.1625
9119	KRT75	-5.9166	2.76133	-22.1396	1.39E-11	1.84E-09	16.5266
586	BCAT1	-7.1578	-1.13091	-22.1269	1.40E-11	1.84E-09	14.9091

9787	DLGAP5	-1.0511	7.81324	-22.0992	1.42E-11	1.85E-09	16.6619
6695	SPOCK1	-6.651	0.93698	-22.0231	1.49E-11	1.91E-09	15.0132
56101	PCDHGB5	4.47677	2.25163	22.00029	1.51E-11	1.91E-09	16.4891
100505806	SNHG18	-7.0465	-1.18826	-21.9185	1.58E-11	1.99E-09	14.8216
6549	SLC9A2	-8.1157	-0.6539	-21.8376	1.65E-11	2.05E-09	14.9024
1291	COL6A1	-1.0101	9.32972	-21.8256	1.66E-11	2.05E-09	16.4001
4680	CEACAM6	-9.0851	0.60638	-21.733	1.76E-11	2.13E-09	14.9341
114897	C1QTNF1	2.15077	4.58688	21.72748	1.76E-11	2.13E-09	16.8219
93664	CADPS2	-2.3057	4.84569	-21.656	1.83E-11	2.18E-09	16.7495
5155	PDGFB	2.56961	4.28076	21.65406	1.84E-11	2.18E-09	16.8185
4923	NTSR1	-1.1732	7.23214	-21.5792	1.92E-11	2.26E-09	16.3965
4082	MARCKS	-1.0487	6.97752	-21.3711	2.16E-11	2.52E-09	16.2905
4735	SEPTIN2	-0.8489	8.67867	-21.3123	2.24E-11	2.59E-09	16.1216
221	ALDH3B1	1.51983	4.95453	21.2529	2.32E-11	2.65E-09	16.4723
57568	SIPA1L2	-1.9269	4.59331	-21.063	2.59E-11	2.94E-09	16.408
23414	ZFPM2	-7.4953	1.66723	-21.0021	2.69E-11	3.02E-09	14.802
1397	CRIP2	5.34897	2.18474	20.9725	2.74E-11	3.05E-09	15.7654
57491	AHRR	-2.3352	3.39597	-20.8683	2.91E-11	3.22E-09	16.3773
4052	LTBP1	-1.1607	7.92949	-20.7682	3.09E-11	3.36E-09	15.8316
9240	PNMA1	-0.9803	7.499	-20.7649	3.10E-11	3.36E-09	15.8621
9319	TRIP13	-1.1191	6.35089	-20.7317	3.16E-11	3.39E-09	15.957
25803	SPDEF	-3.1311	2.99608	-20.7227	3.18E-11	3.39E-09	16.2402
1289	COL5A1	-8.0436	0.61387	-20.6737	3.27E-11	3.46E-09	14.521
871	SERPINH1	0.99276	7.25291	20.60928	3.40E-11	3.57E-09	15.7874
3783	KCNN4	-1.0156	6.70123	-20.5749	3.47E-11	3.61E-09	15.8169
8801	SUCLG2	1.30765	5.57631	20.56289	3.50E-11	3.61E-09	15.9549
6567	SLC16A2	-1.724	4.12166	-20.4157	3.83E-11	3.91E-09	16.0546
25849	PARM1	-6.7119	1.04653	-20.3965	3.87E-11	3.91E-09	14.4189
2631	NIPSNAP2	-1.1447	5.92469	-20.3932	3.88E-11	3.91E-09	15.7933
11044	TENT4A	-1.1787	6.13698	-20.2918	4.13E-11	4.13E-09	15.7021
107	ADCY1	-7.4977	0.09412	-20.2308	4.28E-11	4.20E-09	14.3157
6617	SNAPC1	0.89128	8.02309	20.22049	4.31E-11	4.20E-09	15.4704
664	BNIP3	-0.8719	7.81009	-20.2167	4.32E-11	4.20E-09	15.4824
2335	FN1	-1.2481	9.65765	-20.2134	4.33E-11	4.20E-09	15.3646
55355	HJURP	-1.1672	6.41682	-20.1702	4.45E-11	4.28E-09	15.5891
6662	SOX9	-1.0661	7.66036	-20.1298	4.56E-11	4.35E-09	15.4392
5577	PRKAR2B	-3.9944	2.32517	-20.0853	4.69E-11	4.44E-09	15.6481
7477	WNT7B	-2.7975	3.38249	-20.0592	4.76E-11	4.48E-09	15.8868
4884	NPTX1	-3.5003	2.69254	-19.9874	4.98E-11	4.63E-09	15.7401
5165	PDK3	-1.7015	5.49673	-19.973	5.02E-11	4.63E-09	15.5918
22974	TPX2	-0.8648	8.51686	-19.9707	5.03E-11	4.63E-09	15.2708
230	ALDOC	-1.252	6.2767	-19.9132	5.21E-11	4.77E-09	15.439
10016	PDCD6	-1.0234	7.27449	-19.81	5.56E-11	5.02E-09	15.2612
994	CDC25B	-1.2203	7.69557	-19.8079	5.57E-11	5.02E-09	15.2253
10403	NDC80	-0.9939	6.70301	-19.7344	5.83E-11	5.22E-09	15.2673
154822	LINC00689	-7.3329	-1.04596	-19.691	5.99E-11	5.33E-09	14.0063
126755	LRRC38	-7.263	-1.07829	-19.6589	6.12E-11	5.40E-09	13.9862
145407	ARMH4	-4.6839	3.48429	-19.6855	6.43E-11	5.53E-09	15.5463
10397	NDRG1	-0.914	9.35084	-19.5736	6.46E-11	5.53E-09	14.9546
11336	EXOC3	-1.4704	5.41362	-19.5713	6.47E-11	5.53E-09	15.3308

4907	NT5E	-0.9844	7.14311	-19.5691	6.47E-11	5.53E-09	15.112
81618	ITM2C	-0.8949	7.83679	-19.5664	6.49E-11	5.53E-09	15.0488
2824	GPM6B	-6.5053	-1.45896	-19.4739	6.88E-11	5.83E-09	13.8085
84246	MED10	-1.0881	6.04204	-19.4383	7.04E-11	5.92E-09	15.1485
3939	LDHA	-0.6551	11.6154	-19.4232	7.11E-11	5.94E-09	14.735
1131	CHRM3	-2.0053	4.59781	-19.3247	7.57E-11	6.25E-09	15.3011
51129	ANGPTL4	-1.9541	4.01789	-19.318	7.60E-11	6.25E-09	15.3681
285203	EOGT	1.96639	3.91017	19.30844	7.65E-11	6.25E-09	15.379
10570	DPYSL4	-1.9864	4.37278	-19.3029	7.67E-11	6.25E-09	15.3158
5789	PTPRD	-2.8945	2.52892	-19.2825	7.78E-11	6.29E-09	15.3314
2201	FBN2	-1.0335	9.42193	-19.1833	8.29E-11	6.62E-09	14.6847
3624	INHBA	-1.5467	4.62684	-19.1832	8.29E-11	6.62E-09	15.1852
5328	PLAU	1.09458	9.2959	19.12029	8.63E-11	6.80E-09	14.6498
10468	FST	-0.9452	8.03396	-19.1193	8.64E-11	6.80E-09	14.7293
4139	MARK1	-7.1831	-0.59038	-19.1146	8.67E-11	6.80E-09	13.7908
3800	KIF5C	-4.0357	1.59808	-19.0809	8.86E-11	6.90E-09	14.8162
4599	MX1	3.60046	3.57873	19.04959	9.04E-11	6.99E-09	15.2486
4070	TACSTD2	6.44479	-1.39016	19.04187	9.08E-11	6.99E-09	13.645
1512	CTSH	-1.2519	5.20559	-18.9905	9.39E-11	7.19E-09	14.9616
10112	KIF20A	-1.0453	6.30542	-18.9698	9.52E-11	7.22E-09	14.7946
54463	RETREG1	-4.1593	1.94588	-18.9548	9.61E-11	7.22E-09	14.8647
3398	ID2	-1.4319	4.60297	-18.9489	9.65E-11	7.22E-09	15.0262
54443	ANLN	-0.9687	8.16747	-18.9481	9.66E-11	7.22E-09	14.601
9644	SH3PXD2A	-3.9447	2.69393	-18.9376	9.72E-11	7.22E-09	15.0797
10610	ST6GALNAC2	-1.4358	4.76715	-18.8785	1.01E-10	7.47E-09	14.9545
284716	RIMKLA	-1.1625	6.02443	-18.8659	1.02E-10	7.48E-09	14.7607
79083	MLPH	-1.0288	6.9292	-18.8431	1.03E-10	7.55E-09	14.6363
5420	PODXL	1.38962	5.40583	18.79359	1.07E-10	7.76E-09	14.8067
7837	PXDN	-0.8697	8.22906	-18.7386	1.11E-10	8.00E-09	14.4495
9388	LIPG	-8.0786	0.10243	-18.716	1.12E-10	8.07E-09	13.668
90952	ESAM	-2.1539	4.25235	-18.6517	1.17E-10	8.38E-09	14.8994
9922	IQSEC1	-0.9478	7.28459	-18.6423	1.18E-10	8.38E-09	14.4593
10130	PDIA6	-0.7794	8.91001	-18.635	1.19E-10	8.38E-09	14.3312
9976	CLEC2B	-5.4306	1.14139	-18.6072	1.21E-10	8.49E-09	14.0349
85460	ZNF518B	5.65623	1.83462	18.51335	1.29E-10	8.98E-09	14.2579
7138	TNNT1	-1.0549	6.70937	-18.4654	1.33E-10	9.14E-09	14.3939
133957	CCDC127	-1.2741	5.24946	-18.4651	1.33E-10	9.14E-09	14.5908
154661	RUNDC3B	3.91661	2.1213	18.46273	1.33E-10	9.14E-09	14.636
9397	NMT2	1.11619	5.77733	18.43374	1.36E-10	9.27E-09	14.4941
7421	VDR	3.70305	2.29603	18.4123	1.38E-10	9.36E-09	14.6744
11164	NUDT5	1.18428	5.6545	18.38602	1.40E-10	9.47E-09	14.4793
84620	ST6GAL2	-6.2713	-1.57608	-18.341	1.44E-10	9.71E-09	13.2746
157506	RDH10	-1.115	5.87218	-18.3296	1.46E-10	9.73E-09	14.4027
9037	SEMA5A	-7.4593	-0.45346	-18.2929	1.49E-10	9.93E-09	13.4307
9289	ADGRG1	-1.6319	4.54279	-18.2688	1.52E-10	1.00E-08	14.5702
5054	SERPINE1	1.55501	9.12812	20.33973	1.52E-10	1.00E-08	14.2245
64065	PERP	-0.8575	7.04932	-18.2219	1.57E-10	1.03E-08	14.1815
55691	FRMD4A	2.69086	3.2786	18.21365	1.57E-10	1.03E-08	14.6982
4537	ND3	0.88174	8.25187	18.16262	1.63E-10	1.06E-08	14.0378
6790	AURKA	-0.8957	7.1048	-18.0708	1.74E-10	1.12E-08	14.0671

6167	RPL37	-0.8181	9.23688	-18.0614	1.75E-10	1.12E-08	13.8995
10628	TXNIP	-0.9486	6.92281	-18.0449	1.77E-10	1.12E-08	14.0675
26207	PITPNC1	-1.0581	5.98758	-18.0435	1.77E-10	1.12E-08	14.1804
284611	FAM102B	-1.3876	5.13482	-17.977	1.85E-10	1.17E-08	14.2633
10795	ZNF268	3.55484	1.87518	17.9583	1.87E-10	1.17E-08	14.2995
2203	FBP1	2.53425	3.44804	17.95423	1.88E-10	1.17E-08	14.514
11082	ESM1	-1.2119	5.38176	-17.9527	1.88E-10	1.17E-08	14.2033
642273	FAM110C	-6.0523	0.71623	-17.9347	1.91E-10	1.18E-08	13.2916
10299	MARCHF6	-1.0405	7.28458	-17.9274	1.91E-10	1.18E-08	13.9468
4856	CCN3	-1.4398	4.94646	-17.9164	1.93E-10	1.18E-08	14.2501
1265	CNN2	-1.6769	4.71875	-17.9152	1.93E-10	1.18E-08	14.2928
387496	RASL11A	-1.3562	5.30601	-17.8263	2.05E-10	1.24E-08	14.1267
3069	HDLBP	-0.6952	9.66719	-17.8216	2.06E-10	1.24E-08	13.6987
8877	SPHK1	3.24133	3.79492	17.82094	2.06E-10	1.24E-08	14.4173
80020	FOXRED2	-1.5106	5.21464	-17.8104	2.08E-10	1.24E-08	14.1342
441518	RTL8B	-6.0792	2.20688	-17.763	2.15E-10	1.28E-08	13.9488
114088	TRIM9	-1.5203	4.86936	-17.7553	2.16E-10	1.28E-08	14.1481
56925	LXN	-1.3129	4.55872	-17.7531	2.16E-10	1.28E-08	14.1881
9039	UBA3	1.20534	5.65268	17.6554	2.31E-10	1.36E-08	13.9515
81610	FAM83D	-0.9906	6.66828	-17.6427	2.33E-10	1.37E-08	13.8004
100133941	CD24	-3.0012	2.35474	-17.587	2.43E-10	1.41E-08	14.2184
3672	ITGA1	-4.5628	2.49456	-17.5348	2.52E-10	1.45E-08	14.1
10497	UNC13B	-1.2356	6.2939	-17.5309	2.52E-10	1.45E-08	13.767
23462	HEY1	-1.1727	5.62146	-17.5238	2.54E-10	1.45E-08	13.8525
9123	SLC16A3	-0.9487	8.19793	-17.5203	2.54E-10	1.45E-08	13.5678
4753	NELL2	-2.4054	3.01569	-17.4854	2.60E-10	1.48E-08	14.1973
224	ALDH3A2	-1.0623	7.19794	-17.4809	2.61E-10	1.48E-08	13.6249
8714	ABCC3	-0.9231	6.65497	-17.468	2.64E-10	1.49E-08	13.6708
2495	FTH1	-0.7213	12.5122	-17.4533	2.66E-10	1.49E-08	13.2815
1508	CTSB	0.83787	8.73008	17.45112	2.67E-10	1.49E-08	13.4798
4939	OAS2	1.69759	6.00769	17.56463	2.68E-10	1.49E-08	13.7711
164284	APCDD1L	-1.7683	3.85209	-17.4349	2.70E-10	1.50E-08	14.0775
5738	PTGFRN	-3.5448	1.90486	-17.367	2.83E-10	1.56E-08	13.9542
23555	TSPAN15	1.13938	5.86964	17.34313	2.88E-10	1.58E-08	13.6863
5500	PPP1CB	-1.0106	7.65462	-17.3177	2.93E-10	1.61E-08	13.4587
6916	TBXAS1	-1.4209	4.89649	-17.2986	2.97E-10	1.62E-08	13.8032
9770	RASSF2	-1.6144	4.39083	-17.2611	3.05E-10	1.66E-08	13.8635
79668	PARP8	-6.2859	0.89384	-17.2164	3.15E-10	1.70E-08	12.9556
79625	NDNF	-3.9305	3.07205	-17.1601	3.28E-10	1.77E-08	13.9584
85453	TSPYL5	1.16711	5.55479	17.15074	3.30E-10	1.77E-08	13.5874
27350	APOBEC3C	-0.9419	6.97895	-17.12	3.38E-10	1.80E-08	13.3724
22998	LIMCH1	-3.4128	2.20267	-17.1138	3.39E-10	1.80E-08	13.8546
864	RUNX3	-6.8326	-0.76535	-17.1033	3.42E-10	1.81E-08	12.7923
729678	LINC00847	1.78804	3.86626	17.10043	3.43E-10	1.81E-08	13.8371
23467	NPTXR	-1.5527	5.10156	-17.0664	3.51E-10	1.84E-08	13.5996
2665	GDI2	0.79347	7.16157	17.05021	3.55E-10	1.86E-08	13.3008
57476	GRAMD1B	0.88313	7.8189	17.04094	3.58E-10	1.86E-08	13.2342
10687	PNMA2	-6.6402	-0.33392	-16.9987	3.69E-10	1.91E-08	12.7599
8228	PNPLA4	5.31813	0.96829	16.98909	3.71E-10	1.91E-08	13.0433
8853	ASAP2	-0.9527	6.95635	-16.9861	3.72E-10	1.91E-08	13.2723

54888	NSUN2	-0.8306	7.32805	-16.9816	3.73E-10	1.91E-08	13.2297
51203	NUSAP1	-0.7568	7.95992	-16.9523	3.81E-10	1.94E-08	13.1524
4102	MAGEA3	-6.1502	1.61296	-16.9096	3.93E-10	2.00E-08	13.1748
23560	GTPBP4	0.99535	5.99637	16.87633	4.03E-10	2.04E-08	13.3097
79674	VEPH1	1.34347	5.06088	16.87149	4.04E-10	2.04E-08	13.457
10318	TNIP1	-0.8003	7.4583	-16.8616	4.07E-10	2.04E-08	13.1244
39	ACAT2	-0.9684	5.93979	-16.8576	4.08E-10	2.04E-08	13.2983
27004	TCL6	-5.6229	-1.89848	-16.8198	4.20E-10	2.09E-08	12.424
122953	JDP2	-5.6725	-0.043	-16.8168	4.21E-10	2.09E-08	12.6499
4519	CYTB	0.91334	10.5442	16.79841	4.26E-10	2.11E-08	12.8754
55244	SLC47A1	-6.7358	-0.81363	-16.7884	4.30E-10	2.11E-08	12.6168
55086	RADX	-4.3329	1.84586	-16.7467	4.43E-10	2.17E-08	13.4496
7153	TOP2A	-0.7329	8.66675	-16.7347	4.47E-10	2.18E-08	12.933
1630	DCC	-5.736	-1.8429	-16.7264	4.50E-10	2.18E-08	12.3949
55771	PRR11	-0.7699	8.06077	-16.7241	4.50E-10	2.18E-08	12.9674
5774	PTPN3	1.68321	4.30736	16.68797	4.62E-10	2.23E-08	13.4545
54855	TENT5C	-2.6548	3.23616	-16.6802	4.65E-10	2.24E-08	13.6107
390963	ZNF818P	-2.0958	3.15529	-16.6537	4.74E-10	2.27E-08	13.584
54898	ELOVL2	-2.2727	2.68829	-16.6248	4.84E-10	2.31E-08	13.5816
100859930	HEIH	1.86379	3.51879	16.62326	4.85E-10	2.31E-08	13.5259
5021	OXTR	-1.381	4.70757	-16.6033	4.92E-10	2.33E-08	13.3028
6507	SLC1A3	-5.9828	0.1114	-16.5941	4.96E-10	2.34E-08	12.5468
85352	SHISAL1	-6.2102	-1.07653	-16.5357	5.17E-10	2.43E-08	12.4301
5757	PTMA	-0.7276	9.70279	-16.4833	5.38E-10	2.52E-08	12.6718
6721	SREBF2	-0.7812	7.07024	-16.4527	5.50E-10	2.57E-08	12.8416
1767	DNAH5	-1.6798	3.93518	-16.4342	5.58E-10	2.60E-08	13.3104
2637	GBX2	-3.5061	2.20444	-16.392	5.76E-10	2.67E-08	13.3504
1611	DAP	-0.7518	9.46771	-16.3605	5.90E-10	2.71E-08	12.5874
4609	MYC	1.03993	7.0368	16.35845	5.90E-10	2.71E-08	12.7764
8763	CD164	-0.7728	8.62663	-16.3522	5.93E-10	2.71E-08	12.6332
5552	SRGN	0.93843	7.64559	16.33072	6.03E-10	2.74E-08	12.6931
3838	KPNA2	-0.737	8.74413	-16.3173	6.09E-10	2.75E-08	12.5973
4851	NOTCH1	1.23278	4.79834	16.31313	6.11E-10	2.75E-08	13.0621
5915	RARB	-5.7507	-1.83288	-16.3116	6.12E-10	2.75E-08	12.177
3491	CCN1	1.99654	7.56116	18.71803	6.13E-10	2.75E-08	12.9169
57415	C3orf14	3.01632	1.90277	16.29628	6.19E-10	2.76E-08	13.252
1501	CTNND2	-5.4803	0.35084	-16.2942	6.20E-10	2.76E-08	12.3778
27242	TNFRSF21	-0.8646	8.11941	-16.2788	6.27E-10	2.78E-08	12.6108
9590	AKAP12	-1.0074	9.07096	-16.278	6.27E-10	2.78E-08	12.5462
55789	DEPDC1B	-1.8043	3.65952	-16.2719	6.30E-10	2.78E-08	13.2298
5230	PGK1	-0.5897	11.1013	-16.2633	6.34E-10	2.78E-08	12.4212
7980	TFPI2	0.90113	7.38688	16.26199	6.35E-10	2.78E-08	12.6617
10409	BASP1	-1.1537	6.28788	-16.2226	6.54E-10	2.85E-08	12.755
84969	TOX2	-7.6243	0.16039	-16.2221	6.54E-10	2.85E-08	12.3715
4338	MOCS2	-1.0781	5.26144	-16.2074	6.62E-10	2.87E-08	12.8915
1903	S1PR3	-1.3199	4.8842	-16.1976	6.66E-10	2.88E-08	12.9527
63027	SLC22A23	-0.9894	6.09425	-16.1806	6.75E-10	2.91E-08	12.7445
25941	TPGS2	-1.0369	6.15423	-16.1658	6.83E-10	2.93E-08	12.7253
3575	IL7R	1.18802	5.76219	16.15975	6.86E-10	2.93E-08	12.7843
4093	SMAD9	-2.6917	2.16479	-16.1228	7.05E-10	3.01E-08	13.184

10529	NEBL	-1.7967	5.98233	-17.0552	7.39E-10	3.14E-08	12.7782
11004	KIF2C	-1.0136	6.59925	-16.0572	7.41E-10	3.14E-08	12.5799
207107	SFTA1P	5.87764	1.08875	16.02884	7.58E-10	3.20E-08	12.4138
9780	PIEZO1	0.74261	7.79967	16.02053	7.62E-10	3.21E-08	12.4276
54101	RIPK4	-1.4056	3.94359	-15.9263	8.19E-10	3.43E-08	12.8989
51339	DACT1	-3.6735	1.76244	-15.9226	8.22E-10	3.43E-08	12.9279
24145	PANX1	-0.91	6.05716	-15.8705	8.55E-10	3.56E-08	12.4969
6241	RRM2	-0.6976	8.19306	-15.8282	8.84E-10	3.66E-08	12.2383
286133	SCARA5	-1.6508	5.1059	-15.8266	8.85E-10	3.66E-08	12.6288
891	CCNB1	-1.0998	6.95043	-15.8153	8.92E-10	3.68E-08	12.3438
114784	CSMD2	-6.4064	-0.97731	-15.7968	9.05E-10	3.72E-08	12.0302
53834	FGFRL1	1.50518	4.11227	15.74199	9.45E-10	3.87E-08	12.7343
64782	AEN	1.05759	5.27623	15.72177	9.60E-10	3.92E-08	12.5006
6678	SPARC	-1.9088	7.77904	-18.0012	9.65E-10	3.93E-08	12.4077
58526	MID1IP1	1.32501	4.04949	15.68639	9.86E-10	4.00E-08	12.6923
51760	SYT17	-2.0602	3.23171	-15.6522	1.01E-09	4.10E-08	12.8036
10103	TSPAN1	1.29937	4.6359	15.64156	1.02E-09	4.12E-08	12.5519
4675	NAP1L3	1.50558	4.61023	15.62445	1.04E-09	4.16E-08	12.5505
3161	HMMR	-0.8912	6.64088	-15.6202	1.04E-09	4.16E-08	12.2147
112942	CFAP36	-0.9978	5.2853	-15.609	1.05E-09	4.19E-08	12.3995
83959	SLC4A11	-1.376	4.91927	-15.5878	1.07E-09	4.24E-08	12.4542
3437	IFIT3	1.19689	5.16874	15.58447	1.07E-09	4.24E-08	12.4095
3745	KCNB1	-4.3356	1.05275	-15.5766	1.07E-09	4.25E-08	12.4184
23379	ICE1	-1.0738	6.76779	-15.5117	1.13E-09	4.45E-08	12.112
115286	SLC25A26	1.82706	3.5398	15.51119	1.13E-09	4.45E-08	12.651
2781	GNAZ	-1.4491	4.49182	-15.4953	1.15E-09	4.49E-08	12.4548
83463	MXD3	-1.558	3.97007	-15.4719	1.17E-09	4.56E-08	12.5323
945	CD33	-2.8673	2.36434	-15.444	1.19E-09	4.65E-08	12.6822
8553	BHLHE40	-0.8333	8.18797	-15.436	1.20E-09	4.66E-08	11.9127
1152	CKB	-1.6676	3.70615	-15.4103	1.22E-09	4.73E-08	12.5293
3320	HSP90AA1	-0.601	11.219	-15.4086	1.23E-09	4.73E-08	11.71
3294	HSD17B2	-0.8702	6.03232	-15.4056	1.23E-09	4.73E-08	12.1143
2273	FHL1	0.93959	8.42024	15.4025	1.23E-09	4.73E-08	11.8689
10256	CNKSR1	3.07575	2.27695	15.39602	1.24E-09	4.73E-08	12.6319
3835	KIF22	-0.8784	6.06792	-15.3938	1.24E-09	4.73E-08	12.0996
397	ARHGDIB	-4.2123	0.83532	-15.3878	1.25E-09	4.74E-08	12.2296
55635	DEPDC1	-1.0215	5.52972	-15.3849	1.25E-09	4.74E-08	12.1744
79589	RNF128	-6.4981	-0.93362	-15.3768	1.26E-09	4.75E-08	11.7855
100287072	LOC100287072	-5.2693	-2.07691	-15.3642	1.27E-09	4.79E-08	11.5596
5732	PTGER2	-2.2181	3.1524	-15.3194	1.32E-09	4.93E-08	12.5498
4858	NOVA2	-5.8542	-1.25468	-15.3185	1.32E-09	4.93E-08	11.6897
54908	SPDL1	-0.8862	6.16067	-15.3102	1.33E-09	4.93E-08	12.0164
10231	RCAN2	-5.8589	-1.78404	-15.3094	1.33E-09	4.93E-08	11.619
7277	TUBA4A	1.03089	6.13236	15.30834	1.33E-09	4.93E-08	12.0255
10531	PITRM1	0.7781	6.74219	15.24332	1.40E-09	5.14E-08	11.8871
65268	WNK2	-3.2348	1.85384	-15.2424	1.40E-09	5.14E-08	12.473
23596	OPN3	-1.0487	5.64644	-15.2417	1.40E-09	5.14E-08	12.0362
55713	ZNF334	-5.3424	-2.03783	-15.2377	1.40E-09	5.14E-08	11.4985
83666	PARP9	1.1445	5.89357	15.23492	1.41E-09	5.14E-08	12.0009
90161	HS6ST2	-5.4554	-1.98322	-15.2338	1.41E-09	5.14E-08	11.5142

51512	GTSE1	-1.0581	5.90736	-15.224	1.42E-09	5.17E-08	11.9826
8347	H2BC4	1.25593	4.50142	15.20621	1.44E-09	5.22E-08	12.2122
222537	HS3ST5	-2.3852	2.14789	-15.2031	1.44E-09	5.22E-08	12.4915
4837	NNMT	5.04257	1.2094	15.2015	1.45E-09	5.22E-08	12.0904
9915	ARNT2	-2.8315	1.82872	-15.1759	1.48E-09	5.31E-08	12.4351
55796	MBNL3	6.29074	-0.17381	15.16071	1.49E-09	5.36E-08	11.6973
6558	SLC12A2	-1.5927	6.93743	-16.7357	1.50E-09	5.37E-08	11.959
117581	TWIST2	-5.3407	-0.20803	-15.1408	1.52E-09	5.42E-08	11.6504
90639	COX19	1.1504	4.64513	15.13651	1.52E-09	5.42E-08	12.1239
10395	DLC1	1.53716	3.85448	15.10449	1.56E-09	5.55E-08	12.2546
10494	STK25	-0.7926	6.51719	-15.082	1.59E-09	5.62E-08	11.7743
6546	SLC8A1	-1.8375	3.87922	-15.0805	1.59E-09	5.62E-08	12.2354
3157	HMGCS1	-0.9958	5.8047	-15.0769	1.60E-09	5.62E-08	11.871
400954	EML6	-2.3782	2.85772	-15.0336	1.66E-09	5.81E-08	12.3459
71	ACTG1	-0.8166	11.2825	-15.0178	1.68E-09	5.87E-08	11.3727
51495	HACD3	0.6649	7.46729	15.00724	1.69E-09	5.88E-08	11.6069
51477	ISYNA1	-2.0039	3.77572	-15.0058	1.69E-09	5.88E-08	12.1966
2688	GH1	-5.2641	-2.07857	-15.0058	1.69E-09	5.88E-08	11.3455
79616	CCNJL	-0.819	6.28007	-14.997	1.71E-09	5.89E-08	11.732
6505	SLC1A1	-1.5136	3.96394	-14.9966	1.71E-09	5.89E-08	12.1352
9781	RNF144A	-0.8924	5.48534	-14.9927	1.71E-09	5.89E-08	11.8456
10971	YWHAQ	-0.6109	8.62767	-14.9749	1.74E-09	5.96E-08	11.4867
84740	AFAP1-AS1	-4.7291	1.11535	-14.9665	1.75E-09	5.98E-08	11.9694
112770	GLMP	0.85337	6.0474	14.93882	1.79E-09	6.10E-08	11.7179
699	BUB1	-0.8023	7.36924	-14.9206	1.81E-09	6.18E-08	11.5408
7791	ZYX	-0.8264	9.49096	-14.9173	1.82E-09	6.18E-08	11.3828
5362	PLXNA2	-2.3692	2.17591	-14.9016	1.84E-09	6.24E-08	12.2536
91351	DDX60L	1.38381	4.22918	14.89381	1.85E-09	6.26E-08	12.0024
29959	NRBP1	-0.6962	7.26819	-14.889	1.86E-09	6.26E-08	11.5223
23464	GCAT	-1.1756	4.54997	-14.8873	1.86E-09	6.26E-08	11.9228
146330	FBXL16	-2.3411	2.65413	-14.8538	1.92E-09	6.42E-08	12.2094
55504	TNFRSF19	-2.0056	3.87189	-14.8318	1.95E-09	6.52E-08	12.0348
3005	H1-0	-0.616	9.26008	-14.8129	1.98E-09	6.60E-08	11.3048
40	ASIC2	-5.5139	-1.95304	-14.8041	2.00E-09	6.64E-08	11.2614
11320	MGAT4A	-3.9609	1.1636	-14.7949	2.01E-09	6.67E-08	11.9471
30845	EHD3	-1.2122	4.70555	-14.7783	2.04E-09	6.74E-08	11.8022
6769	STAC	-6.1798	-0.56203	-14.7638	2.06E-09	6.81E-08	11.414
8506	CNTNAP1	-0.8805	5.65145	-14.7476	2.09E-09	6.87E-08	11.607
899	CCNF	-1.0138	5.38732	-14.746	2.09E-09	6.87E-08	11.6505
9060	PAPSS2	-0.7806	7.26852	-14.7359	2.11E-09	6.91E-08	11.3895
117177	RAB3IP	0.86725	5.80457	14.70753	2.16E-09	7.05E-08	11.5526
10605	PAIP1	-0.7747	6.49912	-14.7031	2.17E-09	7.06E-08	11.4476
10398	MYL9	1.91646	3.98324	14.69488	2.18E-09	7.09E-08	11.9032
1045	CDX2	-5.8014	-1.80686	-14.691	2.19E-09	7.10E-08	11.2309
8291	DYSF	1.46418	4.77763	14.67553	2.22E-09	7.17E-08	11.7164
8809	IL18R1	-3.5785	1.51993	-14.6698	2.23E-09	7.18E-08	11.9631
94031	HTRA3	-2.2653	2.25061	-14.6521	2.26E-09	7.27E-08	12.0526
10653	SPINT2	-0.8713	6.99302	-14.644	2.28E-09	7.30E-08	11.3387
51700	CYB5R2	-5.3572	-2.02996	-14.6239	2.31E-09	7.38E-08	11.125
143425	SYT9	-5.6313	0.71415	-14.6214	2.32E-09	7.38E-08	11.4348

55107	ANO1	-1.5768	3.48845	-14.6178	2.33E-09	7.38E-08	11.8952
57761	TRIB3	0.81775	6.4443	14.61689	2.33E-09	7.38E-08	11.3815
140862	ISM1	-5.2081	-2.1056	-14.6161	2.33E-09	7.38E-08	11.0959
23204	ARL6IP1	-0.6648	7.66275	-14.6107	2.34E-09	7.39E-08	11.2409
56133	PCDHB2	3.86812	2.08918	14.60847	2.34E-09	7.39E-08	11.972
6711	SPTBN1	-0.9141	8.61184	-14.602	2.36E-09	7.41E-08	11.1622
23741	EID1	-0.7555	7.28055	-14.5962	2.37E-09	7.43E-08	11.2648
5351	PLOD1	-0.7176	8.46744	-14.588	2.38E-09	7.46E-08	11.159
84722	PSRC1	-1.1855	4.79478	-14.5674	2.43E-09	7.57E-08	11.6012
11199	ANXA10	-1.5346	4.19531	-14.5549	2.45E-09	7.63E-08	11.7138
221687	RNF182	-0.8151	5.78724	-14.5485	2.46E-09	7.65E-08	11.4101
2023	ENO1	-0.6655	11.9122	-14.5336	2.50E-09	7.73E-08	10.9152
3791	KDR	-1.5428	6.49123	-15.6942	2.51E-09	7.76E-08	11.4323
26153	KIF26A	-6.0824	-0.36678	-14.5155	2.53E-09	7.81E-08	11.259
2200	FBN1	-1.5028	6.44833	-15.4489	2.56E-09	7.89E-08	11.392
4938	OAS1	1.7414	4.3792	14.46433	2.64E-09	8.11E-08	11.6199
4792	NFKBIA	2.07862	5.31684	15.95485	2.66E-09	8.14E-08	11.6086
388630	TRABD2B	-5.2559	-2.08213	-14.4364	2.71E-09	8.26E-08	10.9894
28951	TRIB2	-1.2981	5.18122	-14.4341	2.71E-09	8.26E-08	11.419
5583	PRKCH	0.70733	7.0204	14.40339	2.78E-09	8.46E-08	11.1223
100129434	LOC100129434	-5.0096	-2.20435	-14.3752	2.85E-09	8.64E-08	10.9101
9475	ROCK2	-1.11	6.99829	-14.3655	2.87E-09	8.69E-08	11.0938
55786	ZNF415	-1.797	3.02044	-14.3524	2.90E-09	8.76E-08	11.7419
134429	STARD4	-1.4323	4.46438	-14.3331	2.95E-09	8.89E-08	11.4642
2260	FGFR1	-0.7732	6.50907	-14.3291	2.96E-09	8.90E-08	11.1141
9689	BZW1	-0.6738	8.89129	-14.2912	3.06E-09	9.16E-08	10.8643
126433	FBXO27	0.85984	6.03466	14.19916	3.31E-09	9.88E-08	11.066
26002	MOXD1	-1.4291	4.7776	-14.1883	3.34E-09	9.92E-08	11.2759
1490	CCN2	1.40854	7.65115	15.74183	3.34E-09	9.92E-08	11.0456
23428	SLC7A8	-2.7066	1.91239	-14.1862	3.34E-09	9.92E-08	11.6557
9358	ITGBL1	1.03536	5.83006	14.17603	3.37E-09	9.99E-08	11.0797
91133	L3MBTL4	-2.0278	2.64652	-14.1489	3.45E-09	1.02E-07	11.6104
9133	CCNB2	-0.8521	5.93668	-14.1356	3.49E-09	1.03E-07	11.0187
5999	RGS4	1.71606	4.0949	14.13091	3.51E-09	1.03E-07	11.3765
3748	KCNC3	-1.3478	4.39002	-14.1217	3.53E-09	1.04E-07	11.2858
3433	IFIT2	1.22599	6.07473	14.11023	3.57E-09	1.04E-07	10.9885
91607	SLFN11	-5.6885	0.95633	-14.0806	3.66E-09	1.07E-07	11.1412
22884	WDR37	1.45305	4.02481	14.07966	3.66E-09	1.07E-07	11.3311
8900	CCNA1	-3.3679	1.53569	-14.0547	3.74E-09	1.09E-07	11.492
120224	TMEM45B	-1.2743	4.48896	-14.0522	3.75E-09	1.09E-07	11.2012
253558	LCLAT1	-0.9678	5.17121	-14.0516	3.75E-09	1.09E-07	11.0676
54510	PCDH18	-1.3224	3.94928	-14.0096	3.89E-09	1.12E-07	11.2679
8334	H2AC6	0.82622	5.75219	13.99922	3.92E-09	1.13E-07	10.9255
2051	EPHB6	-5.3452	-2.03468	-13.9874	3.96E-09	1.14E-07	10.7089
64135	IFIH1	1.57548	3.69371	13.92527	4.18E-09	1.20E-07	11.2588
8440	NCK2	-1.0683	5.32541	-13.9085	4.24E-09	1.21E-07	10.9127
255743	NPNT	-1.9594	3.34245	-13.9077	4.25E-09	1.21E-07	11.3115
10105	PPIF	0.82602	8.34088	13.90008	4.28E-09	1.22E-07	10.5461
84893	FBH1	0.82644	5.66294	13.88927	4.32E-09	1.22E-07	10.8384
5562	PRKAA1	-0.9163	6.70529	-13.8888	4.32E-09	1.22E-07	10.6903

7110	TMF1	1.29514	5.63537	13.88662	4.33E-09	1.22E-07	10.854
53836	GPR87	2.37097	2.4385	13.8792	4.35E-09	1.23E-07	11.3978
5347	PLK1	-1.0229	6.9879	-13.8138	4.61E-09	1.30E-07	10.5898
701	BUB1B	-0.7454	7.14351	-13.8061	4.64E-09	1.30E-07	10.5621
84267	C9orf64	1.41805	3.49878	13.78755	4.72E-09	1.32E-07	11.1612
412	STS	1.19327	4.60941	13.76401	4.81E-09	1.35E-07	10.9191
121551	BTBD11	-7.7901	0.77039	-14.0632	4.84E-09	1.35E-07	10.7628
84733	CBX2	-1.2743	4.12055	-13.7217	5.00E-09	1.39E-07	10.9699
9788	MTSS1	-6.3494	0.29451	-13.7186	5.01E-09	1.39E-07	10.7367
8912	CACNA1H	-4.761	0.327	-13.7108	5.04E-09	1.40E-07	10.7696
4045	LSAMP	-4.7313	0.7029	-13.6785	5.19E-09	1.44E-07	10.8884
11030	RBPMS	-1.0408	5.48134	-13.6676	5.24E-09	1.45E-07	10.6625
8872	CDC123	0.88724	5.88409	13.6132	5.50E-09	1.51E-07	10.549
10890	RAB10	-0.6633	7.89449	-13.6003	5.56E-09	1.53E-07	10.2986
7127	TNFAIP2	1.3761	4.02456	13.59881	5.57E-09	1.53E-07	10.8885
116987	AGAP1	-0.8363	6.33786	-13.5956	5.58E-09	1.53E-07	10.4626
6464	SHC1	-0.6126	7.72623	-13.5952	5.58E-09	1.53E-07	10.3075
1718	DHCR24	-0.5942	8.5527	-13.5899	5.61E-09	1.53E-07	10.2394
2239	GPC4	1.24754	4.36892	13.57618	5.68E-09	1.54E-07	10.7935
144501	KRT80	1.31112	4.52983	13.57429	5.69E-09	1.54E-07	10.7632
5192	PEX10	-1.186	5.13245	-13.5571	5.78E-09	1.56E-07	10.623
54545	MTMR12	-0.7377	6.31128	-13.5161	5.99E-09	1.62E-07	10.39
9784	SNX17	-0.8609	6.64876	-13.5124	6.01E-09	1.62E-07	10.3443
3976	LIF	-0.783	6.58514	-13.5064	6.04E-09	1.62E-07	10.3457
4232	MEST	4.05882	0.46593	13.49289	6.11E-09	1.64E-07	10.7262
51310	SLC22A17	-1.3758	3.96373	-13.4868	6.15E-09	1.64E-07	10.7872
11253	MAN1B1	0.77959	6.67172	13.47717	6.20E-09	1.65E-07	10.3094
4208	MEF2C	-1.5112	3.70625	-13.457	6.31E-09	1.68E-07	10.8151
10575	CCT4	-0.5949	7.80106	-13.4563	6.32E-09	1.68E-07	10.1693
11190	CEP250	-0.7473	7.28772	-13.4222	6.51E-09	1.73E-07	10.1856
57153	SLC44A2	0.66122	7.78455	13.3937	6.68E-09	1.77E-07	10.1127
124359	CDYL2	2.06949	3.06758	13.39171	6.69E-09	1.77E-07	10.896
22885	ABLIM3	-0.6215	7.72758	-13.3841	6.74E-09	1.78E-07	10.1068
3170	FOXA2	-1.0467	5.70924	-13.3744	6.80E-09	1.79E-07	10.3493
8549	LGR5	-1.2069	6.04128	-13.3737	6.80E-09	1.79E-07	10.3018
57574	MARCHF4	5.96569	-0.57414	13.32739	7.09E-09	1.86E-07	10.412
10133	OPTN	1.22166	4.64761	13.32107	7.13E-09	1.86E-07	10.498
339400	FLG-AS1	5.26442	-0.92842	13.29867	7.28E-09	1.90E-07	10.3459
6907	TBL1X	0.62459	7.11645	13.29604	7.29E-09	1.90E-07	10.0836
5019	OXCT1	-1.0725	5.27105	-13.2677	7.48E-09	1.94E-07	10.3207
158	ADSL	-0.8119	6.12139	-13.2635	7.51E-09	1.95E-07	10.1763
29102	DROSHA	-0.702	6.66226	-13.2623	7.52E-09	1.95E-07	10.1023
501	ALDH7A1	-0.6803	6.45654	-13.2399	7.67E-09	1.98E-07	10.1064
254887	ZDHHC23	1.97399	3.03419	13.22464	7.78E-09	2.00E-07	10.7419
5045	FURIN	0.84691	6.08276	13.21876	7.82E-09	2.01E-07	10.1431
9053	MAP7	-3.7194	1.39187	-13.1871	8.05E-09	2.07E-07	10.7307
7298	TYMS	-0.5841	8.76134	-13.176	8.13E-09	2.08E-07	9.8285
399694	SHC4	-1.1233	4.43174	-13.1736	8.15E-09	2.08E-07	10.3884
79974	CPED1	2.26972	2.14334	13.16772	8.19E-09	2.09E-07	10.7746
120	ADD3	-1.0233	5.46544	-13.1494	8.33E-09	2.12E-07	10.1725

773	CACNA1A	-3.8602	1.07784	-13.1415	8.39E-09	2.13E-07	10.6348
341208	HEPHL1	-3.1196	1.03279	-13.1061	8.67E-09	2.19E-07	10.6398
10669	CGREF1	-1.0931	4.40484	-13.1019	8.70E-09	2.19E-07	10.3235
64897	C12orf43	0.78495	5.66487	13.1004	8.71E-09	2.19E-07	10.0911
55146	ZDHHC4	-1.012	5.27976	-13.0867	8.82E-09	2.21E-07	10.1432
138151	NACC2	0.77705	5.73854	13.08275	8.85E-09	2.22E-07	10.0621
83999	KREMEN1	-1.1865	3.86137	-13.0539	9.09E-09	2.27E-07	10.3896
1281	COL3A1	-1.9217	6.13843	-14.6636	9.18E-09	2.29E-07	10.1841
5727	PTCH1	1.66545	3.71696	13.04161	9.19E-09	2.29E-07	10.4369
577	ADGRB3	-1.4364	3.6114	-13.0287	9.30E-09	2.31E-07	10.4244
4862	NPAS2	-0.6257	8.07109	-12.993	9.61E-09	2.38E-07	9.7
83998	REG4	-2.6612	1.56719	-12.9829	9.70E-09	2.40E-07	10.6012
2318	FLNC	0.67322	7.82354	12.97555	9.77E-09	2.40E-07	9.7042
1382	CRABP2	-0.8608	5.21238	-12.9754	9.77E-09	2.40E-07	10.0433
221935	SDK1	-2.2514	2.20616	-12.9718	9.80E-09	2.40E-07	10.5899
6120	RPE	-0.7845	6.13838	-12.9648	9.87E-09	2.41E-07	9.88332
8543	LMO4	-0.847	5.34173	-12.9567	9.94E-09	2.42E-07	10.002
11078	TRIOBP	-0.6575	6.76604	-12.9567	9.94E-09	2.42E-07	9.79205
7852	CXCR4	-4.4379	-0.08961	-12.941	1.01E-08	2.45E-07	10.1042
109	ADCY3	-0.7624	6.39692	-12.9368	1.01E-08	2.45E-07	9.81994
1183	CLCN4	0.77574	5.55632	12.93505	1.01E-08	2.45E-07	9.94712
5140	PDE3B	-4.4767	-0.39537	-12.9214	1.03E-08	2.48E-07	10.0644
9901	SRGAP3	-1.4638	3.67671	-12.9179	1.03E-08	2.48E-07	10.307
643988	FNDC10	-5.2091	-1.57598	-12.87	1.08E-08	2.59E-07	9.95677
860	RUNX2	-2.4814	1.93018	-12.8586	1.09E-08	2.61E-07	10.4962
26012	NSMF	0.77225	6.15275	12.8547	1.09E-08	2.61E-07	9.77591
6940	ZNF354A	-1.1559	4.28723	-12.853	1.09E-08	2.61E-07	10.1071
509	ATP5F1C	0.71176	6.61442	12.85165	1.10E-08	2.61E-07	9.70943
10715	CERS1	-4.6903	-0.77801	-12.8461	1.10E-08	2.62E-07	9.98635
26873	OPLAH	-1.0703	4.24217	-12.838	1.11E-08	2.63E-07	10.0986
10449	ACAA2	-1.7855	3.00435	-12.8312	1.12E-08	2.65E-07	10.3574
6713	SQLE	-0.6576	6.75307	-12.8042	1.14E-08	2.71E-07	9.64292
60481	ELOVL5	-0.6344	8.28758	-12.7517	1.20E-08	2.83E-07	9.44469
151887	CCDC80	0.88211	4.9404	12.7478	1.21E-08	2.84E-07	9.87408
9672	SDC3	-0.7353	5.97367	-12.747	1.21E-08	2.84E-07	9.6914
8325	FZD8	0.78843	8.24486	12.73672	1.22E-08	2.86E-07	9.43441
5603	MAPK13	1.4005	3.87627	12.72985	1.23E-08	2.87E-07	10.0895
3956	LGALS1	-0.7017	9.14227	-12.7227	1.24E-08	2.89E-07	9.3571
10085	EDIL3	-0.8556	7.39029	-12.7178	1.24E-08	2.89E-07	9.48866
90417	KNSTRN	-0.6596	6.42023	-12.7145	1.25E-08	2.90E-07	9.59559
23277	CLUH	0.7259	6.52275	12.70804	1.25E-08	2.91E-07	9.57846
11015	KDELR3	-0.7067	6.17572	-12.6817	1.28E-08	2.97E-07	9.59651
51182	HSPA14	0.90302	5.05795	12.68075	1.29E-08	2.97E-07	9.78638
27075	TSPAN13	-1.0252	4.90601	-12.6724	1.30E-08	2.99E-07	9.80429
151742	PPM1L	-1.8926	2.89325	-12.659	1.31E-08	3.02E-07	10.2136
23185	LARP4B	0.69753	5.91426	12.6578	1.31E-08	3.02E-07	9.61409
4552	MTRR	-0.8439	5.05941	-12.6546	1.32E-08	3.03E-07	9.75372
3653	IPW	-0.9045	4.8262	-12.6409	1.33E-08	3.05E-07	9.78477
3778	KCNMA1	-0.6872	9.06002	-12.6393	1.34E-08	3.05E-07	9.27862
10205	MPZL2	-5.0506	-0.11179	-12.6377	1.34E-08	3.05E-07	9.87548

92822	ZNF276	0.86123	5.49029	12.63743	1.34E-08	3.05E-07	9.66525
7022	TFAP2C	-0.8526	5.39862	-12.6243	1.36E-08	3.09E-07	9.66321
10184	LHFPL2	-0.9279	5.11293	-12.6211	1.36E-08	3.09E-07	9.71238
4627	MYH9	-0.6516	9.53713	-12.6199	1.36E-08	3.09E-07	9.22965
114804	RNF157	-0.8278	6.01343	-12.6014	1.38E-08	3.13E-07	9.54144
7504	XK	-1.8993	2.91943	-12.5873	1.40E-08	3.17E-07	10.1401
7204	TRIO	-0.9706	7.31971	-12.6305	1.41E-08	3.18E-07	9.36828
85315	PAQR8	-1.4744	3.06943	-12.5789	1.41E-08	3.18E-07	10.0895
128239	IQGAP3	-0.7406	6.38682	-12.5696	1.43E-08	3.20E-07	9.4554
729582	DIRC3	3.73179	0.39068	12.55979	1.44E-08	3.23E-07	9.9876
4190	MDH1	-0.6512	7.38458	-12.5467	1.46E-08	3.26E-07	9.31504
51474	LIMA1	0.79931	6.70708	12.53076	1.48E-08	3.31E-07	9.37812
728577	CNTNAP3B	-1.6557	2.90842	-12.5288	1.48E-08	3.31E-07	10.0749
26018	LRIG1	-4.8262	0.18873	-12.5239	1.49E-08	3.31E-07	9.81486
970	CD70	-3.2101	1.38239	-12.5178	1.50E-08	3.32E-07	10.1574
100216001	MANCR	0.76344	5.65694	12.51306	1.51E-08	3.33E-07	9.51022
2690	GHR	-1.1022	4.54439	-12.5095	1.51E-08	3.34E-07	9.71343
6319	SCD	-0.616	9.39172	-12.4973	1.53E-08	3.37E-07	9.1141
55002	TMCO3	-0.648	6.54995	-12.4886	1.54E-08	3.39E-07	9.35134
26064	RAI14	-0.726	6.22423	-12.4854	1.55E-08	3.40E-07	9.39232
54756	IL17RD	-0.9995	4.51271	-12.4799	1.55E-08	3.41E-07	9.6868
5137	PDE1C	0.95303	4.47678	12.47533	1.56E-08	3.42E-07	9.69377
8140	SLC7A5	0.73043	8.46956	12.47378	1.56E-08	3.42E-07	9.15225
9853	RUSC2	0.85954	5.06969	12.46848	1.57E-08	3.43E-07	9.56992
4536	ND2	0.74012	10.3841	12.42843	1.63E-08	3.56E-07	8.98791
348262	MCRIP1	-1.079	5.46615	-12.4192	1.65E-08	3.58E-07	9.44979
9208	LRRFIP1	-0.8216	6.9302	-12.4022	1.67E-08	3.63E-07	9.21896
253868	MIR1-1HG-AS1	-4.9852	-1.68921	-12.3774	1.71E-08	3.71E-07	9.55129
55629	PNRC2	-0.6777	6.03674	-12.3735	1.72E-08	3.72E-07	9.30463
10807	ENTR1	1.01164	5.31696	12.35827	1.75E-08	3.76E-07	9.41748
5891	MOK	-0.9842	4.67656	-12.3574	1.75E-08	3.76E-07	9.52991
116225	ZMYND19	1.11889	4.2801	12.35231	1.76E-08	3.77E-07	9.61598
1653	DDX1	-0.6448	6.76135	-12.3495	1.76E-08	3.78E-07	9.18327
84991	RBM17	0.7648	5.64077	12.33809	1.78E-08	3.81E-07	9.33507
27239	GPR162	-5.0608	-1.65314	-12.3225	1.81E-08	3.86E-07	9.51471
162394	SLFN5	1.08118	5.84923	12.31651	1.82E-08	3.87E-07	9.2877
10643	IGF2BP3	0.82379	5.53298	12.31634	1.82E-08	3.87E-07	9.33205
81704	DOCK8	-4.1451	0.51225	-12.3077	1.83E-08	3.89E-07	9.78537
8321	FZD1	-0.9652	4.31159	-12.2884	1.87E-08	3.96E-07	9.53186
8602	NOP14	0.73266	6.01838	12.28527	1.87E-08	3.97E-07	9.22124
1470	CST2	-2.9859	1.04889	-12.2773	1.89E-08	3.99E-07	9.91319
29968	PSAT1	0.75271	6.498	12.27396	1.89E-08	4.00E-07	9.14188
29803	REPIN1	0.62422	7.16946	12.26552	1.91E-08	4.02E-07	9.05201
90268	OTULIN	-0.8543	5.74556	-12.2637	1.91E-08	4.02E-07	9.23975
151473	SLC16A14	-0.9177	5.015	-12.2629	1.91E-08	4.02E-07	9.36729
84436	ZNF528	-2.4925	1.49339	-12.2559	1.93E-08	4.04E-07	9.93014
4147	MATN2	0.61582	6.81257	12.25037	1.94E-08	4.05E-07	9.07677
2770	GNAI1	-0.9548	5.16118	-12.2241	1.99E-08	4.15E-07	9.30137
26011	TENM4	-1.0194	5.8525	-12.2147	2.01E-08	4.18E-07	9.17623
4322	MMP13	1.21917	5.35833	12.19873	2.04E-08	4.23E-07	9.25347

4643	MYO1E	-0.7217	6.50151	-12.184	2.07E-08	4.29E-07	9.04614
9232	PTTG1	-0.9112	6.62833	-12.1762	2.08E-08	4.31E-07	9.02375
3755	KCNG1	-1.0903	4.29098	-12.1733	2.09E-08	4.32E-07	9.42237
5881	RAC3	-1.2232	4.38025	-12.1493	2.14E-08	4.41E-07	9.38414
481	ATP1B1	0.82374	5.59313	12.14297	2.15E-08	4.43E-07	9.14347
57646	USP28	-0.683	5.85301	-12.1401	2.16E-08	4.43E-07	9.09209
195828	ZNF367	0.80627	5.29735	12.13464	2.17E-08	4.45E-07	9.18545
80851	SH3BP5L	0.76708	7.02592	12.13162	2.17E-08	4.45E-07	8.93106
3880	KRT19	-0.6833	8.1872	-12.1257	2.19E-08	4.47E-07	8.81398
4217	MAP3K5	-1.0427	4.47281	-12.1219	2.20E-08	4.48E-07	9.33092
22949	PTGR1	0.72036	6.05253	12.11571	2.21E-08	4.50E-07	9.04062
3638	INSIG1	-0.7886	5.15425	-12.1075	2.23E-08	4.53E-07	9.17861
221955	DAGLB	1.06467	5.17525	12.10048	2.24E-08	4.55E-07	9.17977
2037	EPB41L2	-0.6888	6.77065	-12.0844	2.28E-08	4.61E-07	8.90841
114908	TMEM123	-0.5945	8.56339	-12.0763	2.30E-08	4.64E-07	8.73452
23548	TTC33	-1.1812	4.40838	-12.0724	2.30E-08	4.64E-07	9.29785
9120	SLC16A6	3.34676	1.02704	12.07064	2.31E-08	4.64E-07	9.70072
4726	NDUFS6	-0.9759	6.00121	-12.0706	2.31E-08	4.64E-07	9.00266
151246	SGO2	-0.7087	6.5088	-12.0514	2.35E-08	4.72E-07	8.90704
10777	ARPP21	-4.0902	0.18456	-12.0412	2.38E-08	4.76E-07	9.47702
23566	LPAR3	-0.7535	5.49653	-12.0336	2.39E-08	4.78E-07	9.04034
25896	INTS7	0.67679	5.824	12.03047	2.40E-08	4.79E-07	8.98587
4192	MDK	1.1192	7.80984	13.04378	2.43E-08	4.84E-07	8.88044
26999	CYFIP2	-0.7616	5.53726	-12.0095	2.45E-08	4.86E-07	9.00846
51421	AMOTL2	0.98174	6.11767	11.99894	2.48E-08	4.91E-07	8.91464
10846	PDE10A	-1.6574	3.97726	-11.98	2.52E-08	4.99E-07	9.31498
4059	BCAM	-0.7944	6.60798	-11.974	2.54E-08	5.01E-07	8.81396
53918	PELO	-1.0757	4.80402	-11.9634	2.56E-08	5.06E-07	9.10176
56099	PCDHGB7	-5.6839	-1.09674	-11.9533	2.59E-08	5.10E-07	9.27776
55165	CEP55	-0.6049	6.44011	-11.927	2.66E-08	5.23E-07	8.78441
5087	PBX1	-1.304	4.40156	-11.9201	2.68E-08	5.26E-07	9.14551
10123	ARL4C	-0.9393	4.25213	-11.8921	2.75E-08	5.40E-07	9.13372
11065	UBE2C	-0.8305	7.33496	-11.8759	2.80E-08	5.47E-07	8.62744
113763	ZBED6CL	1.76893	2.31455	11.87122	2.81E-08	5.49E-07	9.50947
79695	GALNT12	-3.8893	0.03977	-11.8671	2.82E-08	5.50E-07	9.30196
85236	H2BC12	0.92328	7.70031	11.99541	2.85E-08	5.54E-07	8.59671
5972	REN	4.75843	-1.18414	11.85365	2.86E-08	5.55E-07	9.16358
90637	ZFAND2A	1.10799	4.11801	11.84119	2.90E-08	5.62E-07	9.12204
8710	SERPINB7	0.93024	4.92808	11.83788	2.90E-08	5.63E-07	8.94708
83660	TLN2	-0.8252	5.08912	-11.8125	2.98E-08	5.76E-07	8.88196
30846	EHD2	-2.0102	1.90375	-11.8075	2.99E-08	5.78E-07	9.48134
3553	IL1B	1.66532	3.61644	11.78807	3.05E-08	5.88E-07	9.20171
164832	LONRF2	-2.2826	2.07081	-11.7743	3.10E-08	5.94E-07	9.44136
81611	ANP32E	-0.6587	6.76888	-11.7682	3.11E-08	5.97E-07	8.57465
56975	FAM20C	-0.8132	5.60413	-11.7637	3.13E-08	5.98E-07	8.73866
55008	HERC6	1.3048	3.69717	11.75773	3.15E-08	6.00E-07	9.13389
9830	TRIM14	0.66025	5.90003	11.75212	3.16E-08	6.03E-07	8.67976
54476	RNF216	0.58171	6.49526	11.75104	3.17E-08	6.03E-07	8.5918
5074	PAWR	0.82188	5.3022	11.7315	3.23E-08	6.14E-07	8.76124
127845	GOLT1A	3.03989	1.01146	11.72303	3.26E-08	6.18E-07	9.38165

10413	YAP1	-0.7333	7.51192	-11.7055	3.32E-08	6.28E-07	8.42719
60509	AGBL5	-0.7903	5.84017	-11.6999	3.34E-08	6.30E-07	8.63202
1010	CDH12	-3.1573	1.61904	-11.6922	3.36E-08	6.34E-07	9.38273
162461	TMEM92	1.6709	2.44201	11.66812	3.44E-08	6.48E-07	9.28358
9526	MPDU1	0.75781	6.06888	11.66759	3.45E-08	6.48E-07	8.56547
92249	LINC01278	0.79565	4.96154	11.66363	3.46E-08	6.49E-07	8.75136
23593	HEBP2	-0.7523	5.05581	-11.6474	3.52E-08	6.59E-07	8.71076
390874	ONECUT3	-4.8585	-1.75486	-11.646	3.52E-08	6.59E-07	8.93625
54985	HCFC1R1	-1.0377	4.89386	-11.6349	3.56E-08	6.65E-07	8.73504
89886	SLAMF9	-3.5901	-0.11218	-11.627	3.59E-08	6.70E-07	9.07396
8503	PIK3R3	-0.8426	6.22913	-11.6255	3.60E-08	6.70E-07	8.49488
3768	KCNJ12	-5.2011	-0.80878	-11.6179	3.62E-08	6.74E-07	8.99205
1601	DAB2	-0.7631	6.01164	-11.6169	3.63E-08	6.74E-07	8.51612
105373989	LOC105373989	-1.8765	3.08588	-11.6153	3.63E-08	6.74E-07	9.12488
9832	JAKMIP2	-2.4089	1.44969	-11.6062	3.67E-08	6.79E-07	9.29734
100287284	MANSC4	1.59386	2.75401	11.60075	3.69E-08	6.81E-07	9.16465
27158	NDOR1	0.80135	5.60318	11.59812	3.70E-08	6.82E-07	8.5655
1033	CDKN3	-0.6317	7.14144	-11.5927	3.72E-08	6.84E-07	8.3427
64856	VWA1	-1.9815	2.44442	-11.5885	3.73E-08	6.86E-07	9.20411
283987	HID1	-0.7582	5.63064	-11.5834	3.75E-08	6.88E-07	8.54007
101410538	MMP24OS	-0.8853	5.03052	-11.5832	3.75E-08	6.88E-07	8.64937
9444	QKI	-0.6454	7.51404	-11.5783	3.77E-08	6.89E-07	8.28909
26271	FBXO5	-0.7919	5.52315	-11.5782	3.77E-08	6.89E-07	8.55312
100507012	BMPR1B-DT	-4.3072	-0.56527	-11.5766	3.78E-08	6.90E-07	8.94446
10097	ACTR2	-0.7123	8.55035	-11.5668	3.82E-08	6.94E-07	8.19315
5567	PRKACB	-0.7767	5.4248	-11.5608	3.84E-08	6.98E-07	8.55119
9063	PIAS2	-0.9632	4.49312	-11.5504	3.88E-08	7.04E-07	8.72209
23683	PRKD3	-0.7589	6.15902	-11.5452	3.90E-08	7.07E-07	8.41692
10957	PNRC1	-0.7077	5.48774	-11.5394	3.92E-08	7.10E-07	8.51584
6541	SLC7A1	0.7245	6.37865	11.53833	3.93E-08	7.10E-07	8.38041
55388	MCM10	0.9743	4.41273	11.5373	3.93E-08	7.10E-07	8.73105
1803	DPP4	-0.6187	7.14711	-11.512	4.04E-08	7.27E-07	8.25439
29015	SLC43A3	-0.7728	5.50964	-11.492	4.12E-08	7.41E-07	8.46167
285590	SH3PXD2B	-0.8215	4.94735	-11.4827	4.16E-08	7.46E-07	8.5552
6640	SNTA1	-0.7493	5.64498	-11.4827	4.16E-08	7.46E-07	8.42832
54757	FAM20A	-3.3935	1.21008	-11.4614	4.25E-08	7.61E-07	9.13792
115908	CTHRC1	0.77161	5.07121	11.45038	4.30E-08	7.69E-07	8.4999
2192	FBLN1	-0.8445	6.26296	-11.4369	4.36E-08	7.77E-07	8.28545
202915	TMEM184A	1.06814	4.0749	11.43582	4.36E-08	7.77E-07	8.69769
1602	DACH1	-2.5166	1.26176	-11.4179	4.44E-08	7.90E-07	9.10656
7272	TTK	-0.6709	6.24325	-11.4016	4.52E-08	8.03E-07	8.24727
51200	CPA4	1.15217	4.24362	11.40023	4.52E-08	8.03E-07	8.62622
196264	MPZL3	-2.5179	1.21259	-11.3987	4.53E-08	8.03E-07	9.08623
55274	PHF10	-0.8704	4.86227	-11.3866	4.59E-08	8.12E-07	8.46826
3673	ITGA2	-1.0306	5.84326	-11.3638	4.70E-08	8.30E-07	8.27281
55717	WDR11	-0.7602	5.1542	-11.3603	4.71E-08	8.31E-07	8.38163
29952	DPP7	0.88242	6.14203	11.34358	4.80E-08	8.42E-07	8.20515
55120	FANCL	-1.1546	3.56012	-11.342	4.80E-08	8.42E-07	8.70364
80310	PDGFD	-1.6679	2.98679	-11.3418	4.81E-08	8.42E-07	8.84349
163404	PLPPR5	-1.2596	3.35756	-11.3412	4.81E-08	8.42E-07	8.74997

8863	PER3	-0.9305	4.58302	-11.3327	4.85E-08	8.49E-07	8.46638
2309	FOXO3	-0.7585	5.34207	-11.3262	4.88E-08	8.53E-07	8.30992
8991	SELENBP1	1.23384	3.33065	11.32073	4.91E-08	8.57E-07	8.74092
284	ANGPT1	-4.2105	-0.14771	-11.3128	4.95E-08	8.61E-07	8.74167
653653	LOC653653	-1.131	3.50708	-11.3118	4.96E-08	8.61E-07	8.68123
151636	DTX3L	0.81958	6.21935	11.30541	4.99E-08	8.66E-07	8.15023
7483	WNT9A	0.85406	5.12281	11.28911	5.07E-08	8.79E-07	8.31605
10486	CAP2	-0.6493	5.93253	-11.2847	5.10E-08	8.82E-07	8.16403
1062	CENPE	-1.1156	6.56709	-11.8263	5.14E-08	8.89E-07	8.15654
5257	PHKB	-0.6056	6.48004	-11.271	5.17E-08	8.92E-07	8.07071
26049	FAM169A	-1.1784	3.99058	-11.2646	5.21E-08	8.97E-07	8.52662
3977	LIFR	-1.0284	6.08835	-11.2982	5.23E-08	9.01E-07	8.12583
25917	THUMPD3	-0.8186	5.75517	-11.2512	5.28E-08	9.07E-07	8.15799
8519	IFITM1	1.791	4.69179	12.15426	5.29E-08	9.08E-07	8.52983
2256	FGF11	-2.0087	2.18076	-11.2423	5.33E-08	9.12E-07	8.87369
665	BNIP3L	-0.632	7.39177	-11.2407	5.34E-08	9.12E-07	7.93084
20	ABCA2	0.6212	5.78733	11.21842	5.46E-08	9.31E-07	8.11638
7226	TRPM2	1.10112	3.97601	11.21518	5.48E-08	9.33E-07	8.4803
8444	DYRK3	1.10021	3.81699	11.18321	5.67E-08	9.63E-07	8.48047
5393	EXOSC9	0.85532	4.98435	11.18163	5.68E-08	9.63E-07	8.22383
8491	MAP4K3	-0.7484	5.87739	-11.1772	5.70E-08	9.66E-07	8.05518
51226	COPZ2	3.83905	0.60539	11.174	5.72E-08	9.68E-07	8.77106
55076	TMEM45A	-0.6964	6.38729	-11.148	5.88E-08	9.92E-07	7.94733
55829	SELENOS	0.62993	5.67815	11.14437	5.90E-08	9.94E-07	8.05199
9550	ATP6V1G1	0.6548	6.60548	11.12636	6.01E-08	1.01E-06	7.89585
23071	ERP44	0.65927	6.04131	11.12408	6.03E-08	1.01E-06	7.9725
8417	STX7	-0.9124	5.86134	-11.1202	6.05E-08	1.01E-06	7.99747
10950	BTG3	-1.2588	3.54298	-11.1168	6.07E-08	1.02E-06	8.46536
3606	IL18	0.65933	5.58911	11.11229	6.10E-08	1.02E-06	8.03165
23184	MESD	0.58989	6.20811	11.09034	6.25E-08	1.04E-06	7.90886
9849	ZNF518A	2.97895	0.56414	11.08932	6.25E-08	1.04E-06	8.71811
196403	DTX3	1.00544	4.16496	11.07849	6.32E-08	1.05E-06	8.28315
57537	SORCS2	-5.0326	-0.88971	-11.0644	6.42E-08	1.06E-06	8.48602
23198	PSME4	-0.6278	6.91709	-11.0603	6.45E-08	1.07E-06	7.78135
80024	SLC8B1	0.66022	5.64185	11.05574	6.48E-08	1.07E-06	7.95952
11332	ACOT7	-0.7734	7.01637	-11.0479	6.53E-08	1.08E-06	7.75722
55964	SEPTIN3	-1.8182	2.01312	-11.0428	6.57E-08	1.08E-06	8.67447
140733	MACROD2	-2.036	1.92567	-11.0279	6.67E-08	1.10E-06	8.67181
2539	G6PD	-0.6956	7.80619	-11.0245	6.69E-08	1.10E-06	7.65112
10911	UTS2	-4.0882	-0.42889	-11.0201	6.73E-08	1.10E-06	8.44435
128434	VSTM2L	-2.7095	1.30998	-11.0145	6.77E-08	1.11E-06	8.69348
8573	CASK	-0.7009	6.26533	-11.0006	6.87E-08	1.12E-06	7.79889
6832	SUPV3L1	0.59335	6.28192	11.0003	6.87E-08	1.12E-06	7.79713
54480	CHPF2	0.68676	5.50694	10.99102	6.94E-08	1.13E-06	7.91026
2817	GPC1	-0.7942	6.15268	-10.9898	6.94E-08	1.13E-06	7.80426
1063	CENPF	-0.7751	7.57196	-10.9851	6.98E-08	1.14E-06	7.62837
27436	EML4	-0.6413	5.98913	-10.9828	7.00E-08	1.14E-06	7.81838
23270	TSPYL4	-0.8734	5.01215	-10.9719	7.08E-08	1.15E-06	7.97954
8237	USP11	-0.605	7.331	-10.9693	7.10E-08	1.15E-06	7.63264
3014	H2AX	-0.8174	7.00137	-10.9685	7.10E-08	1.15E-06	7.66999

3708	ITPR1	-1.1135	5.76565	-11.0822	7.14E-08	1.15E-06	7.86124
84818	IL17RC	-0.7373	6.28726	-10.9542	7.21E-08	1.16E-06	7.74383
54942	ABITRAM	0.82155	4.85612	10.94339	7.30E-08	1.17E-06	7.9813
7089	TLE2	-0.9914	4.40948	-10.9359	7.35E-08	1.18E-06	8.06363
55619	DOCK10	0.71971	5.59457	10.93254	7.38E-08	1.18E-06	7.82993
92154	MTSS2	0.6262	6.32798	10.92354	7.45E-08	1.19E-06	7.70427
91947	ARRDC4	5.00629	-0.81195	10.915	7.52E-08	1.20E-06	8.3545
8970	H2BC11	1.17606	3.35996	10.91394	7.53E-08	1.20E-06	8.28491
10577	NPC2	0.62309	6.16472	10.91232	7.54E-08	1.20E-06	7.71501
51765	STK26	0.7157	5.83106	10.90879	7.57E-08	1.21E-06	7.76408
91754	NEK9	-0.6933	6.32083	-10.9009	7.63E-08	1.22E-06	7.67821
7295	TXN	0.63833	7.40677	10.89599	7.67E-08	1.22E-06	7.54388
9320	TRIP12	-0.698	7.90711	-10.8956	7.68E-08	1.22E-06	7.49659
51374	ATRAID	-0.6132	6.66124	-10.894	7.69E-08	1.22E-06	7.62458
5797	PTPRM	-0.7176	7.17931	-10.8913	7.71E-08	1.22E-06	7.56132
3855	KRT7	1.64141	5.51553	12.01269	7.76E-08	1.23E-06	7.99428
57462	MYORG	0.93507	4.05025	10.88433	7.77E-08	1.23E-06	8.08829
134957	STXBP5	-0.8743	5.69494	-10.867	7.92E-08	1.25E-06	7.7381
781	CACNA2D1	-2.8923	1.10591	-10.8626	7.95E-08	1.25E-06	8.53154
162963	ZNF610	3.70577	-0.68941	10.8593	7.98E-08	1.25E-06	8.27521
10920	COPS8	-0.735	6.23912	-10.8387	8.16E-08	1.28E-06	7.61942
9792	SERTAD2	-0.5865	7.60233	-10.8385	8.16E-08	1.28E-06	7.45727
7378	UPP1	0.99684	5.11604	10.83682	8.18E-08	1.28E-06	7.81553
4668	NAGA	-0.6629	6.11897	-10.8355	8.19E-08	1.28E-06	7.63214
81037	CLPTM1L	-1.0452	5.18891	-10.8346	8.19E-08	1.28E-06	7.79457
2281	FKBP1B	-3.1155	0.01305	-10.8286	8.25E-08	1.28E-06	8.38221
64432	MRPS25	0.7415	5.24511	10.82718	8.26E-08	1.28E-06	7.77278
23302	WSCD1	-3.9062	-0.27578	-10.8078	8.43E-08	1.31E-06	8.26285
2720	GLB1	0.60747	6.02078	10.78278	8.66E-08	1.34E-06	7.58858
9875	URB1	0.58857	6.34448	10.77874	8.70E-08	1.35E-06	7.53625
816	CAMK2B	-0.897	4.15067	-10.7657	8.83E-08	1.37E-06	7.92358
283554	GPR137C	-1.0373	4.07739	-10.7562	8.92E-08	1.38E-06	7.93384
126282	TNFAIP8L1	-1.1723	4.33473	-10.7543	8.93E-08	1.38E-06	7.87988
51116	MRPS2	0.93693	5.92367	10.75349	8.94E-08	1.38E-06	7.57741
7068	THRB	-4.8262	-1.77026	-10.7512	8.96E-08	1.38E-06	8.13303
576	ADGRB2	-0.8906	4.30287	-10.7414	9.06E-08	1.39E-06	7.86259
221061	FAM171A1	0.59792	5.94282	10.73306	9.14E-08	1.40E-06	7.54341
55143	CDCA8	-0.6443	6.36985	-10.7314	9.16E-08	1.40E-06	7.47707
8492	PRSS12	1.99939	1.5589	10.72678	9.20E-08	1.41E-06	8.37703
79643	CHMP6	-0.8777	4.3474	-10.7255	9.22E-08	1.41E-06	7.83467
9404	LPXN	1.30485	4.01629	10.72127	9.26E-08	1.41E-06	7.92883
23242	COBL	-0.9969	3.81966	-10.7122	9.35E-08	1.42E-06	7.93958
51514	DTL	0.68399	6.10216	10.71131	9.36E-08	1.42E-06	7.49556
148479	PHF13	-0.6006	6.11097	-10.7092	9.38E-08	1.42E-06	7.48764
5744	PTHLH	-1.0244	5.62966	-10.7041	9.43E-08	1.43E-06	7.56603
51308	REEP2	-0.9247	4.27049	-10.6998	9.48E-08	1.43E-06	7.82286
55686	MREG	0.9809	4.29351	10.69682	9.51E-08	1.43E-06	7.82322
5324	PLAG1	-1.9445	1.82762	-10.6938	9.54E-08	1.44E-06	8.31675
2982	GUCY1A1	-3.4117	1.88064	-10.7348	9.58E-08	1.44E-06	8.34393
90203	SNX21	-0.6047	6.09214	-10.684	9.64E-08	1.44E-06	7.46137

55646	LYAR	0.78736	4.94257	10.68326	9.65E-08	1.44E-06	7.66704
9050	PSTPIP2	4.76257	-1.71321	10.68319	9.65E-08	1.44E-06	8.07752
200504	GKN2	2.88449	0.59843	10.68123	9.67E-08	1.44E-06	8.31139
64857	PLEKHG2	-0.7269	4.92303	-10.6807	9.68E-08	1.44E-06	7.66187
11244	ZHX1	0.6268	6.16004	10.6793	9.69E-08	1.44E-06	7.44891
80204	FBXO11	-0.6346	6.43546	-10.6691	9.80E-08	1.46E-06	7.39632
54084	TSPEAR	-1.7805	2.7664	-10.6648	9.84E-08	1.46E-06	8.14545
55632	G2E3	-0.8452	5.36976	-10.6602	9.89E-08	1.47E-06	7.55668
9631	NUP155	-0.6734	6.1615	-10.6469	1.00E-07	1.49E-06	7.40927
22871	NLGN1	-1.2815	3.68082	-10.6465	1.00E-07	1.49E-06	7.90836
5590	PRKCZ	-0.7439	5.16365	-10.6378	1.01E-07	1.50E-06	7.56667
54798	DCHS2	-5.3663	-0.56212	-10.6308	1.02E-07	1.51E-06	8.096
80177	MYCT1	4.09351	-0.11297	10.62102	1.03E-07	1.53E-06	8.08621
9582	APOBEC3B	-0.8481	4.75694	-10.6077	1.05E-07	1.55E-06	7.61346
90233	ZNF551	0.94672	4.11791	10.60392	1.05E-07	1.55E-06	7.75378
11076	TPPP	-1.2193	3.12909	-10.5971	1.06E-07	1.56E-06	7.97055
8532	CPZ	0.74553	4.78831	10.59002	1.07E-07	1.56E-06	7.58877
4900	NRGN	-1.1986	5.27695	-10.7735	1.07E-07	1.57E-06	7.5293
11091	WDR5	0.60614	6.22745	10.58442	1.07E-07	1.57E-06	7.32876
284904	SEC14L4	-1.0469	3.50338	-10.5619	1.10E-07	1.60E-06	7.84101
119	ADD2	0.73799	5.38776	10.55938	1.10E-07	1.61E-06	7.43816
1891	ECH1	-0.6008	6.17843	-10.5493	1.12E-07	1.62E-06	7.29241
57109	REXO4	0.65723	5.79039	10.54087	1.13E-07	1.63E-06	7.34621
151313	FAHD2B	-1.4063	2.92017	-10.5359	1.13E-07	1.64E-06	7.95264
148156	ZNF558	-0.8282	4.31404	-10.522	1.15E-07	1.66E-06	7.60571
4128	MAOA	-3.7034	-0.09386	-10.5143	1.16E-07	1.67E-06	8.02053
92659	MAFG-DT	-1.6119	2.69077	-10.5044	1.17E-07	1.69E-06	7.96982
5810	RAD1	-0.6626	5.24369	-10.5	1.18E-07	1.69E-06	7.39016
23203	PMPCA	0.69797	5.58761	10.49388	1.19E-07	1.70E-06	7.32587
219790	RTKN2	-0.8731	5.56543	-10.4873	1.20E-07	1.71E-06	7.32164
10675	CSPG5	-2.3199	1.97045	-10.4802	1.20E-07	1.73E-06	8.07442
9497	SLC4A7	-1.0796	6.61988	-11.2069	1.22E-07	1.74E-06	7.26595
57713	SFMBT2	-1.5003	2.67801	-10.4662	1.22E-07	1.74E-06	7.92481
196410	METTL7B	1.02924	3.93907	10.46424	1.23E-07	1.75E-06	7.63628
79649	MAP7D3	2.09267	1.33829	10.4581	1.23E-07	1.76E-06	8.09532
23102	TBC1D2B	0.65031	5.4872	10.45077	1.24E-07	1.77E-06	7.29169
55704	CCDC88A	-1.1293	6.58199	-11.2903	1.28E-07	1.80E-06	7.24461
80380	PDCD1LG2	2.01633	1.6773	10.42624	1.28E-07	1.80E-06	8.03879
5764	PTN	-2.8246	0.83299	-10.4241	1.28E-07	1.81E-06	8.05932
57795	BRINP2	-1.3261	2.91234	-10.4031	1.31E-07	1.85E-06	7.7976
167681	PRSS35	0.65304	6.35185	10.39768	1.32E-07	1.85E-06	7.09279
100093630	SNHG8	1.12817	3.93175	10.39451	1.32E-07	1.86E-06	7.56122
440465	BAIAP2-DT	-0.6082	5.73428	-10.3908	1.33E-07	1.86E-06	7.17495
9185	REPS2	-2.0212	2.05913	-10.3904	1.33E-07	1.86E-06	7.95556
25894	PLEKHG4	-1.139	3.96476	-10.3885	1.33E-07	1.86E-06	7.53788
285237	C3orf38	0.70722	4.98339	10.38704	1.34E-07	1.86E-06	7.31206
646719	NIPBL-DT	-0.9913	3.60368	-10.3835	1.34E-07	1.87E-06	7.60932
6498	SKIL	0.87566	4.7573	10.38026	1.35E-07	1.87E-06	7.35398
6256	RXRA	0.63326	5.89412	10.37587	1.35E-07	1.88E-06	7.13539
730101	LOC730101	-0.9949	3.6894	-10.3701	1.36E-07	1.89E-06	7.57433

81544	GDPD5	0.87843	4.94737	10.36842	1.36E-07	1.89E-06	7.30212
84671	ZNF347	-0.9879	4.55771	-10.3676	1.36E-07	1.89E-06	7.37806
101929523	LOC101929523	-4.5869	-1.363	-10.367	1.37E-07	1.89E-06	7.78337
154664	ABCA13	1.03312	4.54269	10.35615	1.38E-07	1.91E-06	7.37575
590	BCHE	1.40659	2.55829	10.34731	1.40E-07	1.93E-06	7.81439
8559	PRPF18	1.08306	3.75225	10.34132	1.41E-07	1.94E-06	7.53762
85455	DISP2	-0.8255	5.19623	-10.3397	1.41E-07	1.94E-06	7.21362
3782	KCNN3	-3.5821	-0.84402	-10.3383	1.41E-07	1.94E-06	7.75625
27198	HCAR1	1.60851	2.50124	10.33537	1.41E-07	1.94E-06	7.81898
139221	PWWP3B	-3.2945	0.04217	-10.3311	1.42E-07	1.95E-06	7.8776
3628	INPP1	0.98069	3.73201	10.32279	1.43E-07	1.97E-06	7.51618
513	ATP5F1D	-0.8497	4.995	-10.3146	1.45E-07	1.98E-06	7.22298
10314	LANCL1	-0.734	6.53136	-10.3078	1.46E-07	1.99E-06	6.96072
7357	UGCG	0.99197	4.90078	10.30697	1.46E-07	1.99E-06	7.2421
10066	SCAMP2	0.66873	7.09053	10.30633	1.46E-07	1.99E-06	6.89217
8495	PPFIBP2	-4.3424	-1.2364	-10.2999	1.47E-07	2.01E-06	7.71769
144402	CPNE8	1.18119	3.73107	10.29693	1.48E-07	2.01E-06	7.49552
4122	MAN2A2	0.59069	6.44475	10.2945	1.48E-07	2.01E-06	6.95686
83595	SOX7	0.91731	4.41315	10.27926	1.51E-07	2.04E-06	7.30864
2171	FABP5	-0.6412	6.01475	-10.2767	1.51E-07	2.05E-06	6.99583
57597	BAHCC1	-0.6834	5.45827	-10.2743	1.51E-07	2.05E-06	7.0855
80164	PRR36	-1.0793	3.54598	-10.2737	1.52E-07	2.05E-06	7.49693
340371	NRBP2	-1.0042	4.3171	-10.2722	1.52E-07	2.05E-06	7.31729
2043	EPHA4	-3.9481	0.01662	-10.2389	1.58E-07	2.12E-06	7.74619
79754	ASB13	1.05591	3.92552	10.22156	1.61E-07	2.16E-06	7.35529
10157	AASS	-1.5693	2.86058	-10.2198	1.61E-07	2.16E-06	7.60635
134218	DNAJC21	-0.6062	5.57589	-10.2182	1.61E-07	2.16E-06	6.99678
54478	PIMREG	-0.8049	4.69712	-10.2045	1.64E-07	2.19E-06	7.15056
10867	TSPAN9	0.86122	4.1798	10.1981	1.65E-07	2.20E-06	7.26137
84628	NTNG2	-2.525	0.51195	-10.1978	1.65E-07	2.20E-06	7.80387
84189	SLITRK6	1.21145	4.09563	10.19483	1.66E-07	2.21E-06	7.29163
55366	LGR4	-0.8652	5.29395	-10.1856	1.67E-07	2.23E-06	7.01295
57579	FAM135A	-1.092	4.1976	-10.1749	1.69E-07	2.25E-06	7.23142
7374	UNG	0.71064	6.45944	10.16966	1.70E-07	2.26E-06	6.80716
151195	CCNYL1	-0.8162	4.81757	-10.1696	1.70E-07	2.26E-06	7.08462
10800	CYSLTR1	4.59563	-1.0244	10.16921	1.70E-07	2.26E-06	7.60878
134145	ATPSCKMT	-1.0645	3.3624	-10.1655	1.71E-07	2.26E-06	7.41045
3554	IL1R1	-2.3532	2.03868	-10.1643	1.71E-07	2.27E-06	7.71004
8741	TNFSF13	1.91153	1.72562	10.16173	1.72E-07	2.27E-06	7.73345
79937	CNTNAP3	-1.0371	3.62213	-10.1614	1.72E-07	2.27E-06	7.34525
1384	CRAT	0.70868	5.45285	10.15959	1.72E-07	2.27E-06	6.95354
1462	VCAN	3.14942	0.1187	10.14363	1.75E-07	2.31E-06	7.69127
7137	TNNI3	-2.0534	2.33131	-10.1365	1.77E-07	2.32E-06	7.62534
22941	SHANK2	0.93391	3.87953	10.1355	1.77E-07	2.32E-06	7.25819
115207	KCTD12	-0.6599	5.31859	-10.1341	1.77E-07	2.32E-06	6.94261
25789	TMEM59L	-3.415	-0.14135	-10.1327	1.78E-07	2.32E-06	7.63881
4638	MYLK	-0.7577	5.8932	-10.1302	1.78E-07	2.33E-06	6.8412
11174	ADAMTS6	2.02755	1.83726	10.12567	1.79E-07	2.34E-06	7.68402
54662	TBC1D13	0.65112	6.18185	10.1151	1.81E-07	2.36E-06	6.78019
132884	EVC2	2.03909	1.37496	10.11488	1.81E-07	2.36E-06	7.70948

1836	SLC26A2	-0.8438	6.78995	-10.1521	1.83E-07	2.37E-06	6.69829
28988	DBNL	-0.7662	5.74149	-10.1072	1.83E-07	2.37E-06	6.83811
991	CDC20	-0.7853	6.65107	-10.0999	1.84E-07	2.38E-06	6.69645
157855	KCNU1	2.74741	0.91777	10.07897	1.89E-07	2.43E-06	7.68376
3434	IFIT1	0.8051	4.89795	10.07694	1.89E-07	2.44E-06	6.96196
91752	ZNF804A	-1.187	3.58294	-10.0686	1.91E-07	2.46E-06	7.24981
3764	KCNJ8	-1.8931	1.56364	-10.0565	1.94E-07	2.49E-06	7.6233
116349	EXOC3-AS1	-1.6402	2.12222	-10.0426	1.97E-07	2.52E-06	7.5338
2044	EPHA5	-0.9984	5.0643	-10.038	1.98E-07	2.53E-06	6.88225
1825	DSC3	-0.8539	7.10126	-10.1997	1.99E-07	2.55E-06	6.59183
5147	PDE6D	-0.6757	5.03007	-10.0287	2.00E-07	2.55E-06	6.87021
151194	METTL21A	-0.8774	4.00736	-10.0224	2.01E-07	2.57E-06	7.08382
204962	SLC44A5	-3.8344	-0.47447	-10.0207	2.02E-07	2.57E-06	7.46076
64979	MRPL36	-0.6705	5.14102	-10.0118	2.04E-07	2.59E-06	6.8283
54906	TASOR2	0.9109	6.24651	10.045	2.07E-07	2.62E-06	6.64456
27345	KCNMB4	-1.7898	1.64533	-9.99991	2.07E-07	2.62E-06	7.54821
8425	LTBP4	-0.5889	5.95007	-9.99344	2.08E-07	2.64E-06	6.66401
8792	TNFRSF11A	-0.6246	5.33671	-9.97925	2.12E-07	2.67E-06	6.75144
83442	SH3BGRL3	-0.679	9.33223	-10.0756	2.12E-07	2.67E-06	6.32927
794	CALB2	3.65491	-1.20249	9.973767	2.13E-07	2.68E-06	7.37807
4839	NOP2	0.60096	5.75334	9.967461	2.14E-07	2.69E-06	6.66728
54461	FBXW5	0.8922	6.13441	9.990106	2.15E-07	2.70E-06	6.61516
29886	SNX8	0.65167	5.60977	9.961281	2.16E-07	2.71E-06	6.68487
56623	INPP5E	0.84982	4.79594	9.957158	2.17E-07	2.72E-06	6.83907
401474	SAMD12	0.64232	5.87342	9.942491	2.21E-07	2.76E-06	6.61811
7410	VAV2	0.6688	5.91746	9.93079	2.24E-07	2.79E-06	6.59738
157313	CDCA2	-0.612	5.93468	-9.92375	2.25E-07	2.81E-06	6.58161
169714	QSOX2	0.78362	4.99216	9.922749	2.26E-07	2.81E-06	6.75563
716	C1S	0.74355	5.04833	9.919147	2.27E-07	2.82E-06	6.73905
83860	TAF3	1.09382	4.106	9.91185	2.29E-07	2.84E-06	6.943
5737	PTGFR	-0.7254	5.63769	-9.91157	2.29E-07	2.84E-06	6.61704
64225	ATL2	-0.6943	5.71362	-9.89533	2.33E-07	2.89E-06	6.58389
9750	RIPOR2	-2.8764	1.59041	-9.89439	2.33E-07	2.89E-06	7.45439
692099	FAM86DP	0.91857	4.03236	9.892224	2.34E-07	2.89E-06	6.92893
196527	ANO6	0.66784	6.6196	9.888251	2.35E-07	2.91E-06	6.44341
81832	NETO1	-3.6479	-0.10122	-9.88573	2.36E-07	2.91E-06	7.37458
4653	MYOC	-2.4103	1.41191	-9.87436	2.39E-07	2.94E-06	7.43383
3358	HTR2C	-5.0166	-0.61832	-9.87092	2.40E-07	2.95E-06	7.32104
100505876	CEBPZOS	-0.589	6.18281	-9.86764	2.41E-07	2.96E-06	6.47515
1186	CLCN7	0.91456	4.38012	9.865359	2.41E-07	2.96E-06	6.81743
285148	IAH1	-0.6888	4.92852	-9.86105	2.42E-07	2.97E-06	6.68589
730102	CRYZL2P	-1.8521	1.94079	-9.84364	2.47E-07	3.02E-06	7.33159
8671	SLC4A4	-1.713	2.88204	-9.83713	2.49E-07	3.04E-06	7.15501
113675	SDSL	1.22439	3.774	9.835373	2.50E-07	3.05E-06	6.93433
93010	B3GNT7	-3.6691	-0.24451	-9.82996	2.51E-07	3.06E-06	7.29187
100506994	PTPRG-AS1	3.30262	0.20281	9.827469	2.52E-07	3.06E-06	7.35631
196	AHR	-0.7274	5.97677	-9.82699	2.52E-07	3.06E-06	6.45814
25777	SUN2	-0.6318	6.04289	-9.82598	2.52E-07	3.06E-06	6.44512
23324	MAN2B2	0.96441	4.32595	9.825394	2.53E-07	3.06E-06	6.78216
90355	MACIR	0.6548	5.6364	9.806352	2.58E-07	3.12E-06	6.49033

23371	TNS2	0.64444	5.14682	9.803072	2.59E-07	3.13E-06	6.57507
59341	TRPV4	1.18125	3.34641	9.775208	2.68E-07	3.22E-06	6.95791
83734	ATG10	-0.9523	3.56857	-9.76193	2.72E-07	3.27E-06	6.8717
158038	LINGO2	-1.5535	2.15784	-9.75879	2.73E-07	3.28E-06	7.18932
23301	EHBP1	-0.6654	6.08391	-9.75262	2.75E-07	3.30E-06	6.34874
3983	ABLIM1	-0.6855	5.49288	-9.74898	2.76E-07	3.31E-06	6.44132
4354	MPP1	0.71765	4.70692	9.737161	2.80E-07	3.34E-06	6.58343
10991	SLC38A3	-4.7748	-1.79108	-9.73609	2.80E-07	3.34E-06	7.12571
83540	NUF2	-0.7009	5.30539	-9.7348	2.81E-07	3.34E-06	6.45815
646600	IGF2BP2-AS1	1.56089	2.75429	9.733692	2.81E-07	3.34E-06	7.05751
51651	PTRH2	0.65863	5.7904	9.733202	2.81E-07	3.34E-06	6.37409
84665	MYPN	0.9067	3.68791	9.724143	2.84E-07	3.37E-06	6.80308
101927111	SUCLG2-AS1	2.01837	1.70075	9.705497	2.90E-07	3.44E-06	7.20674
3241	HPCAL1	-0.6505	7.92843	-9.70115	2.92E-07	3.45E-06	6.07081
64794	DDX31	0.73227	4.51031	9.69564	2.94E-07	3.47E-06	6.57384
6309	SC5D	-0.6201	6.11258	-9.68158	2.99E-07	3.52E-06	6.25546
55051	NRDE2	1.21556	3.65749	9.672519	3.02E-07	3.56E-06	6.76109
4688	NCF2	1.62907	2.03206	9.666227	3.04E-07	3.58E-06	7.10637
158586	ZXDB	0.80917	4.7032	9.660288	3.06E-07	3.60E-06	6.49122
79180	EFHD2	0.66913	6.89814	9.655236	3.08E-07	3.61E-06	6.11997
3017	H2BC5	0.83671	4.62157	9.65132	3.10E-07	3.63E-06	6.49813
728855	LINC00623	-4.6576	-1.32078	-9.64533	3.12E-07	3.65E-06	7.05065
3887	KRT81	0.9881	9.27589	10.53535	3.14E-07	3.67E-06	6.06457
5359	PLSCR1	0.67446	5.45756	9.638021	3.14E-07	3.67E-06	6.31285
92906	HNRNPLL	-0.7397	5.27977	-9.63354	3.16E-07	3.68E-06	6.33745
23508	TTC9	-2.4314	0.84238	-9.62919	3.18E-07	3.70E-06	7.17273
1847	DUSP5	0.62197	7.19299	9.626058	3.19E-07	3.71E-06	6.04887
1848	DUSP6	0.60472	6.95715	9.619263	3.21E-07	3.74E-06	6.06707
901	CCNG2	-0.8955	5.27737	-9.6178	3.22E-07	3.74E-06	6.32144
89795	NAV3	1.3868	3.46183	9.617676	3.22E-07	3.74E-06	6.74707
656	BMP8B	-2.7821	0.64677	-9.61416	3.23E-07	3.75E-06	7.15515
55084	SOBP	-2.4006	1.53479	-9.61341	3.24E-07	3.75E-06	7.11838
4674	NAP1L2	-0.9643	4.07694	-9.61153	3.24E-07	3.75E-06	6.56715
54492	NEURL1B	-1.2988	2.76091	-9.60542	3.27E-07	3.78E-06	6.87756
85027	SMIM3	-0.585	5.60134	-9.59863	3.29E-07	3.79E-06	6.23323
221393	ADGRF4	2.09243	1.44761	9.587152	3.34E-07	3.84E-06	7.09134
23424	TDRD7	0.80046	4.12456	9.586216	3.34E-07	3.84E-06	6.52525
3866	KRT15	-0.9925	3.58235	-9.57348	3.39E-07	3.89E-06	6.63694
79905	TMC7	-0.7519	4.74458	-9.57269	3.40E-07	3.89E-06	6.36687
56683	CFAP298	0.58046	5.59869	9.572362	3.40E-07	3.89E-06	6.20367
55054	ATG16L1	-0.6037	6.23806	-9.56862	3.41E-07	3.90E-06	6.09528
9227	LRAT	-3.3743	-0.32001	-9.55647	3.46E-07	3.94E-06	6.99968
81846	SBF2	-0.6646	5.92589	-9.55264	3.48E-07	3.96E-06	6.12296
100506289	LOC100506289	-3.6585	-1.05344	-9.53471	3.55E-07	4.04E-06	6.92176
80213	TM2D3	0.75653	4.35482	9.524081	3.60E-07	4.08E-06	6.39411
9479	MAPK8IP1	-0.8073	4.74711	-9.52228	3.61E-07	4.09E-06	6.30409
5063	PAK3	-4.8099	-0.59641	-9.52141	3.61E-07	4.09E-06	6.94541
389336	C5orf46	-0.9474	3.80403	-9.51873	3.62E-07	4.10E-06	6.5143
79071	ELOVL6	-0.8021	4.89003	-9.50367	3.69E-07	4.17E-06	6.25122
84561	SLC12A8	4.04718	-0.38697	9.50118	3.70E-07	4.17E-06	6.92129

4751	NEK2	-0.7593	5.13817	-9.47953	3.79E-07	4.27E-06	6.17085
23148	NACAD	-2.5622	1.2901	-9.47923	3.80E-07	4.27E-06	6.97908
2870	GRK6	-0.7322	5.57472	-9.47734	3.80E-07	4.27E-06	6.08714
5314	PKHD1	-1.8295	2.33267	-9.47547	3.81E-07	4.28E-06	6.82555
259266	ASPM	-0.8624	5.69501	-9.46386	3.87E-07	4.33E-06	6.05211
3749	KCNC4	-3.0712	0.38637	-9.45395	3.91E-07	4.38E-06	6.96045
55753	OGDHL	1.16844	3.17521	9.446813	3.95E-07	4.41E-06	6.58972
246243	RNASEH1	-0.679	4.95798	-9.44501	3.95E-07	4.41E-06	6.16091
10457	GPNMB	3.9708	-1.57372	9.437757	3.99E-07	4.44E-06	6.8046
5825	ABCD3	-0.6074	5.74564	-9.43196	4.02E-07	4.46E-06	5.99835
51537	MTFP1	-0.7219	4.63177	-9.43131	4.02E-07	4.46E-06	6.21123
81930	KIF18A	-0.7359	4.56858	-9.406	4.14E-07	4.59E-06	6.19286
284021	MILR1	1.15558	4.10657	9.394189	4.20E-07	4.64E-06	6.30319
4037	LRP3	0.89706	4.96192	9.390989	4.22E-07	4.66E-06	6.10194
116843	SLC18B1	-0.8856	3.96416	-9.38215	4.26E-07	4.70E-06	6.30155
6509	SLC1A4	-0.6135	5.64457	-9.3702	4.32E-07	4.76E-06	5.93685
219844	HYLS1	-0.654	5.21984	-9.36609	4.35E-07	4.78E-06	6.00912
23096	IQSEC2	0.60169	5.48761	9.364771	4.35E-07	4.78E-06	5.96041
51390	AIG1	-0.6471	5.73458	-9.35795	4.39E-07	4.81E-06	5.90644
57484	RNF150	-0.9974	4.20533	-9.35652	4.40E-07	4.82E-06	6.21748
10295	BCKDK	-0.6884	5.88569	-9.35182	4.42E-07	4.84E-06	5.87449
5934	RBL2	-0.6295	5.87798	-9.34672	4.45E-07	4.86E-06	5.86825
203	AK1	0.63452	5.96607	9.341746	4.48E-07	4.88E-06	5.85138
282969	FUOM	-1.8055	1.44699	-9.33468	4.51E-07	4.91E-06	6.77711
7884	SLBP	0.60109	5.59535	9.322236	4.58E-07	4.98E-06	5.88698
5205	ATP8B1	0.79213	4.7879	9.313894	4.63E-07	5.02E-06	6.03554
27042	UTP25	0.74251	4.75076	9.306214	4.67E-07	5.05E-06	6.03206
90441	ZNF622	-0.6108	5.57156	-9.30447	4.68E-07	5.06E-06	5.86518
91373	UAP1L1	0.82624	4.46155	9.297749	4.72E-07	5.09E-06	6.08549
3140	MR1	1.23131	2.97845	9.297274	4.72E-07	5.09E-06	6.45027
116984	ARAP2	1.64528	1.71184	9.294422	4.74E-07	5.11E-06	6.69701
6857	SYT1	0.80387	4.1889	9.291573	4.76E-07	5.12E-06	6.13797
90293	KLHL13	-0.8018	4.66409	-9.28975	4.77E-07	5.13E-06	6.0258
7286	TUFT1	0.8292	4.36353	9.276197	4.85E-07	5.20E-06	6.07972
57658	CALCOCO1	0.63258	4.77448	9.274802	4.85E-07	5.21E-06	5.98392
85363	TRIM5	0.63672	5.50284	9.271078	4.88E-07	5.22E-06	5.83819
11067	DEPP1	-1.0829	3.25491	-9.27037	4.88E-07	5.22E-06	6.33622
79102	RNF26	-0.605	7.48579	-9.26789	4.89E-07	5.23E-06	5.56016
51454	GULP1	0.60158	7.29196	9.262849	4.92E-07	5.26E-06	5.57536
130814	SLC66A3	-1.1655	3.15748	-9.26194	4.93E-07	5.26E-06	6.35131
10451	VAV3	-3.6982	-0.15732	-9.26054	4.94E-07	5.27E-06	6.68026
4288	MKI67	-0.621	8.5256	-9.25987	4.94E-07	5.27E-06	5.46382
23046	KIF21B	-3.4896	-0.64162	-9.25889	4.95E-07	5.27E-06	6.6351
4915	NTRK2	-3.9843	0.18885	-9.25678	4.96E-07	5.28E-06	6.70113
55636	CHD7	-2.7629	0.64298	-9.25343	4.98E-07	5.29E-06	6.7333
3732	CD82	-1.4948	2.21187	-9.25301	4.98E-07	5.29E-06	6.55722
7620	ZNF69	-3.0027	0.8373	-9.25267	4.99E-07	5.29E-06	6.73142
5332	PLCB4	0.65226	5.78146	9.230611	5.12E-07	5.43E-06	5.73846
6595	SMARCA2	0.58244	6.16963	9.223018	5.17E-07	5.47E-06	5.66609
4584	MUC3A	-0.9505	4.41277	-9.22218	5.17E-07	5.47E-06	5.9968

10783	NEK6	0.58477	5.6419	9.219494	5.19E-07	5.48E-06	5.74624
375484	SIMC1	-0.7003	4.35768	-9.20855	5.26E-07	5.54E-06	5.9848
221830	POLR1F	0.58709	5.66294	9.19831	5.33E-07	5.60E-06	5.71521
84909	AOPEP	1.19814	2.77406	9.197559	5.33E-07	5.60E-06	6.36885
54812	AFTPH	-0.6128	5.73623	-9.1901	5.38E-07	5.64E-06	5.6893
59348	ZNF350	1.51907	2.27889	9.184929	5.41E-07	5.67E-06	6.46751
284013	VMO1	3.40018	-0.51959	9.18395	5.42E-07	5.67E-06	6.55736
5591	PRKDC	-0.6675	7.43607	-9.17594	5.47E-07	5.72E-06	5.44643
4772	NFATC1	-1.3777	2.20848	-9.17279	5.50E-07	5.73E-06	6.45271
6620	SNCB	-3.0611	0.0583	-9.16649	5.54E-07	5.77E-06	6.60916
29899	GPSM2	-0.6984	4.95757	-9.16598	5.54E-07	5.77E-06	5.80333
23567	ZNF346	-0.6724	5.17684	-9.16389	5.56E-07	5.78E-06	5.75728
24146	CLDN15	0.90492	3.56612	9.148354	5.66E-07	5.88E-06	6.10611
169611	OLFML2A	-2.302	2.05564	-9.13417	5.76E-07	5.98E-06	6.4654
374659	HDDC3	0.89834	4.51619	9.131275	5.78E-07	5.99E-06	5.86149
4644	MYO5A	-0.9099	7.10981	-9.79101	5.79E-07	6.00E-06	5.55716
338761	C1QL4	-1.9531	1.67254	-9.12865	5.80E-07	6.00E-06	6.49922
1054	CEBPG	0.60631	5.59206	9.126204	5.82E-07	6.01E-06	5.63411
123720	WHAMM	0.74977	4.54762	9.119009	5.87E-07	6.06E-06	5.83379
217	ALDH2	-0.8961	3.77433	-9.11526	5.90E-07	6.07E-06	6.00492
3156	HMGCR	-0.5856	6.0637	-9.11509	5.90E-07	6.07E-06	5.53914
55071	C9orf40	1.24093	3.33743	9.11346	5.91E-07	6.07E-06	6.13129
23642	SNHG1	0.63881	5.44647	9.110361	5.93E-07	6.09E-06	5.63996
57526	PCDH19	-2.5198	1.04878	-9.10161	6.00E-07	6.13E-06	6.53641
79883	PODNL1	1.60361	1.98231	9.099258	6.01E-07	6.14E-06	6.4128
6801	STRN	-0.9198	5.5484	-9.12593	6.06E-07	6.18E-06	5.60822
51106	TFB1M	-0.8012	4.40594	-9.09259	6.06E-07	6.18E-06	5.82618
1841	DTYMK	-0.7517	5.84538	-9.08652	6.11E-07	6.21E-06	5.53858
2992	GYG1	0.61743	5.22857	9.07756	6.18E-07	6.27E-06	5.63745
5142	PDE4B	3.25727	-0.7531	9.075918	6.19E-07	6.27E-06	6.42572
23015	GOLGA8A	-1.061	3.05245	-9.07525	6.19E-07	6.27E-06	6.1332
3321	IGSF3	-2.4895	0.37956	-9.07159	6.22E-07	6.29E-06	6.5149
2048	EPHB2	-0.6804	4.66952	-9.07063	6.23E-07	6.30E-06	5.73802
51701	NLK	0.70875	4.47502	9.068599	6.25E-07	6.31E-06	5.78252
157922	CAMSAP1	0.6161	5.90338	9.062568	6.29E-07	6.34E-06	5.499
2250	FGF5	0.89837	4.00466	9.061176	6.30E-07	6.34E-06	5.88722
114818	KLHL29	-4.2865	-1.26322	-9.05477	6.35E-07	6.39E-06	6.39864
10723	SLC12A7	-1.3081	2.26738	-9.05111	6.38E-07	6.41E-06	6.28405
9214	FCMR	2.4946	0.22381	9.049951	6.39E-07	6.41E-06	6.4846
115201	ATG4A	0.78283	4.12634	9.042785	6.45E-07	6.46E-06	5.82981
54509	RHOF	0.60472	7.45379	9.038844	6.48E-07	6.49E-06	5.26648
8706	B3GALNT1	-0.6548	7.10173	-9.03631	6.50E-07	6.49E-06	5.29952
201255	LRRC45	-0.6431	5.44499	-9.02154	6.62E-07	6.61E-06	5.52045
283431	GAS2L3	-0.6511	5.65856	-9.01245	6.69E-07	6.67E-06	5.47096
9630	GNA14	2.08656	1.26236	9.012249	6.70E-07	6.67E-06	6.4062
22915	MMRN1	-1.8464	1.89005	-9.00758	6.74E-07	6.69E-06	6.31258
11153	FICD	0.64267	4.82882	8.997126	6.82E-07	6.77E-06	5.61193
10561	IFI44	0.90772	3.90866	8.993437	6.85E-07	6.80E-06	5.82209
100506686	IQCH-AS1	1.82047	2.18134	8.987578	6.90E-07	6.84E-06	6.24593
9759	HDAC4	-0.6962	4.49238	-8.98747	6.91E-07	6.84E-06	5.66741

347735	SERINC2	-0.7427	4.42928	-8.97533	7.01E-07	6.92E-06	5.66611
84240	ZCCHC9	-0.6635	4.47322	-8.97356	7.03E-07	6.93E-06	5.65247
57481	KIAA1210	-3.5578	-0.2934	-8.97281	7.03E-07	6.93E-06	6.34123
9354	UBE4A	-0.6036	7.10764	-8.96139	7.13E-07	7.00E-06	5.19947
57504	MTA3	-0.5822	5.26081	-8.959	7.15E-07	7.02E-06	5.47148
79814	AGMAT	3.53081	0.76781	8.956793	7.17E-07	7.03E-06	6.37653
55322	C5orf22	-0.6441	5.39873	-8.95537	7.19E-07	7.04E-06	5.4419
218	ALDH3A1	0.78309	5.90412	8.953763	7.20E-07	7.04E-06	5.35926
54972	TMEM132A	-0.7194	5.68337	-8.94799	7.25E-07	7.08E-06	5.38273
56106	PCDHGA10	-3.651	0.03283	-8.94644	7.27E-07	7.08E-06	6.33712
6683	SPAST	-0.728	4.79474	-8.94333	7.30E-07	7.10E-06	5.54569
23162	MAPK8IP3	0.6195	4.96752	8.93937	7.33E-07	7.13E-06	5.50692
685	BTC	-0.9489	3.51902	-8.93327	7.39E-07	7.17E-06	5.8312
8555	CDC14B	0.71723	4.70824	8.923512	7.48E-07	7.23E-06	5.54192
26575	RGS17	-1.2316	3.60488	-8.91701	7.54E-07	7.28E-06	5.80174
9456	HOMER1	-0.8987	4.32839	-8.90592	7.64E-07	7.37E-06	5.60152
1385	CREB1	-0.815	5.5451	-8.90447	7.66E-07	7.38E-06	5.35105
8507	ENC1	-0.6557	5.41708	-8.89691	7.73E-07	7.43E-06	5.36125
25840	METTL7A	4.28518	-1.01417	8.885733	7.84E-07	7.52E-06	6.21654
23491	CES3	1.50391	2.16796	8.880692	7.89E-07	7.56E-06	6.09769
27063	ANKRD1	3.00545	0.04643	8.879597	7.90E-07	7.57E-06	6.2675
599	BCL2L2	-0.582	5.06967	-8.87213	7.97E-07	7.63E-06	5.39318
1021	CDK6	0.69462	5.79789	8.866395	8.03E-07	7.68E-06	5.25856
112476	PRRT2	-2.013	0.81587	-8.86159	8.08E-07	7.72E-06	6.24568
374	AREG	-0.645	5.74344	-8.86013	8.10E-07	7.72E-06	5.25459
4100	MAGEA1	-5.1868	-0.96429	-8.98781	8.13E-07	7.74E-06	6.19899
10418	SPON1	-4.2627	-1.80436	-8.8542	8.16E-07	7.76E-06	6.14818
257407	C2orf72	-2.2982	0.56548	-8.85226	8.18E-07	7.77E-06	6.24617
5163	PDK1	-0.7058	5.31692	-8.84002	8.30E-07	7.87E-06	5.30506
378938	MALAT1	0.73424	6.5084	8.830529	8.40E-07	7.95E-06	5.10331
23586	DDX58	0.7485	4.66359	8.827601	8.43E-07	7.97E-06	5.42491
9187	SLC24A1	0.94606	4.19358	8.824209	8.47E-07	8.00E-06	5.53227
9855	FARP2	-0.8758	4.17877	-8.81129	8.61E-07	8.10E-06	5.5095
55601	DDX60	1.40099	4.44614	9.33171	8.64E-07	8.12E-06	5.58219
6273	S100A2	-0.6994	4.66301	-8.8051	8.68E-07	8.14E-06	5.38909
645996	NAP1L6P	-2.2562	0.65534	-8.80317	8.70E-07	8.15E-06	6.18218
286499	FAM133A	2.46885	0.48679	8.796865	8.77E-07	8.20E-06	6.18031
5833	PCYT2	-0.679	4.70666	-8.79668	8.77E-07	8.20E-06	5.36808
10459	MAD2L2	-0.6004	5.4849	-8.78509	8.90E-07	8.31E-06	5.19884
11166	SOX21	-1.2695	2.49655	-8.77042	9.06E-07	8.44E-06	5.87162
285636	C5orf51	-0.5848	5.84288	-8.76303	9.15E-07	8.51E-06	5.10743
26289	AK5	-0.8037	3.92626	-8.76194	9.16E-07	8.51E-06	5.50047
2961	GTF2E2	-0.5828	5.70009	-8.7523	9.27E-07	8.60E-06	5.11679
85465	SELENOI	-0.6249	5.95927	-8.74466	9.36E-07	8.67E-06	5.06475
3775	KCNK1	-0.6003	4.90093	-8.73362	9.50E-07	8.77E-06	5.24197
23288	IQCE	0.67348	5.30234	8.728724	9.56E-07	8.81E-06	5.16164
29799	YPEL1	-0.8667	3.71066	-8.7276	9.57E-07	8.82E-06	5.50914
54982	CLN6	0.70722	5.72785	8.723699	9.62E-07	8.84E-06	5.07949
151507	MSL3P1	-0.7745	4.46379	-8.72231	9.63E-07	8.84E-06	5.32303
8934	RAB29	0.60325	4.78329	8.722104	9.64E-07	8.84E-06	5.25467

5933	RBL1	-0.59	6.28237	-8.7209	9.65E-07	8.85E-06	4.98389
23223	RRP12	0.66736	5.62448	8.72086	9.65E-07	8.85E-06	5.09245
114787	GPRIN1	-0.6111	5.29519	-8.71723	9.70E-07	8.88E-06	5.14281
55364	IMPACT	-0.7438	5.24863	-8.70839	9.81E-07	8.96E-06	5.14217
22954	TRIM32	0.59387	5.2641	8.704859	9.85E-07	9.00E-06	5.13506
7091	TLE4	0.72925	4.20057	8.70199	9.89E-07	9.02E-06	5.35913
11005	SPINK5	-1.8403	1.94133	-8.69197	1.00E-06	9.12E-06	5.89712
51754	TMEM8B	-0.7614	4.41646	-8.69147	1.00E-06	9.12E-06	5.29159
2258	FGF13	-2.8868	0.37031	-8.68994	1.00E-06	9.13E-06	6.04841
22920	KIFAP3	-0.7488	4.20314	-8.68584	1.01E-06	9.17E-06	5.33185
10391	CORO2B	-2.8011	-0.41466	-8.67931	1.02E-06	9.23E-06	6.00206
9245	GCNT3	1.91537	1.67501	8.659042	1.04E-06	9.44E-06	5.9036
347902	AMIGO2	-1.0339	4.09148	-8.65242	1.05E-06	9.51E-06	5.32207
254427	PROSER2	1.2716	2.88265	8.649618	1.06E-06	9.54E-06	5.63284
56143	PCDHA5	3.4378	-0.38598	8.647281	1.06E-06	9.56E-06	5.94706
2537	IFI6	1.68115	4.65687	9.355943	1.07E-06	9.64E-06	5.36525
80004	ESRP2	0.748	4.77021	8.639335	1.07E-06	9.64E-06	5.14948
8320	EOMES	-2.6371	0.77143	-8.61789	1.10E-06	9.88E-06	5.94761
22981	NINL	-1.0507	3.11902	-8.61087	1.11E-06	9.96E-06	5.50406
51704	GPRC5B	-0.6165	5.19787	-8.60686	1.12E-06	1.00E-05	5.01213
1295	COL8A1	1.34943	2.7074	8.59455	1.13E-06	1.01E-05	5.60292
221937	FOXK1	0.63034	5.4229	8.593634	1.14E-06	1.01E-05	4.95535
2176	FANCC	0.6107	4.92731	8.592485	1.14E-06	1.02E-05	5.04986
29070	CCDC113	-0.7928	4.58575	-8.58038	1.15E-06	1.03E-05	5.10494
5128	CDK17	0.63005	4.96166	8.566439	1.18E-06	1.05E-05	5.00771
64122	FN3K	-0.705	4.33083	-8.56086	1.18E-06	1.05E-05	5.13248
79925	SPEF2	-0.9943	3.10405	-8.55835	1.19E-06	1.06E-05	5.43547
152687	ZNF595	1.88358	1.38349	8.55318	1.20E-06	1.06E-05	5.80189
6297	SALL2	-0.618	4.76483	-8.55145	1.20E-06	1.06E-05	5.02367
64324	NSD1	-0.6421	7.34477	-8.5432	1.21E-06	1.07E-05	4.61077
22901	ARSG	1.38349	2.50891	8.541648	1.21E-06	1.07E-05	5.57827
9254	CACNA2D2	-0.8739	3.82313	-8.54056	1.22E-06	1.07E-05	5.22931
8563	THOC5	-0.6343	5.10079	-8.53992	1.22E-06	1.07E-05	4.94013
3601	IL15RA	1.52395	1.63545	8.536523	1.22E-06	1.08E-05	5.73666
3576	CXCL8	1.2443	2.49809	8.534022	1.23E-06	1.08E-05	5.56427
440044	SLC22A20P	2.20134	0.43731	8.521804	1.25E-06	1.09E-05	5.8353
27074	LAMP3	0.69655	4.63152	8.518859	1.25E-06	1.10E-05	5.01365
57168	ASPHD2	-3.2228	-1.02556	-8.51567	1.26E-06	1.10E-05	5.7628
5010	CLDN11	0.99327	4.71467	8.514919	1.26E-06	1.10E-05	5.00019
8890	EIF2B4	-0.6077	5.24099	-8.5108	1.26E-06	1.11E-05	4.87264
152519	NIPAL1	1.13576	2.6194	8.507292	1.27E-06	1.11E-05	5.49637
2167	FABP4	-2.3876	2.83374	-9.07627	1.27E-06	1.11E-05	5.61397
5251	PHEX	1.39366	2.90912	8.503142	1.28E-06	1.11E-05	5.43655
90957	DHX57	-0.6173	5.11809	-8.50167	1.28E-06	1.12E-05	4.88404
652968	CASTOR1	-1.1384	2.5696	-8.49874	1.28E-06	1.12E-05	5.48857
84803	GPAT3	-0.6838	4.94472	-8.49644	1.29E-06	1.12E-05	4.91262
10079	ATP9A	-0.5954	8.52839	-8.52718	1.29E-06	1.12E-05	4.45229
54956	PARP16	0.6248	4.75837	8.488458	1.30E-06	1.13E-05	4.94318
112597	CYTOR	1.3078	3.11806	8.4846	1.31E-06	1.14E-05	5.35506
84983	FAM222A-AS1	2.36902	0.22712	8.459731	1.35E-06	1.17E-05	5.75979

119391	GSTO2	1.54338	1.9037	8.45903	1.35E-06	1.17E-05	5.59124
5820	PVT1	0.77218	4.04788	8.449957	1.37E-06	1.18E-05	5.05418
100288152	SLC9A3-AS1	-1.0135	3.59916	-8.44839	1.37E-06	1.19E-05	5.16399
389337	ARHGEF37	-0.6404	5.49069	-8.43831	1.39E-06	1.20E-05	4.72686
949	SCARB1	0.6552	6.53483	8.432625	1.40E-06	1.21E-05	4.55784
1993	ELAVL2	0.76515	4.00539	8.431991	1.40E-06	1.21E-05	5.0398
10518	CIB2	-0.8909	3.61347	-8.42541	1.41E-06	1.22E-05	5.12462
25920	NELFB	0.62787	6.45468	8.420419	1.42E-06	1.22E-05	4.55155
283316	CD163L1	2.9806	-1.04827	8.40642	1.45E-06	1.24E-05	5.63055
10344	CCL26	1.66407	1.96418	8.405301	1.45E-06	1.24E-05	5.51313
4240	MFGE8	-0.754	5.67123	-8.38261	1.49E-06	1.27E-05	4.61979
9096	TBX18	3.70206	-0.66199	8.378704	1.50E-06	1.28E-05	5.61558
571	BACH1	0.81421	5.10002	8.36962	1.52E-06	1.29E-05	4.71398
4311	MME	0.86779	3.93081	8.362873	1.53E-06	1.30E-05	4.96681
23177	CEP68	-0.8139	4.61426	-8.35846	1.54E-06	1.31E-05	4.79494
2115	ETV1	-0.7963	5.19833	-8.35266	1.55E-06	1.32E-05	4.66631
133686	NADK2	-0.7185	4.62345	-8.3398	1.58E-06	1.34E-05	4.76461
122622	ADSS1	-1.5567	1.70857	-8.33426	1.59E-06	1.34E-05	5.45104
8722	CTSF	0.75499	4.76706	8.327393	1.60E-06	1.36E-05	4.7223
4087	SMAD2	-0.5994	5.21787	-8.31775	1.62E-06	1.37E-05	4.61024
168374	ZNF92	0.63761	4.51223	8.309155	1.64E-06	1.38E-05	4.74877
3659	IRF1	0.9123	3.99881	8.304059	1.65E-06	1.39E-05	4.87089
57082	KNL1	-0.6692	6.70392	-8.2941	1.68E-06	1.40E-05	4.34131
255187	SH3TC2-DT	-1.41	2.39874	-8.28537	1.70E-06	1.41E-05	5.24812
11221	DUSP10	-1.8346	1.17441	-8.28212	1.70E-06	1.42E-05	5.46322
79834	PEAK1	0.79759	5.82247	8.302685	1.72E-06	1.43E-05	4.45673
54885	TBC1D8B	-0.8909	4.69681	-8.26802	1.73E-06	1.44E-05	4.65323
85459	CEP295	-0.7602	5.54588	-8.26704	1.74E-06	1.44E-05	4.48106
199990	FAAP20	-0.7662	5.60765	-8.26621	1.74E-06	1.44E-05	4.4689
10982	MAPRE2	-0.6841	4.51823	-8.26297	1.75E-06	1.45E-05	4.67961
10642	IGF2BP1	1.3782	2.21632	8.254886	1.76E-06	1.46E-05	5.25037
541471	MIR4435-2HG	0.9137	3.82047	8.254607	1.77E-06	1.46E-05	4.84584
54930	HAUS4	-0.831	3.96474	-8.25408	1.77E-06	1.46E-05	4.79861
51608	GET4	0.6661	5.6434	8.25065	1.77E-06	1.46E-05	4.44284
6988	TCTA	0.58355	4.88692	8.248678	1.78E-06	1.47E-05	4.58303
7368	UGT8	0.60578	5.25289	8.247428	1.78E-06	1.47E-05	4.50879
4724	NDUFS4	-0.7764	4.27889	-8.24526	1.79E-06	1.47E-05	4.7108
58488	PCTP	0.60742	4.55666	8.243921	1.79E-06	1.47E-05	4.64723
10681	GNB5	-0.6775	4.6578	-8.23924	1.80E-06	1.48E-05	4.61612
7465	WEE1	-0.6255	4.98967	-8.23733	1.81E-06	1.48E-05	4.54313
3754	KCNF1	-1.9634	1.46353	-8.23591	1.81E-06	1.48E-05	5.36712
79807	GSTCD	-0.6706	4.71337	-8.22854	1.83E-06	1.49E-05	4.58895
348801	LNP1	0.66263	4.48252	8.223761	1.84E-06	1.50E-05	4.63689
23122	CLASP2	-0.6698	6.01567	-8.2237	1.84E-06	1.50E-05	4.34011
8644	AKR1C3	-2.3591	0.74981	-8.22222	1.84E-06	1.50E-05	5.43193
7690	ZNF131	-0.5865	5.36592	-8.22191	1.84E-06	1.50E-05	4.44785
10514	MYBBP1A	0.59948	6.02891	8.22162	1.84E-06	1.50E-05	4.33699
84191	CIAO2A	0.61771	4.9489	8.217637	1.85E-06	1.51E-05	4.52767
8633	UNC5C	-1.5471	1.89304	-8.21302	1.87E-06	1.52E-05	5.25411
83604	TMEM47	-1.3827	2.22821	-8.2101	1.87E-06	1.52E-05	5.17883

9928	KIF14	-0.6347	6.12229	-8.20529	1.88E-06	1.53E-05	4.29734
10461	MERTK	-0.8578	3.73034	-8.20252	1.89E-06	1.54E-05	4.78542
7111	TMOD1	2.43821	0.7563	8.20137	1.89E-06	1.54E-05	5.40826
137886	UBXN2B	-0.6248	4.99554	-8.19821	1.90E-06	1.54E-05	4.48717
402483	LINC01000	0.70949	4.28868	8.194001	1.91E-06	1.55E-05	4.64035
153642	ARSK	-0.8017	4.30503	-8.19099	1.92E-06	1.55E-05	4.6296
57732	ZFYVE28	0.85242	3.59921	8.187033	1.93E-06	1.56E-05	4.80401
56127	PCDHB9	2.31815	0.05513	8.170291	1.97E-06	1.59E-05	5.38939
81796	SLCO5A1	-1.8306	0.90063	-8.15294	2.02E-06	1.62E-05	5.31886
8558	CDK10	0.78359	5.09317	8.150789	2.03E-06	1.62E-05	4.40848
84895	MIGA2	0.62831	4.60159	8.146438	2.04E-06	1.63E-05	4.50101
64780	MICAL1	-0.7289	4.0485	-8.14106	2.05E-06	1.64E-05	4.61717
6785	ELOVL4	-0.9845	3.04888	-8.14064	2.05E-06	1.64E-05	4.87446
6674	SPAG1	0.62083	4.80339	8.140379	2.05E-06	1.64E-05	4.44915
7576	ZNF28	-0.6292	5.17566	-8.13962	2.06E-06	1.64E-05	4.36897
8797	TNFRSF10A	0.75984	4.18062	8.137495	2.06E-06	1.64E-05	4.58763
6636	SNRPF	0.59887	5.86006	8.130822	2.08E-06	1.66E-05	4.23612
7444	VRK2	-0.7368	4.03545	-8.12578	2.10E-06	1.67E-05	4.59912
79788	ZNF665	-1.54	2.03365	-8.12403	2.10E-06	1.67E-05	5.10552
144453	BEST3	1.87856	1.40597	8.123853	2.10E-06	1.67E-05	5.22615
58489	ABHD17C	-0.9186	3.35527	-8.11354	2.13E-06	1.69E-05	4.75652
64073	C19orf33	-0.6339	5.92612	-8.10813	2.15E-06	1.70E-05	4.19035
92715	DPH7	0.83897	4.49433	8.10745	2.15E-06	1.70E-05	4.4762
130497	OSR1	-0.8449	3.60065	-8.10646	2.15E-06	1.70E-05	4.68238
5495	PPM1B	-0.5849	4.93627	-8.10638	2.15E-06	1.70E-05	4.36892
10555	AGPAT2	0.60815	6.00898	8.082883	2.22E-06	1.75E-05	4.14418
5144	PDE4D	-1.1263	2.5736	-8.0812	2.22E-06	1.75E-05	4.91405
65999	LRRC61	1.65325	1.88107	8.071806	2.25E-06	1.77E-05	5.07299
4646	MYO6	-0.6776	4.76396	-8.07017	2.26E-06	1.77E-05	4.35488
8027	STAM	0.66213	4.92597	8.063309	2.28E-06	1.78E-05	4.31533
2717	GLA	0.68755	4.95209	8.062521	2.28E-06	1.78E-05	4.30973
489	ATP2A3	-2.4791	0.38276	-8.05832	2.29E-06	1.79E-05	5.23607
64115	VSIR	1.17885	2.47999	8.047448	2.33E-06	1.81E-05	4.89824
1801	DPH1	0.65362	4.27427	8.039641	2.35E-06	1.83E-05	4.42409
23240	TMEM131L	0.84944	3.43433	8.022318	2.41E-06	1.87E-05	4.61343
9260	PDLIM7	-0.5965	5.77752	-8.01859	2.42E-06	1.88E-05	4.08661
768	CA9	-4.215	-0.89158	-8.013	2.44E-06	1.89E-05	5.15929
149076	ZNF362	-0.7211	4.40237	-8.0111	2.44E-06	1.89E-05	4.35085
167359	NIM1K	-2.272	0.20994	-8.00843	2.45E-06	1.90E-05	5.17428
146227	BEAN1	-5.2452	0.21729	-8.47699	2.46E-06	1.90E-05	5.20812
29098	RANGRF	0.7621	4.01372	8.005802	2.46E-06	1.90E-05	4.4415
1820	ARID3A	-1.1628	2.46177	-8.00556	2.46E-06	1.90E-05	4.83535
56103	PCDHGB2	0.9754	3.9104	7.997477	2.49E-06	1.92E-05	4.46374
55788	LMBRD1	-0.8157	3.79793	-7.98652	2.53E-06	1.94E-05	4.46228
100131017	ZNF316	0.67834	5.0775	7.986483	2.53E-06	1.94E-05	4.17542
7280	TUBB2A	-0.6349	4.37411	-7.98252	2.54E-06	1.95E-05	4.31447
65267	WNK3	-2.6366	-0.31727	-7.97493	2.57E-06	1.96E-05	5.13038
3598	IL13RA2	1.94068	1.47938	7.971763	2.58E-06	1.97E-05	5.00928
390928	ACP7	0.70901	4.04421	7.967972	2.59E-06	1.98E-05	4.37792
10301	DLEU1	0.73789	4.20272	7.966511	2.60E-06	1.98E-05	4.33902

144455	E2F7	0.62657	6.26669	7.965556	2.60E-06	1.98E-05	3.93722
51763	INPP5K	0.6097	5.49469	7.958633	2.62E-06	2.00E-05	4.05386
55164	SHQ1	0.68396	4.58811	7.957053	2.63E-06	2.00E-05	4.23631
639	PRDM1	2.21922	0.24246	7.948663	2.66E-06	2.02E-05	5.09445
339290	LINC00667	0.94627	3.33749	7.948513	2.66E-06	2.02E-05	4.5365
5229	PGGT1B	-0.7372	5.0036	-7.94568	2.67E-06	2.02E-05	4.12882
101926978	LINC01111	2.13339	0.65154	7.944837	2.67E-06	2.02E-05	5.06747
26095	PTPN20	4.09854	-1.11471	7.944688	2.67E-06	2.02E-05	5.06922
201931	TMEM192	0.61183	4.64754	7.933845	2.71E-06	2.04E-05	4.18801
54944	LINC01521	-1.4215	1.97565	-7.92188	2.76E-06	2.07E-05	4.83018
79789	CLMN	-1.1516	3.30797	-7.91907	2.77E-06	2.08E-05	4.50229
6338	SCNN1B	-2.0507	0.98955	-7.89797	2.85E-06	2.13E-05	4.96754
6590	SLPI	-2.8248	-0.0411	-7.89562	2.86E-06	2.13E-05	5.02991
29785	CYP2S1	-0.7417	4.34317	-7.89166	2.87E-06	2.14E-05	4.19353
56667	MUC13	2.57463	0.54843	7.891347	2.87E-06	2.14E-05	5.00808
387804	VSTM5	-1.6124	1.75916	-7.89116	2.88E-06	2.14E-05	4.83479
63935	PCIF1	-0.5834	5.6819	-7.89114	2.88E-06	2.14E-05	3.91964
10351	ABCA8	1.30527	2.23114	7.890724	2.88E-06	2.14E-05	4.73754
54538	ROBO4	0.99198	4.43555	7.881179	2.92E-06	2.17E-05	4.17249
79098	C1orf116	1.76643	1.09573	7.879009	2.92E-06	2.17E-05	4.92741
9134	CCNE2	0.63365	4.96199	7.876273	2.93E-06	2.18E-05	4.03925
355	FAS	1.04416	2.72308	7.849958	3.04E-06	2.25E-05	4.55505
7789	ZXDA	1.20061	2.19156	7.849878	3.04E-06	2.25E-05	4.68379
79734	KCTD17	-0.7632	4.19895	-7.84378	3.07E-06	2.26E-05	4.15834
55100	WDR70	-0.6274	5.00668	-7.84179	3.08E-06	2.27E-05	3.97608
51427	ZNF107	0.67601	4.57018	7.841619	3.08E-06	2.27E-05	4.07354
115416	MALSU1	0.59245	4.74727	7.83657	3.10E-06	2.28E-05	4.02581
10782	ZNF274	1.13906	3.50825	7.833755	3.11E-06	2.29E-05	4.33793
1368	CPM	-0.7075	4.33815	-7.83188	3.12E-06	2.29E-05	4.10776
11010	GLIPR1	0.66584	3.96129	7.829601	3.13E-06	2.30E-05	4.19793
3459	IFNGR1	-0.646	4.40718	-7.8264	3.14E-06	2.30E-05	4.08254
100506190	LINC00963	0.59804	4.31953	7.820349	3.17E-06	2.32E-05	4.09727
114134	SLC2A13	-0.8849	3.31095	-7.81713	3.18E-06	2.32E-05	4.34518
1997	ELF1	0.69079	4.64892	7.816085	3.19E-06	2.33E-05	4.01978
53917	RAB24	-0.587	4.73577	-7.8102	3.21E-06	2.34E-05	3.98582
266722	HS6ST3	-2.6269	-0.50276	-7.80441	3.24E-06	2.36E-05	4.902
2925	GRPR	-0.8103	7.04579	-8.36907	3.26E-06	2.37E-05	3.73452
643641	ZNF862	0.88194	3.55091	7.796521	3.27E-06	2.38E-05	4.26167
284254	DYNAP	2.71602	-0.61882	7.795994	3.28E-06	2.38E-05	4.88324
57221	ARFGEF3	-1.0898	5.59103	-8.36062	3.29E-06	2.39E-05	3.94144
55020	TTC38	-0.5942	4.58752	-7.78697	3.32E-06	2.40E-05	3.98425
29080	CCDC59	0.58795	5.08349	7.785679	3.32E-06	2.40E-05	3.88207
1909	EDNRA	-2.0263	0.44273	-7.78488	3.33E-06	2.40E-05	4.85954
113622	ADPRHL1	0.87451	3.96459	7.784802	3.33E-06	2.40E-05	4.14038
10769	PLK2	0.72129	5.68375	7.784543	3.33E-06	2.40E-05	3.77083
8515	ITGA10	0.8723	3.6653	7.783435	3.33E-06	2.40E-05	4.21309
7079	TIMP4	2.47394	0.50542	7.782064	3.34E-06	2.41E-05	4.86001
26240	FAM50B	1.46124	1.67759	7.779669	3.35E-06	2.41E-05	4.69353
206338	LVRN	1.77549	0.86863	7.775764	3.37E-06	2.42E-05	4.80975
196792	FAM24B	0.99236	2.93656	7.767512	3.41E-06	2.45E-05	4.38069

11097	NUP42	0.63038	4.10419	7.760535	3.44E-06	2.47E-05	4.0619
2289	FKBP5	0.59391	4.82027	7.743776	3.52E-06	2.51E-05	3.87543
440482	ANKRD20A5P	-2.0345	1.0589	-7.74329	3.52E-06	2.51E-05	4.74508
54516	MTRF1L	-0.6198	4.50638	-7.74132	3.53E-06	2.52E-05	3.93621
9641	IKBKE	0.73808	3.61995	7.738423	3.55E-06	2.52E-05	4.15411
90007	MIDN	-0.7902	4.38579	-7.73711	3.55E-06	2.52E-05	3.96083
10788	IQGAP2	-2.1148	0.52177	-7.73302	3.57E-06	2.54E-05	4.7838
10217	CTDSPL	-2.7926	0.19503	-7.73081	3.58E-06	2.54E-05	4.80581
1373	CPS1	-0.7241	4.5943	-7.72725	3.60E-06	2.55E-05	3.89853
126731	CCSAP	-0.6317	4.74606	-7.72196	3.63E-06	2.56E-05	3.85603
9394	HS6ST1	0.66373	4.51429	7.706563	3.71E-06	2.61E-05	3.88912
3269	HRH1	-0.7148	3.65813	-7.70169	3.73E-06	2.63E-05	4.08333
4135	MAP6	-1.9567	0.81941	-7.70065	3.74E-06	2.63E-05	4.70996
10815	CPLX1	2.46319	0.55038	7.698286	3.75E-06	2.63E-05	4.74246
64838	FNDC4	-2.8578	0.2895	-7.69734	3.75E-06	2.63E-05	4.75769
100505633	LINC01133	-3.1093	-1.08118	-7.69477	3.77E-06	2.64E-05	4.73732
729830	FAM160A1	0.66496	4.75028	7.694074	3.77E-06	2.64E-05	3.81926
3352	HTR1D	-2.0191	0.51666	-7.69255	3.78E-06	2.64E-05	4.72686
3310	HSPA6	1.08476	4.43076	7.786441	3.80E-06	2.66E-05	3.92167
158835	AWAT2	1.75841	1.17703	7.679494	3.85E-06	2.69E-05	4.63772
27143	PALD1	-0.8706	3.50093	-7.67793	3.86E-06	2.69E-05	4.09356
2776	GNAQ	0.93206	3.34616	7.67714	3.86E-06	2.69E-05	4.14216
4148	MATN3	-1.6469	1.06916	-7.67374	3.88E-06	2.70E-05	4.63702
50937	CDON	-0.8423	4.65699	-7.66251	3.94E-06	2.74E-05	3.79293
91543	RSAD2	1.76878	1.42586	7.650795	4.00E-06	2.78E-05	4.56125
29948	OSGIN1	0.71624	5.02447	7.649783	4.01E-06	2.78E-05	3.69817
55006	TRMT61B	-0.5918	4.26169	-7.64752	4.02E-06	2.78E-05	3.85373
84306	PDCD2L	0.8253	3.46657	7.640114	4.06E-06	2.80E-05	4.05341
151176	ERFE	-1.216	2.6696	-7.62114	4.17E-06	2.87E-05	4.23706
374383	NCR3LG1	1.25046	2.61736	7.618113	4.19E-06	2.87E-05	4.25576
9880	ZBTB39	0.596	4.44274	7.615823	4.20E-06	2.88E-05	3.77012
9014	TAF1B	-0.6299	4.54731	-7.61508	4.21E-06	2.88E-05	3.74194
6621	SNAPC4	0.96864	3.44771	7.613731	4.22E-06	2.89E-05	4.02513
644100	ARL14EPL	1.13334	2.66044	7.60856	4.25E-06	2.91E-05	4.22569
6000	RGS7	2.12593	0.7876	7.606805	4.26E-06	2.91E-05	4.58926
7464	CORO2A	0.79287	3.47189	7.60623	4.26E-06	2.91E-05	4.00063
4312	MMP1	-0.6058	4.82369	-7.60108	4.29E-06	2.93E-05	3.66118
54541	DDIT4	-0.7996	4.1816	-7.60066	4.29E-06	2.93E-05	3.8087
10039	PARP3	0.62714	4.38705	7.599621	4.30E-06	2.94E-05	3.75964
728730	MAP4K3-DT	-1.1353	2.76735	-7.59742	4.31E-06	2.94E-05	4.17453
8825	LIN7A	-2.9884	-0.57194	-7.59117	4.35E-06	2.96E-05	4.61466
63920	ZBED8	-0.6545	3.90522	-7.58037	4.42E-06	3.00E-05	3.84149
91289	LMF2	-0.5924	6.32507	-7.5785	4.43E-06	3.01E-05	3.36087
283392	TRHDE-AS1	1.12774	2.75601	7.576129	4.44E-06	3.01E-05	4.15433
118987	PDZD8	-0.6102	6.62045	-7.56583	4.51E-06	3.05E-05	3.30177
377677	CA13	1.74965	0.90214	7.562111	4.53E-06	3.06E-05	4.50609
9619	ABCG1	1.08303	2.33656	7.561722	4.54E-06	3.06E-05	4.23235
79669	C3orf52	1.21192	1.90098	7.558881	4.55E-06	3.08E-05	4.32648
10536	P3H3	-0.7775	4.31397	-7.55779	4.56E-06	3.08E-05	3.71327
1515	CTSV	-0.9987	2.85273	-7.55777	4.56E-06	3.08E-05	4.08884

100287042	LOC100287042	-1.5037	1.6794	-7.55087	4.61E-06	3.11E-05	4.36029
1277	COL1A1	-0.7677	3.4851	-7.51104	4.87E-06	3.26E-05	3.84892
64420	SUSD1	0.66156	3.98867	7.510652	4.87E-06	3.26E-05	3.7235
29882	ANAPC2	0.66848	4.75724	7.504803	4.91E-06	3.29E-05	3.53814
27237	ARHGEF16	2.08926	0.19959	7.500389	4.94E-06	3.30E-05	4.48372
124739	USP43	2.00983	0.28792	7.477268	5.11E-06	3.40E-05	4.44542
222389	BEND7	0.75796	3.39143	7.475266	5.12E-06	3.41E-05	3.82656
2737	GLI3	-0.646	4.53414	-7.4702	5.16E-06	3.43E-05	3.53013
26038	CHD5	-2.3908	0.02014	-7.47019	5.16E-06	3.43E-05	4.45038
170689	ADAMTS15	-0.8383	3.26463	-7.46661	5.19E-06	3.44E-05	3.84221
60598	KCNK15	0.82781	3.94616	7.461696	5.22E-06	3.46E-05	3.66768
79832	QSER1	-0.6306	6.78472	-7.50119	5.29E-06	3.49E-05	3.12521
64777	RMND5B	-0.5922	5.06871	-7.45192	5.30E-06	3.49E-05	3.38874
2731	GLDC	-1.0858	3.73219	-7.45126	5.30E-06	3.50E-05	3.70802
219285	SAMD9L	0.88926	3.92814	7.445949	5.34E-06	3.52E-05	3.65087
51316	PLAC8	2.10723	0.12828	7.43902	5.39E-06	3.55E-05	4.40174
1936	EEF1D	2.685	1.483	7.515672	5.50E-06	3.61E-05	4.28322
154796	AMOT	-0.5911	4.63383	-7.42395	5.51E-06	3.61E-05	3.43792
7754	ZNF204P	-1.0079	3.34097	-7.42296	5.52E-06	3.61E-05	3.76356
53947	A4GALT	-2.0447	0.06216	-7.4166	5.57E-06	3.64E-05	4.37111
6275	S100A4	-0.6856	4.37857	-7.41374	5.59E-06	3.65E-05	3.48162
146206	CARMIL2	1.10957	2.62988	7.40781	5.64E-06	3.68E-05	3.93707
9580	SOX13	0.76566	4.65327	7.406737	5.65E-06	3.68E-05	3.41687
27010	TPK1	0.59232	4.27033	7.402301	5.68E-06	3.70E-05	3.49189
6446	SGK1	-0.7217	4.30202	-7.40002	5.70E-06	3.71E-05	3.47942
2643	GCH1	0.74705	3.68338	7.398969	5.71E-06	3.71E-05	3.63707
317649	EIF4E3	0.69369	4.17019	7.397916	5.72E-06	3.72E-05	3.51201
51531	TRMO	0.85607	3.18141	7.397326	5.72E-06	3.72E-05	3.76857
91966	EOLA1	0.77537	3.61403	7.395639	5.74E-06	3.72E-05	3.65103
1491	CTH	-0.9585	2.67151	-7.3928	5.76E-06	3.74E-05	3.89049
140710	SOGA1	-0.7994	7.04158	-7.89604	5.79E-06	3.75E-05	3.11966
147015	DHRS13	-1.1478	2.36219	-7.38282	5.84E-06	3.78E-05	3.9581
317761	C14orf39	0.81119	3.56431	7.380109	5.86E-06	3.79E-05	3.6419
5734	PTGER4	-0.5986	4.10761	-7.37383	5.92E-06	3.82E-05	3.48312
57181	SLC39A10	-0.6221	5.78768	-7.37295	5.92E-06	3.83E-05	3.13688
10379	IRF9	0.86605	4.45163	7.371762	5.93E-06	3.83E-05	3.41306
100288798	LOC100288798	1.38037	2.40152	7.37158	5.94E-06	3.83E-05	3.9502
27344	PCSK1N	-0.7428	4.53063	-7.36891	5.96E-06	3.84E-05	3.38099
29953	TRHDE	0.6216	4.95372	7.368048	5.97E-06	3.84E-05	3.29036
340526	RTL5	-1.5855	1.94657	-7.36721	5.97E-06	3.85E-05	4.04296
467	ATF3	1.0383	3.20031	7.365889	5.98E-06	3.85E-05	3.72453
7075	TIE1	-1.7077	0.86643	-7.36021	6.03E-06	3.88E-05	4.21645
7052	TGM2	0.58089	7.45328	7.411019	6.05E-06	3.89E-05	2.90843
283234	CCDC88B	1.21449	2.1427	7.357383	6.06E-06	3.89E-05	3.98045
2202	EFEMP1	1.14265	2.13326	7.356326	6.07E-06	3.89E-05	3.97822
3399	ID3	1.01259	2.58136	7.356121	6.07E-06	3.89E-05	3.86808
151827	LRRC34	0.95027	2.94741	7.35413	6.09E-06	3.90E-05	3.76835
55616	ASAP3	-0.7749	4.06726	-7.34405	6.17E-06	3.94E-05	3.4524
92935	MARS2	0.80153	3.5704	7.341067	6.20E-06	3.96E-05	3.58163
115123	MARCHF3	-1.3129	1.88035	-7.34002	6.21E-06	3.96E-05	4.00742

84888	SPPL2A	-0.682	4.35271	-7.33896	6.22E-06	3.96E-05	3.375
100506394	GAS6-DT	1.29743	2.04492	7.329929	6.30E-06	4.01E-05	3.96465
2852	GPER1	0.91835	4.00978	7.329387	6.30E-06	4.01E-05	3.45689
1896	EDA	-1.3816	1.99767	-7.32875	6.31E-06	4.01E-05	3.96787
172	AFG3L1P	0.88412	3.81674	7.32743	6.32E-06	4.01E-05	3.501
84973	SNHG7	1.32847	2.35849	7.327422	6.32E-06	4.01E-05	3.89211
152078	SLC66A1L	1.01288	2.6785	7.32447	6.35E-06	4.02E-05	3.79658
3280	HES1	-0.8525	4.39969	-7.31793	6.41E-06	4.05E-05	3.33677
57037	ANKMY2	0.58394	4.74211	7.315733	6.43E-06	4.06E-05	3.25519
11248	NXPH3	2.16513	-0.26831	7.29797	6.60E-06	4.14E-05	4.21809
84141	EVA1A	0.94264	3.68357	7.296562	6.61E-06	4.15E-05	3.49182
1396	CRIP1	1.08359	3.47735	7.293097	6.64E-06	4.16E-05	3.54626
57106	NAT14	-0.8502	3.63894	-7.29304	6.64E-06	4.16E-05	3.48525
8341	H2BC15	1.70531	1.02167	7.28964	6.67E-06	4.18E-05	4.098
25836	NIPBL	-0.8332	6.40635	-7.7738	6.75E-06	4.22E-05	3.03868
5071	PRKN	-2.2009	0.47343	-7.27824	6.79E-06	4.24E-05	4.14919
144404	TMEM120B	0.6029	5.27822	7.269596	6.87E-06	4.29E-05	3.07577
81788	NUAK2	1.90788	0.92105	7.265343	6.91E-06	4.32E-05	4.08124
2621	GAS6	0.60168	5.17856	7.257678	6.99E-06	4.36E-05	3.07718
7456	WIPF1	-1.0121	2.53928	-7.25489	7.02E-06	4.37E-05	3.7187
9886	RHOBTB1	-0.6617	4.15535	-7.25287	7.04E-06	4.38E-05	3.29027
10855	HPSE	0.88354	3.61204	7.247957	7.09E-06	4.40E-05	3.43381
57455	REXO1	0.6041	5.10729	7.246734	7.10E-06	4.41E-05	3.07479
10516	FBLN5	0.77203	3.31333	7.242028	7.15E-06	4.43E-05	3.4971
3229	HOXC13	-1.1764	1.9409	-7.23368	7.24E-06	4.48E-05	3.83187
6710	SPTB	1.1967	2.16143	7.233282	7.24E-06	4.48E-05	3.79056
285735	LINC00326	1.73558	1.37383	7.228342	7.29E-06	4.50E-05	3.95584
83461	CDCA3	-0.5903	5.05999	-7.21433	7.44E-06	4.58E-05	3.03083
80095	ZNF606	-0.8633	2.87639	-7.20924	7.50E-06	4.60E-05	3.55829
200916	RPL22L1	0.67545	3.75339	7.206941	7.52E-06	4.61E-05	3.32644
10964	IFI44L	1.25297	3.29502	7.205144	7.54E-06	4.62E-05	3.46894
148266	ZNF569	1.4931	1.66154	7.203063	7.56E-06	4.63E-05	3.85969
56901	NDUFA4L2	1.55747	1.27849	7.188958	7.72E-06	4.71E-05	3.90779
54532	USP53	0.66093	4.45259	7.18493	7.76E-06	4.73E-05	3.12208
2297	FOXD1	-0.6714	4.08941	-7.18238	7.79E-06	4.74E-05	3.19903
333	APLP1	-0.5848	4.52793	-7.16344	8.01E-06	4.85E-05	3.06602
6383	SDC2	1.02279	2.52209	7.158196	8.07E-06	4.89E-05	3.58585
345757	FAM174A	-0.5932	4.0814	-7.1517	8.15E-06	4.93E-05	3.15201
23259	DDHD2	-0.6048	5.54944	-7.14706	8.20E-06	4.96E-05	2.83405
100101467	ZSCAN30	-0.6446	3.99138	-7.14506	8.23E-06	4.97E-05	3.1651
79833	GEMIN6	-0.6313	4.02933	-7.14114	8.27E-06	4.99E-05	3.14952
4133	MAP2	-1.0276	2.33411	-7.1265	8.45E-06	5.07E-05	3.57639
389432	SAMD5	-0.6784	4.63471	-7.12637	8.45E-06	5.07E-05	2.98744
7179	TPTE	2.32793	0.36354	7.125497	8.46E-06	5.07E-05	3.94283
83593	RASSF5	0.65323	4.43927	7.105672	8.71E-06	5.21E-05	3.00334
9891	NUAK1	0.61427	4.57429	7.104065	8.73E-06	5.22E-05	2.96917
79632	FAM184A	-3.2985	-0.30466	-7.09925	8.80E-06	5.25E-05	3.94213
100507533	SOX21-AS1	-1.5825	1.15606	-7.0971	8.82E-06	5.27E-05	3.78582
24	ABCA4	-1.3989	1.50632	-7.09228	8.89E-06	5.30E-05	3.71366
10761	PLAC1	2.21729	0.77726	7.092002	8.89E-06	5.30E-05	3.85208

80774	LIMD2	-0.7589	3.89843	-7.08937	8.92E-06	5.31E-05	3.10536
55714	TENM3	2.17385	0.76514	7.085296	8.98E-06	5.34E-05	3.84143
138162	C9orf116	1.42943	1.92215	7.082376	9.02E-06	5.35E-05	3.62478
57545	CC2D2A	0.73105	4.36159	7.079972	9.05E-06	5.37E-05	2.98428
201134	CEP112	0.60068	4.06924	7.076517	9.09E-06	5.39E-05	3.04454
5592	PRKG1	-0.7139	3.97549	-7.07608	9.10E-06	5.39E-05	3.06482
57489	ODF2L	-0.8055	2.98879	-7.06651	9.23E-06	5.46E-05	3.30944
585	BBS4	0.63692	4.18235	7.065642	9.24E-06	5.46E-05	3.00138
7186	TRAF2	0.67172	4.56348	7.062813	9.28E-06	5.48E-05	2.90985
84503	ZNF527	1.48369	1.53696	7.062413	9.28E-06	5.48E-05	3.67117
3248	HPGD	0.71055	7.23554	7.517635	9.34E-06	5.51E-05	2.58799
84171	LOXL4	-0.789	3.98628	-7.05728	9.35E-06	5.52E-05	3.03495
55930	MYO5C	-0.7087	5.93945	-7.13704	9.38E-06	5.53E-05	2.65138
23541	SEC14L2	-0.7715	4.30205	-7.0526	9.42E-06	5.55E-05	2.95127
11123	RCAN3	-0.6302	4.53867	-7.04943	9.46E-06	5.56E-05	2.889
345557	PLCXD3	-0.7071	7.81374	-7.49546	9.61E-06	5.63E-05	2.49814
56935	SMCO4	-0.9461	2.53381	-7.03374	9.68E-06	5.66E-05	3.38218
26160	IFT172	-0.6977	4.11977	-7.03143	9.71E-06	5.67E-05	2.96016
10404	CPQ	0.67783	4.08884	7.024593	9.81E-06	5.72E-05	2.96167
958	CD40	-0.8249	3.0734	-7.01848	9.90E-06	5.77E-05	3.21371
84874	ZNF514	-0.6344	4.32918	-7.01468	9.96E-06	5.79E-05	2.88307
388	RHOB	-0.9392	3.55676	-6.99776	1.02E-05	5.90E-05	3.05907
5293	PIK3CD	-0.8288	3.20705	-6.99385	1.03E-05	5.93E-05	3.14038
8226	PUDP	0.5982	4.3398	6.992117	1.03E-05	5.94E-05	2.84906
7984	ARHGEF5	0.93695	2.87941	6.975011	1.06E-05	6.08E-05	3.20979
22944	KIN	0.96193	3.11594	6.974415	1.06E-05	6.09E-05	3.14638
10882	C1QL1	-0.8287	3.41517	-6.96746	1.07E-05	6.13E-05	3.04413
50944	SHANK1	0.87507	2.87595	6.963818	1.07E-05	6.16E-05	3.19065
25960	ADGRA2	-1.2816	2.19547	-6.96052	1.08E-05	6.19E-05	3.36491
154214	RNF217	-0.6975	3.87049	-6.95112	1.09E-05	6.25E-05	2.89693
5141	PDE4A	-1.2989	2.03482	-6.9458	1.10E-05	6.30E-05	3.37995
54587	MXRA8	-0.9045	2.46029	-6.94438	1.10E-05	6.31E-05	3.26201
7593	MZF1	0.72987	3.48805	6.934989	1.12E-05	6.40E-05	2.97833
627	BDNF	0.9687	2.43546	6.925444	1.14E-05	6.47E-05	3.24858
2395	FXN	0.77909	3.8439	6.924926	1.14E-05	6.47E-05	2.87255
9586	CREB5	-0.5885	4.68964	-6.92122	1.14E-05	6.50E-05	2.65523
64067	NPAS3	-1.5806	1.77266	-6.91903	1.15E-05	6.51E-05	3.40583
995	CDC25C	-0.7311	4.38632	-6.91867	1.15E-05	6.51E-05	2.72221
26086	GPSM1	0.69778	4.44211	6.918668	1.15E-05	6.51E-05	2.71381
130557	ZNF513	-0.6841	3.81178	-6.9121	1.16E-05	6.57E-05	2.85028
84166	NLRC5	1.4306	1.84641	6.908646	1.16E-05	6.58E-05	3.37581
60489	APOBEC3G	1.36716	2.33591	6.908467	1.17E-05	6.58E-05	3.26377
344887	NMRAL2P	1.70114	1.5298	6.908161	1.17E-05	6.58E-05	3.4464
81605	URM1	0.59203	5.89307	6.906529	1.17E-05	6.59E-05	2.40397
151242	PPP1R1C	1.93119	0.10589	6.891855	1.19E-05	6.72E-05	3.61394
8798	DYRK4	0.99268	2.87763	6.889613	1.20E-05	6.74E-05	3.07971
6755	SSTR5	-1.1378	3.00208	-6.88148	1.21E-05	6.81E-05	3.03232
53944	CSNK1G1	0.59528	5.9958	6.877891	1.22E-05	6.83E-05	2.34221
79094	CHAC1	0.58061	4.25865	6.877868	1.22E-05	6.83E-05	2.68911
64421	DCLRE1C	0.67045	4.03576	6.877184	1.22E-05	6.84E-05	2.74474

55290	BRF2	0.60279	4.87552	6.876158	1.22E-05	6.84E-05	2.54883
9170	LPAR2	0.76945	3.47984	6.871046	1.23E-05	6.89E-05	2.88262
254263	CNIH2	-1.2745	2.2843	-6.8603	1.25E-05	6.99E-05	3.18856
196500	PIANP	-2.4554	0.16769	-6.85702	1.26E-05	7.00E-05	3.5627
93663	ARHGAP18	0.64	4.34701	6.852708	1.27E-05	7.04E-05	2.63044
3556	IL1RAP	0.59773	3.95467	6.844543	1.28E-05	7.12E-05	2.71139
284370	ZNF615	1.11744	2.7462	6.844022	1.28E-05	7.12E-05	3.0492
80328	ULBP2	1.04389	2.03966	6.842268	1.29E-05	7.14E-05	3.21645
11133	KPTN	-0.7421	3.0686	-6.838	1.29E-05	7.17E-05	2.93172
170712	COX7B2	0.79144	3.45018	6.832316	1.31E-05	7.23E-05	2.83023
339803	LOC339803	-0.6549	3.88003	-6.8231	1.32E-05	7.32E-05	2.69288
9205	ZMYM5	0.69638	3.81257	6.819521	1.33E-05	7.35E-05	2.71186
79815	NIPAL2	-0.9307	2.74437	-6.78264	1.41E-05	7.71E-05	2.93764
57722	IGDCC4	2.67299	-0.92276	6.782095	1.41E-05	7.71E-05	3.48927
11227	GALNT5	-2.9247	-0.92915	-6.77923	1.41E-05	7.74E-05	3.48522
2886	GRB7	1.20637	1.82575	6.773495	1.43E-05	7.80E-05	3.16215
129080	EMID1	-1.4742	1.06342	-6.77194	1.43E-05	7.81E-05	3.30642
57171	DOLPP1	0.61695	5.10822	6.768401	1.44E-05	7.85E-05	2.33074
11037	STON1	-1.3625	2.11459	-6.76811	1.44E-05	7.85E-05	3.08828
94030	LRRC4B	-1.8816	0.81083	-6.76195	1.45E-05	7.92E-05	3.33838
999	CDH1	-1.6752	1.00922	-6.76086	1.45E-05	7.93E-05	3.3026
8458	TTF2	-0.6062	5.04742	-6.75342	1.47E-05	8.00E-05	2.31551
100287036	LOC100287036	-1.4171	1.74048	-6.75162	1.47E-05	8.02E-05	3.14788
54873	PALMD	-1.8675	1.06251	-6.75042	1.48E-05	8.03E-05	3.28415
7039	TGFA	0.92529	2.6739	6.734726	1.51E-05	8.19E-05	2.88812
4773	NFATC2	-0.5923	5.51249	-6.73408	1.51E-05	8.20E-05	2.19425
8694	DGAT1	-0.592	6.08712	-6.72927	1.52E-05	8.26E-05	2.08936
135138	PACRG	-2.3169	-0.27303	-6.72835	1.53E-05	8.26E-05	3.39728
80210	ARMC9	-0.5912	4.45225	-6.72509	1.53E-05	8.30E-05	2.39854
100499467	LINC00673	-0.619	4.33491	-6.72195	1.54E-05	8.33E-05	2.42144
84870	RSPO3	-2.4248	-0.26582	-6.72105	1.54E-05	8.33E-05	3.38728
10333	TLR6	1.19622	2.01998	6.709786	1.57E-05	8.46E-05	3.01982
101927870	LOC101927870	-0.8231	3.10276	-6.70832	1.57E-05	8.47E-05	2.72085
4222	MEOX1	-2.4377	-0.55072	-6.70763	1.57E-05	8.47E-05	3.37818
7490	WT1	-1.1156	2.05536	-6.70307	1.59E-05	8.52E-05	2.99187
56097	PCDHGC5	-0.959	2.78403	-6.69741	1.60E-05	8.58E-05	2.7935
54556	ING3	0.6676	3.61344	6.687534	1.62E-05	8.70E-05	2.55377
4982	TNFRSF11B	-2.2976	-0.6346	-6.6872	1.62E-05	8.70E-05	3.3495
55722	CEP72	-0.8906	3.37544	-6.68097	1.64E-05	8.76E-05	2.60668
129285	PPP1R21	-0.6446	4.5953	-6.67449	1.66E-05	8.83E-05	2.28662
25819	NOCT	1.0611	3.04077	6.671977	1.66E-05	8.86E-05	2.69716
57494	RIMKLB	0.654	3.64711	6.669731	1.67E-05	8.88E-05	2.51579
94121	SYTL4	0.77007	3.21274	6.661642	1.69E-05	8.97E-05	2.62193
59344	ALOXE3	1.33679	1.54435	6.66018	1.69E-05	8.99E-05	3.04801
79072	FASTKD3	-0.6745	3.48346	-6.64719	1.73E-05	9.16E-05	2.51739
89953	KLC4	-0.6134	3.95483	-6.64271	1.74E-05	9.20E-05	2.38656
140886	PABPC5	0.87191	2.74835	6.636607	1.75E-05	9.28E-05	2.71078
390916	NUDT19	0.6306	4.06036	6.634229	1.76E-05	9.31E-05	2.35259
1356	CP	-2.758	-0.81819	-6.63046	1.77E-05	9.35E-05	3.26963
56521	DNAJC12	0.9614	2.79037	6.627048	1.78E-05	9.39E-05	2.68874

2946	GSTM2	-0.7242	3.27152	-6.62039	1.80E-05	9.48E-05	2.53248
100505894	TMEM161B-AS1	-1.2531	1.50722	-6.61667	1.81E-05	9.52E-05	2.97968
55432	YOD1	0.61691	5.43118	6.60601	1.84E-05	9.66E-05	2.00783
339804	C2orf74	-0.7715	3.36075	-6.60493	1.84E-05	9.67E-05	2.48554
8462	KLF11	-0.5842	3.88504	-6.59654	1.86E-05	9.78E-05	2.32934
9736	USP34	-0.5824	7.36301	-6.79481	1.90E-05	9.93E-05	1.75813
126353	MISP	1.1334	2.48101	6.57763	1.92E-05	0.0001003	2.69873
348487	FAM131C	-1.2455	1.54252	-6.57379	1.93E-05	0.0001008	2.90446
978	CDA	0.75532	4.31879	6.568143	1.95E-05	0.0001016	2.18772
85395	FAM207A	0.64899	4.3692	6.562774	1.96E-05	0.0001022	2.16387
5055	SERPINB2	-1.9233	0.28157	-6.56147	1.97E-05	0.0001024	3.09672
124976	SPNS2	-1.6529	0.7269	-6.55427	1.99E-05	0.0001034	3.02506
57536	KIAA1328	-1.1294	2.55207	-6.54387	2.02E-05	0.0001045	2.61687
1870	E2F2	0.89468	2.51296	6.53898	2.04E-05	0.0001051	2.6184
154043	CNKSR3	-0.7661	3.49234	-6.53395	2.05E-05	0.0001058	2.33587
284802	FRG1BP	3.60268	-1.22754	6.519334	2.10E-05	0.000108	3.10635
4325	MMP16	-0.6587	5.34297	-6.51856	2.10E-05	0.0001081	1.88082
343099	CCDC18	-0.6428	3.66966	-6.51587	2.11E-05	0.0001085	2.25632
221395	ADGRF5	2.17323	0.02641	6.514712	2.11E-05	0.0001085	3.05739
55502	HES6	-2.6137	0.50867	-6.51218	2.12E-05	0.0001089	3.01091
11322	TMC6	1.02127	2.61857	6.511255	2.12E-05	0.000109	2.55138
401074	LINC00960	1.31768	1.80161	6.509245	2.13E-05	0.0001092	2.75588
57475	PLEKHH1	-0.9883	2.94249	-6.50278	2.15E-05	0.0001102	2.44216
29103	DNAJC15	0.88383	3.23068	6.502188	2.15E-05	0.0001102	2.36686
90525	SHF	-1.549	0.96547	-6.50105	2.16E-05	0.0001104	2.90397
151050	KANSL1L	-1.1177	2.30805	-6.50061	2.16E-05	0.0001104	2.61006
2212	FCGR2A	-0.6878	4.59186	-6.49771	2.17E-05	0.0001108	2.00386
10410	IFITM3	0.62396	7.58522	6.870044	2.20E-05	0.0001121	1.63501
57134	MAN1C1	-2.0877	-0.06213	-6.48612	2.21E-05	0.0001124	3.01618
6550	SLC9A3	-1.191	1.59088	-6.48419	2.22E-05	0.0001126	2.75106
4648	MYO7B	1.20336	2.06914	6.481076	2.23E-05	0.0001131	2.64717
89846	FGD3	1.39431	1.2925	6.475299	2.25E-05	0.0001138	2.80738
5990	RFX2	-0.7308	3.38214	-6.46856	2.27E-05	0.0001149	2.25885
65250	CPLANE1	-0.6029	4.96793	-6.46499	2.28E-05	0.0001154	1.86786
23531	MMD	-0.6732	3.54254	-6.46479	2.28E-05	0.0001154	2.20838
6196	RPS6KA2	-1.9003	0.45879	-6.46226	2.29E-05	0.0001158	2.92296
83850	ESYT3	-1.9013	-0.31407	-6.44235	2.36E-05	0.000119	2.96547
9668	ZNF432	0.71761	3.49874	6.439025	2.38E-05	0.0001194	2.18621
93953	GCNA	1.09284	1.78704	6.438591	2.38E-05	0.0001195	2.63879
51313	GASK1B	-2.0558	0.57238	-6.43472	2.39E-05	0.00012	2.86994
400745	SH2D5	0.76467	2.99335	6.415378	2.46E-05	0.000123	2.2852
1410	CRYAB	1.61838	1.55126	6.409984	2.49E-05	0.0001237	2.66062
282996	RBM20	-1.9864	0.60922	-6.40587	2.50E-05	0.0001244	2.81838
8821	INPP4B	1.12758	1.93477	6.404832	2.50E-05	0.0001245	2.55239
4319	MMP10	-2.103	-0.43008	-6.39951	2.53E-05	0.0001253	2.90927
147	ADRA1B	-1.5465	1.63026	-6.39942	2.53E-05	0.0001253	2.61778
23345	SYNE1	0.80993	4.24835	6.392134	2.55E-05	0.0001265	1.92071
9712	USP6NL	0.61985	4.34646	6.388045	2.57E-05	0.0001272	1.88431
133522	PPARGC1B	0.67826	3.36011	6.383334	2.59E-05	0.000128	2.13103
79729	SH3D21	-0.6961	3.82873	-6.3755	2.62E-05	0.0001294	1.9885

344901	OSTN	-1.3743	1.94652	-6.3751	2.62E-05	0.0001295	2.50453
79822	ARHGAP28	-0.6477	4.45284	-6.37247	2.63E-05	0.00013	1.83017
5175	PECAM1	1.30086	1.49569	6.369926	2.64E-05	0.0001303	2.5976
64129	TINAGL1	3.77076	-0.44609	6.434266	2.65E-05	0.0001303	2.89034
388335	TMEM220	1.02041	1.9791	6.356241	2.70E-05	0.0001326	2.46037
58480	RHOU	-0.9993	3.86405	-6.41978	2.71E-05	0.0001327	1.97796
254827	NAALADL2	-0.9125	2.73382	-6.35152	2.72E-05	0.0001333	2.25073
2828	GPR4	-0.6806	4.22078	-6.34981	2.73E-05	0.0001335	1.8483
7113	TMPRSS2	-1.2448	1.45243	-6.34914	2.73E-05	0.0001335	2.56556
3899	AFF3	-0.7836	2.987	-6.3457	2.75E-05	0.0001341	2.16763
56144	PCDHA4	1.65012	0.40092	6.343645	2.76E-05	0.0001345	2.74382
60490	PPCDC	0.70599	3.87689	6.334293	2.80E-05	0.0001359	1.91553
286343	LURAP1L	-0.6123	4.2966	-6.32708	2.83E-05	0.0001371	1.79154
9148	NEURL1	-1.0789	1.68929	-6.32605	2.83E-05	0.0001372	2.47347
79723	SUV39H2	0.68144	3.52877	6.32474	2.84E-05	0.0001375	1.99024
3174	HNF4G	-0.7561	3.29987	-6.3148	2.88E-05	0.0001394	2.03134
84068	SLC10A7	0.64615	3.78597	6.313099	2.89E-05	0.0001396	1.90218
92270	ATP6AP1L	-1.524	0.73924	-6.3114	2.90E-05	0.0001399	2.6393
50651	SLC45A1	-1.3018	1.29234	-6.30934	2.91E-05	0.0001403	2.53458
388228	SBK1	-1.6081	1.08639	-6.30611	2.92E-05	0.0001408	2.5763
8464	SUPT3H	-0.6586	3.48156	-6.30489	2.93E-05	0.000141	1.9632
843	CASP10	1.18795	2.4631	6.29781	2.96E-05	0.0001424	2.25363
1875	E2F5	-0.6644	3.78896	-6.29543	2.97E-05	0.0001427	1.86642
26108	PYGO1	-0.6824	3.48809	-6.29369	2.98E-05	0.0001429	1.94362
84964	ALKBH6	0.87785	2.82618	6.293112	2.98E-05	0.000143	2.13617
57690	TNRC6C	1.00097	2.66438	6.289413	3.00E-05	0.0001437	2.17894
100288181	LNCOC1	0.92677	3.03443	6.289263	3.00E-05	0.0001437	2.07503
152225	LINC02085	0.78171	2.98346	6.285519	3.02E-05	0.0001442	2.07668
4326	MMP17	0.81307	4.6282	6.340116	3.06E-05	0.000146	1.66426
120071	LARGE2	-1.8873	0.02377	-6.27624	3.06E-05	0.0001461	2.68015
84329	HVCN1	1.01016	2.51541	6.271796	3.08E-05	0.0001469	2.18983
5099	PCDH7	0.97766	3.32517	6.263605	3.12E-05	0.0001484	1.95627
64919	BCL11B	-1.837	-0.04274	-6.26061	3.14E-05	0.000149	2.6617
7402	UTRN	-0.6724	6.95232	-6.60037	3.19E-05	0.0001514	1.30589
8624	PSMG1	0.58858	5.43125	6.247399	3.20E-05	0.0001518	1.42158
2246	FGF1	0.98122	2.60114	6.243281	3.23E-05	0.0001527	2.11923
2983	GUCY1B1	-0.6012	3.8152	-6.23851	3.25E-05	0.0001536	1.76404
54522	ANKRD16	0.91636	2.28794	6.231822	3.28E-05	0.000155	2.17876
154091	SLC2A12	-0.753	3.55453	-6.21577	3.37E-05	0.0001581	1.79994
257068	PLCXD2	1.47601	2.43993	6.221078	3.42E-05	0.0001602	2.1291
330	BIRC3	1.06556	3.61182	6.265715	3.43E-05	0.0001606	1.8074
10062	NR1H3	0.66617	4.20474	6.203559	3.43E-05	0.0001607	1.61579
118491	CFAP70	1.35184	1.30616	6.19117	3.50E-05	0.0001633	2.34828
9912	ARHGAP44	1.68683	0.28595	6.182687	3.55E-05	0.0001651	2.50394
342979	PALM3	-0.7659	3.79501	-6.18014	3.56E-05	0.0001656	1.677
7043	TGFB3	0.61885	3.72978	6.179157	3.57E-05	0.0001658	1.69441
22854	NTNG1	-0.7477	3.20303	-6.17618	3.59E-05	0.0001665	1.82842
27433	TOR2A	0.8105	2.58282	6.169331	3.63E-05	0.000168	1.99579
124149	ANKRD26P1	1.98228	-0.11936	6.15811	3.69E-05	0.0001707	2.51302
54816	ZNF280D	-0.6844	4.12212	-6.15042	3.74E-05	0.0001725	1.54277

10046	MAMLD1	0.64522	3.51488	6.150268	3.74E-05	0.0001725	1.7043
375341	C3orf62	0.81855	2.84983	6.14943	3.74E-05	0.0001726	1.8899
9508	ADAMTS3	0.73816	3.38946	6.148758	3.75E-05	0.0001726	1.73912
10267	RAMP1	-0.6063	5.66982	-6.16409	3.75E-05	0.0001726	1.21569
8187	ZNF239	-0.8711	2.99721	-6.14522	3.77E-05	0.0001734	1.83694
220992	ZNF485	0.81822	2.50154	6.138809	3.81E-05	0.0001748	1.9669
6372	CXCL6	-0.8249	2.4662	-6.13745	3.81E-05	0.0001752	1.96726
56911	MAP3K7CL	2.87936	-0.43514	6.133099	3.84E-05	0.0001761	2.5106
92255	LMBRD2	-0.7278	5.38427	-6.31236	3.84E-05	0.0001761	1.29995
22994	CEP131	-0.5916	4.33367	-6.12819	3.87E-05	0.0001773	1.45236
395	ARHGAP6	-0.6852	3.44555	-6.11985	3.92E-05	0.0001791	1.66721
8660	IRS2	-1.2074	1.92947	-6.11714	3.94E-05	0.0001796	2.08226
83543	AIF1L	0.82761	3.08704	6.104038	4.02E-05	0.0001829	1.75039
8339	H2BC8	0.84799	2.95371	6.099977	4.05E-05	0.0001837	1.78027
55281	TMEM140	1.05414	2.57348	6.098863	4.06E-05	0.0001839	1.89093
55040	EPN3	1.84045	-0.07661	6.09713	4.07E-05	0.0001842	2.41036
57118	CAMK1D	0.88942	3.21792	6.096804	4.07E-05	0.0001843	1.70469
1951	CELSR3	0.59566	3.84288	6.091748	4.10E-05	0.0001857	1.51815
54622	ARL15	-1.0877	1.9842	-6.08842	4.12E-05	0.0001866	2.01701
4600	MX2	2.46637	-0.05081	6.087932	4.13E-05	0.0001866	2.40571
8436	CAVIN2	2.24476	-0.19076	6.077532	4.20E-05	0.0001893	2.39647
3981	LIG4	0.69405	3.64575	6.068909	4.25E-05	0.0001917	1.53531
4602	MYB	-0.8315	3.07409	-6.06595	4.27E-05	0.0001924	1.68315
6399	TRAPPC2	0.61638	3.81768	6.062999	4.30E-05	0.0001931	1.47723
9200	HACD1	1.12567	1.72872	6.057136	4.34E-05	0.0001948	2.03254
84315	MON1A	0.73683	3.98193	6.054204	4.36E-05	0.0001955	1.42478
4084	MXD1	0.79071	2.9819	6.045146	4.42E-05	0.0001977	1.67886
33	ACADL	-1.4467	0.95292	-6.04511	4.42E-05	0.0001977	2.17273
63899	NSUN3	0.69108	3.3746	6.042494	4.44E-05	0.0001983	1.56371
196513	DCP1B	0.93198	2.10922	6.040556	4.45E-05	0.0001987	1.90839
55824	PAG1	-0.6702	3.40127	-6.03298	4.51E-05	0.0002008	1.53379
144535	CFAP54	1.56975	0.38984	6.032438	4.51E-05	0.0002009	2.247
200576	PIKFYVE	-0.6404	6.21201	-6.26105	4.55E-05	0.0002027	0.99855
84734	FAM167B	2.06559	0.59209	6.026077	4.56E-05	0.0002028	2.22131
80709	AKNA	0.89202	2.778	6.023474	4.58E-05	0.0002035	1.70284
79690	GAL3ST4	1.1797	1.69485	6.017019	4.62E-05	0.0002052	1.97601
284759	SIRPB2	-1.4694	0.57863	-6.0143	4.64E-05	0.0002059	2.1841
79173	BRME1	0.58273	3.64149	6.007657	4.69E-05	0.0002077	1.42984
9476	NAPSA	1.662	-0.1647	5.991621	4.82E-05	0.0002124	2.24779
100131827	ZNF717	0.87263	2.97394	5.978544	4.92E-05	0.0002164	1.57228
101927789	LOC101927789	-0.6368	3.37607	-5.97776	4.93E-05	0.0002165	1.44637
441666	LOC441666	0.85983	2.92028	5.975003	4.95E-05	0.0002172	1.58103
84541	KBTBD8	2.74732	-0.01106	5.974453	4.95E-05	0.0002174	2.22643
728613	LOC728613	-0.8064	3.24549	-5.97344	4.96E-05	0.0002176	1.48014
153218	SPINK13	1.52878	0.56936	5.966464	5.02E-05	0.0002197	2.11371
57182	ANKRD50	0.63786	6.42645	6.226358	5.05E-05	0.0002209	0.87147
79962	DNAJC22	0.66848	3.76204	5.961042	5.06E-05	0.0002212	1.32208
8784	TNFRSF18	-1.904	0.0513	-5.95864	5.08E-05	0.0002219	2.17251
729737	LOC729737	1.06555	1.58145	5.954758	5.11E-05	0.0002232	1.89352
64092	SAMSN1	1.80431	0.17306	5.954311	5.12E-05	0.0002232	2.15309

30061	SLC40A1	-0.7652	2.87331	-5.95152	5.14E-05	0.0002242	1.5441
54567	DLL4	1.32012	0.94699	5.95007	5.15E-05	0.0002245	2.01927
6340	SCNN1G	-2.059	0.81827	-5.94674	5.18E-05	0.0002254	2.05346
51166	AADAT	0.60968	3.75185	5.942224	5.22E-05	0.000227	1.29053
54438	GFOD1	0.60031	3.37052	5.93782	5.25E-05	0.0002286	1.38552
84958	SYTL1	1.52544	0.75912	5.936709	5.26E-05	0.0002289	2.03461
26353	HSPB8	1.42504	1.43046	5.932179	5.30E-05	0.0002304	1.90001
9625	AATK	-1.5089	0.48118	-5.92837	5.33E-05	0.0002317	2.05981
100506465	LINC01234	-0.6178	3.55981	-5.92173	5.39E-05	0.000234	1.30152
51365	PLA1A	1.71671	0.61393	5.902212	5.57E-05	0.0002406	2.00624
51599	LSR	-1.2265	1.88929	-5.89713	5.61E-05	0.0002423	1.72622
100130776	AGAP2-AS1	0.65067	4.47778	5.895241	5.63E-05	0.000243	1.03211
117854	TRIM6	0.71416	3.39675	5.891492	5.66E-05	0.0002444	1.30348
8869	ST3GAL5	-1.6575	0.92507	-5.87581	5.81E-05	0.0002499	1.90446
221662	RBM24	-0.6674	3.452	-5.86564	5.91E-05	0.0002537	1.23705
85016	CFAP300	-1.7395	0.17423	-5.8629	5.93E-05	0.0002546	1.99931
728411	GUSBP1	-0.8123	2.74678	-5.84682	6.09E-05	0.0002606	1.40369
92714	ARRDC1	0.62504	4.48098	5.842895	6.13E-05	0.0002619	0.94117
8818	DPM2	0.58664	5.95284	5.890058	6.20E-05	0.0002645	0.6564
2354	FOSB	1.32071	1.23293	5.834443	6.22E-05	0.0002652	1.77406
147923	ZNF420	0.71552	2.92912	5.830704	6.25E-05	0.0002667	1.32881
11031	RAB31	-0.9883	1.8444	-5.83028	6.26E-05	0.0002667	1.61649
131909	FAM172BP	1.65676	0.24389	5.829659	6.26E-05	0.0002668	1.93842
57531	HACE1	-0.6699	3.96704	-5.82827	6.28E-05	0.0002673	1.03699
84517	ACTRT3	0.77637	2.56296	5.827718	6.28E-05	0.0002674	1.42717
9839	ZEB2	1.50354	1.22119	5.825302	6.31E-05	0.0002683	1.76657
440957	SMIM4	0.6157	3.19166	5.82433	6.32E-05	0.0002686	1.24175
631	BFSP1	0.96651	2.34649	5.821056	6.35E-05	0.0002699	1.48062
3106	HLA-B	-0.7883	2.67265	-5.8183	6.38E-05	0.0002709	1.37431
101927780	LINC01303	1.27964	0.99562	5.815948	6.41E-05	0.0002715	1.78711
92922	CCDC102A	-0.6024	4.30092	-5.81524	6.41E-05	0.0002718	0.93063
116362	RBP7	0.73585	2.98212	5.811378	6.45E-05	0.0002732	1.28205
140685	ZBTB46	-0.8001	2.38945	-5.79982	6.58E-05	0.0002776	1.42044
56944	OLFML3	3.18163	-1.15211	5.797631	6.60E-05	0.0002785	2.00052
163702	IFNLR1	-0.8694	2.61505	-5.79695	6.61E-05	0.0002787	1.3566
286257	PAXX	0.74698	4.38769	5.798068	6.70E-05	0.0002819	0.87675
101929563	LOC101929563	1.50921	0.04351	5.780669	6.79E-05	0.0002851	1.88015
84102	SLC41A2	0.64583	3.73384	5.777041	6.83E-05	0.0002864	1.01517
9066	SYT7	-0.7652	2.6254	-5.77537	6.85E-05	0.0002871	1.31367
101927040	LOC101927040	1.51414	0.68819	5.774639	6.85E-05	0.0002873	1.77797
84830	ADTRP	1.31935	1.31984	5.765141	6.96E-05	0.0002911	1.6395
388341	LRRC75A	-0.998	1.57543	-5.75264	7.11E-05	0.0002961	1.5491
100506599	PPP1R26-AS1	0.72618	2.65124	5.750918	7.13E-05	0.0002966	1.27013
404550	C16orf74	0.64457	3.40226	5.737429	7.29E-05	0.0003023	1.03677
255057	CBARP	-1.7725	0.51767	-5.73653	7.30E-05	0.0003027	1.74405
345275	HSD17B13	-1.4067	0.72586	-5.73135	7.36E-05	0.0003049	1.69225
284521	OR2L13	-1.9684	-0.85551	-5.70906	7.64E-05	0.0003146	1.85037
23616	SH3BP1	-0.6426	3.54405	-5.70746	7.66E-05	0.0003152	0.93998
121227	LRIG3	0.65255	3.05236	5.701582	7.73E-05	0.0003178	1.07115
134285	TMEM171	0.61001	4.01421	5.695424	7.81E-05	0.00032	0.80052

4576	TRNT	-1.1689	1.70062	-5.69141	7.86E-05	0.000322	1.42042
10161	LPAR6	-0.6067	3.82158	-5.68403	7.96E-05	0.0003256	0.82476
1950	EGF	0.91586	2.45845	5.678835	8.03E-05	0.000328	1.20619
55180	LINS1	0.70472	3.71	5.675259	8.07E-05	0.0003293	0.84878
100289019	SLC25A25-AS1	1.16569	1.11307	5.672532	8.11E-05	0.0003307	1.52043
375444	C5orf34	-0.6866	3.55033	-5.67166	8.12E-05	0.000331	0.87771
4105	MAGEA6	-1.4544	0.44775	-5.66203	8.25E-05	0.0003353	1.62225
150776	SMPD4BP	0.63129	3.35372	5.661185	8.27E-05	0.0003357	0.91786
8913	CACNA1G	-3.2812	-1.07683	-5.65916	8.46E-05	0.0003425	1.76255
25780	RASGRP3	-1.6771	0.23845	-5.64296	8.52E-05	0.0003444	1.62659
100289097	FRG1CP	-0.6887	3.61277	-5.63839	8.58E-05	0.0003468	0.80368
728	C5AR1	3.87901	-1.52671	5.831182	8.66E-05	0.0003497	1.78484
347733	TUBB2B	-2.2974	-0.29419	-5.62829	8.73E-05	0.0003521	1.67757
57622	LRFN1	-0.6616	3.02107	-5.62681	8.75E-05	0.0003528	0.94527
7128	TNFAIP3	0.78986	3.73835	5.624092	8.79E-05	0.000354	0.75606
153571	C5orf38	-0.8844	2.29329	-5.62271	8.81E-05	0.0003547	1.14526
441054	C4orf47	-1.294	1.55341	-5.61745	8.89E-05	0.0003572	1.3329
200765	TIGD1	-0.7192	2.67263	-5.60135	9.13E-05	0.0003659	1.00036
51555	PEX5L	-1.2903	1.30628	-5.58025	9.46E-05	0.0003766	1.32281
2264	FGFR4	-0.6279	3.32559	-5.57963	9.47E-05	0.0003768	0.77782
29842	TFCP2L1	-3.2765	-1.1472	-5.5869	9.53E-05	0.000379	1.64741
360132	FKBP9P1	-1.0743	1.81761	-5.57189	9.59E-05	0.0003808	1.18451
5896	RAG1	1.27937	0.86317	5.571133	9.60E-05	0.0003811	1.39923
9881	TRANK1	0.83687	2.89582	5.56141	9.76E-05	0.0003863	0.87965
339324	ZNF260	0.59478	4.66772	5.559521	9.79E-05	0.0003871	0.40732
5581	PRKCE	-0.6486	3.38625	-5.55221	9.91E-05	0.0003915	0.71416
285550	FAM200B	0.63124	3.9023	5.539265	0.0001013	0.0003989	0.55838
1949	EFNB3	-0.6164	3.40449	-5.52979	0.0001029	0.0004042	0.66904
64940	STAG3L4	0.60605	3.49959	5.528382	0.0001031	0.0004049	0.64615
8345	H2BC9	-1.5997	-0.0063	-5.528	0.0001032	0.0004051	1.46519
55321	TMEM74B	-1.8347	-0.14934	-5.52548	0.0001036	0.0004064	1.48358
203054	ADCK5	-0.6115	3.64044	-5.52415	0.0001039	0.0004072	0.59437
91860	CALML4	0.61304	3.00558	5.518902	0.0001048	0.0004103	0.7664
26232	FBXO2	0.77797	2.7268	5.518725	0.0001048	0.0004103	0.85052
8497	PPFIA4	-0.6001	3.33437	-5.50665	0.000107	0.0004179	0.64771
8864	PER2	-0.6353	3.7393	-5.49764	0.0001086	0.0004238	0.52278
100130744	LOC100130744	-1.191	1.6885	-5.48778	0.0001104	0.0004291	1.07425
401039	LINC01940	-1.627	0.10182	-5.4865	0.0001106	0.0004299	1.38152
286333	FAM225A	1.6095	0.49518	5.482903	0.0001113	0.0004321	1.32062
57664	PLEKHA4	-1.9144	-0.61374	-5.47534	0.0001127	0.0004366	1.44912
100293516	ZNF587B	0.74013	2.94195	5.474546	0.0001129	0.0004369	0.71102
6335	SCN9A	0.76883	3.50445	5.471005	0.0001136	0.0004391	0.5502
6252	RTN1	-2.165	-0.51211	-5.47057	0.0001136	0.0004393	1.43545
1748	DLX4	-1.8928	-0.06266	-5.46894	0.000114	0.0004403	1.37907
4804	NGFR	1.92079	-0.4876	5.468348	0.0001141	0.0004406	1.42897
199704	ZNF585A	-2.5338	-0.71702	-5.45513	0.0001166	0.0004491	1.43351
57126	CD177	1.32429	0.38168	5.454267	0.0001168	0.0004496	1.28327
144577	C12orf66	0.7596	3.06839	5.447282	0.0001182	0.0004544	0.62818
128854	TSPY26P	-1.166	1.08013	-5.44666	0.0001183	0.0004547	1.13695
728215	FAM155A	-0.7421	3.33297	-5.44583	0.0001185	0.0004552	0.54531

100287082	GACAT2	0.86103	2.86599	5.444503	0.0001187	0.0004561	0.68488
378805	LINC-PINT	0.77295	2.5227	5.443077	0.000119	0.000457	0.77444
53820	RIPPLY3	-0.9665	1.97862	-5.43381	0.0001209	0.0004633	0.90234
138241	C9orf85	0.68128	3.34549	5.430129	0.0001217	0.0004659	0.51905
9823	ARMCX2	0.98874	1.78635	5.429206	0.0001219	0.0004665	0.94886
3604	TNFRSF9	1.82085	0.3499	5.426369	0.0001224	0.0004685	1.25344
79646	PANK3	-0.8298	5.22201	-5.67885	0.0001228	0.0004698	0.15289
342926	ZNF677	-1.4395	1.47396	-5.4215	0.0001234	0.0004719	1.01774
3757	KCNH2	-3.4038	-0.91766	-5.48372	0.0001239	0.0004734	1.40222
147372	CCBE1	0.89332	2.99575	5.419316	0.0001239	0.0004734	0.60585
26499	PLEK2	0.88033	2.2612	5.415992	0.0001246	0.0004758	0.8019
285753	CEP57L1	-0.7125	3.64744	-5.41515	0.0001248	0.0004763	0.40471
375704	ENHO	-2.0343	0.32891	-5.41229	0.0001254	0.0004777	1.23202
23109	DDN	1.56139	0.36135	5.408251	0.0001262	0.0004806	1.21375
152992	TRMT44	0.63749	3.19103	5.404265	0.0001271	0.0004834	0.51438
7507	XPA	0.68254	2.76689	5.403912	0.0001272	0.0004835	0.63424
115749	C12orf56	0.69844	2.61438	5.403122	0.0001273	0.0004839	0.67644
85319	BAGE2	-1.7491	0.60756	-5.40259	0.0001275	0.0004842	1.16454
132014	IL17RE	-1.3001	0.95472	-5.39888	0.0001283	0.0004868	1.08276
56477	CCL28	-0.7029	2.57128	-5.39585	0.0001289	0.0004887	0.66961
79817	MOB3B	-1.0452	1.94492	-5.39474	0.0001292	0.0004894	0.84581
158399	ZNF483	0.84723	2.55118	5.388218	0.0001306	0.000494	0.67313
57669	EPB41L5	-0.6814	4.03685	-5.3867	0.0001309	0.0004946	0.25145
8708	B3GALT1	-0.7155	3.23682	-5.38464	0.0001314	0.0004959	0.46353
90874	ZNF697	-0.876	2.6538	-5.38347	0.0001317	0.0004963	0.63062
79369	B3GNT4	-1.7505	-0.37485	-5.38153	0.0001321	0.0004973	1.26642
28968	SLC6A16	1.04533	1.45999	5.380781	0.0001323	0.0004977	0.943
5336	PLCG2	-0.7959	2.2659	-5.38049	0.0001323	0.0004978	0.72899
7832	BTG2	-1.2472	0.58505	-5.37587	0.0001334	0.0005009	1.10999
658	BMPR1B	-0.7199	3.72365	-5.35889	0.0001373	0.0005139	0.28486
349075	ZNF713	-1.0122	1.83044	-5.34672	0.0001401	0.0005236	0.78926
256126	SYCE2	-0.7667	2.20217	-5.34363	0.0001409	0.0005256	0.6807
91851	CHRDL1	-1.3898	0.82459	-5.34084	0.0001415	0.0005278	1.00915
5563	PRKAA2	-0.6459	4.7773	-5.36387	0.0001423	0.0005302	-0.00028
89874	SLC25A21	-1.0471	1.40119	-5.33359	0.0001433	0.0005337	0.86892
101060091	ACTR3-AS1	1.52839	-0.08523	5.323086	0.0001459	0.0005422	1.13154
727677	CASC8	1.02931	1.82139	5.321166	0.0001464	0.0005437	0.75291
5028	P2RY1	-0.6732	3.48982	-5.31969	0.0001467	0.0005449	0.27741
283314	C1RL-AS1	1.80092	1.09633	5.316086	0.0001476	0.0005473	0.93272
23312	DMXL2	-0.5835	5.77099	-5.44511	0.0001478	0.0005477	-0.1944
400720	ZNF772	0.65647	3.28142	5.311742	0.0001487	0.0005504	0.32592
4241	MELTF	0.64125	3.15134	5.309269	0.0001494	0.0005522	0.35771
157753	TMEM74	-1.4232	0.67188	-5.30547	0.0001503	0.0005552	0.97701
284161	GDPD1	0.74731	2.82156	5.300093	0.0001517	0.0005594	0.43767
84448	ABLIM2	-1.0044	1.97392	-5.29681	0.0001526	0.000562	0.66447
1838	DTNB	-0.6559	3.55202	-5.28846	0.0001548	0.0005692	0.20376
223117	SEMA3D	0.92268	4.65098	5.520325	0.0001567	0.0005747	0.0259
51085	MLXIPL	-2.2704	-0.79097	-5.27029	0.0001597	0.000584	1.12872
735301	SNHG9	0.95753	1.72803	5.268151	0.0001602	0.0005858	0.67991
101927455	TMEM108-AS1	2.01364	0.41299	5.264702	0.0001612	0.0005887	0.97248

222166	MTURN	-0.6129	3.04874	-5.25802	0.000163	0.000594	0.28895
56937	PMEPA1	-0.8644	2.54243	-5.25377	0.0001642	0.0005978	0.43111
51621	KLF13	-0.8823	1.70071	-5.25331	0.0001644	0.0005979	0.65347
221336	BEND6	-1.4103	0.56808	-5.24491	0.0001668	0.0006052	0.88995
100652748	TIMM23B	0.70654	2.95515	5.235022	0.0001696	0.0006137	0.28273
1911	PHC1	-0.9865	2.46529	-5.23044	0.0001709	0.0006174	0.41671
153443	SRFBP1	0.65149	3.71307	5.230206	0.000171	0.0006175	0.06242
8715	NOL4	1.54047	-0.25364	5.224396	0.0001727	0.0006226	0.98499
643827	CNTNAP3P2	-1.0325	1.88634	-5.21499	0.0001755	0.0006319	0.54335
5266	PI3	0.62194	4.08656	5.212373	0.0001763	0.000634	-0.06796
148213	ZNF681	0.77113	3.01712	5.21192	0.0001765	0.0006343	0.22639
100505783	OSER1-DT	-0.7086	2.54897	-5.20306	0.0001792	0.0006426	0.3346
9699	RIMS2	-1.8953	0.40974	-5.20012	0.0001801	0.0006453	0.85065
147906	DACT3	-1.5674	0.44733	-5.19824	0.0001807	0.0006472	0.83343
54932	EXD3	0.78141	2.33669	5.190403	0.0001831	0.0006549	0.37954
5097	PCDH1	1.15826	1.28759	5.188151	0.0001838	0.000657	0.64705
7145	TNS1	-0.5886	3.31026	-5.18591	0.0001846	0.0006589	0.08588
28986	MAGEH1	-1.1544	1.40342	-5.18426	0.0001851	0.0006606	0.60834
91694	LONRF1	1.15543	1.76551	5.172719	0.0001888	0.000672	0.50847
57396	CLK4	-0.7176	2.98128	-5.17013	0.0001896	0.0006744	0.15342
347746	PWARSN	-1.513	0.84702	-5.16957	0.0001898	0.0006749	0.71013
23150	FRMD4B	1.05652	3.07957	5.209383	0.0001901	0.0006756	0.16108
7096	TLR1	1.37741	0.50798	5.163408	0.0001919	0.0006813	0.76252
284348	LYPD5	-1.4244	0.51231	-5.16326	0.0001919	0.0006813	0.75788
2702	GJA5	1.27365	1.40561	5.163038	0.000192	0.0006814	0.57994
4883	NPR3	-1.6328	0.80091	-5.1588	0.0001934	0.0006851	0.70405
286437	LOC286437	0.88066	2.6483	5.158448	0.0001935	0.0006853	0.24073
79896	THNSL1	0.6209	3.30433	5.156484	0.0001942	0.000687	0.04139
79659	DYNC2H1	-0.5946	5.68349	-5.31561	0.0001958	0.0006918	-0.45634
222256	CDHR3	-0.7538	2.74446	-5.1476	0.0001972	0.0006959	0.18185
10580	SORBS1	-1.0759	1.43928	-5.13035	0.0002031	0.0007148	0.5037
23643	LY96	1.12704	1.24862	5.129616	0.0002034	0.0007155	0.55172
27094	KCNMB3	0.90535	1.9304	5.125064	0.000205	0.0007206	0.37456
2774	GNAL	1.20533	0.4649	5.122827	0.0002058	0.0007228	0.6949
283575	LINC02288	0.92014	1.80004	5.117204	0.0002078	0.0007291	0.39389
898	CCNE1	0.78312	1.96092	5.115342	0.0002085	0.0007312	0.34527
64651	CSRNP1	0.80149	2.93842	5.112489	0.0002095	0.0007346	0.07241
5154	PDGFA	0.67812	2.75513	5.112119	0.0002097	0.0007347	0.11922
84532	ACSS1	-1.0165	1.07786	-5.10866	0.0002109	0.0007385	0.54338
79816	TLE6	-1.6062	0.04216	-5.10711	0.0002115	0.0007399	0.74047
100506433	LINC00648	-0.6856	2.80908	-5.10666	0.0002116	0.0007402	0.0881
9480	ONECUT2	1.04867	2.57003	5.105224	0.0002122	0.0007418	0.17499
2898	GRIK2	-0.8795	1.9411	-5.10513	0.0002122	0.0007418	0.32982
4825	NKX6-1	-1.2903	0.67332	-5.0997	0.0002142	0.000748	0.61453
1852	DUSP9	-1.3742	0.71111	-5.09942	0.0002143	0.0007482	0.60807
2920	CXCL2	1.48621	0.61693	5.092537	0.0002169	0.0007549	0.62255
252995	FNDC5	-1.481	-0.34908	-5.08905	0.0002182	0.0007586	0.76
29993	PACSIN1	-0.8848	3.06445	-5.08528	0.0002197	0.0007624	-0.01731
54328	GPR173	0.64864	3.16773	5.081411	0.0002211	0.0007671	-0.05455
201181	ZNF385C	-1.2723	1.23113	-5.0779	0.0002225	0.0007713	0.46221

55176	SEC61A2	0.60957	3.10004	5.075187	0.0002235	0.0007744	-0.04807
6751	SSTR1	1.72986	-0.52317	5.074261	0.0002239	0.0007752	0.76312
2019	EN1	-0.8959	1.95204	-5.07277	0.0002245	0.0007768	0.26927
285352	KIF9-AS1	0.77121	2.24993	5.071661	0.0002249	0.0007781	0.19016
3676	ITGA4	1.72202	-0.17637	5.067685	0.0002265	0.0007826	0.70832
200035	NUDT17	1.2908	1.05905	5.05908	0.0002299	0.0007923	0.47148
94120	SYTL3	0.95703	1.80945	5.054556	0.0002317	0.0007974	0.28067
693199	MIR614	0.99899	1.11374	5.042614	0.0002366	0.0008117	0.42275
10149	ADGRG2	-3.1406	-0.85159	-5.08097	0.0002366	0.0008117	0.7662
51054	PLEKHA8P1	1.0225	1.12377	5.040746	0.0002373	0.0008136	0.41779
222962	SLC29A4	-0.8148	2.71098	-5.03378	0.0002402	0.0008219	-0.01193
388588	SMIM1	-1.6755	-0.19929	-5.02883	0.0002423	0.0008288	0.63996
58538	MPP4	1.25682	0.79241	5.026829	0.0002432	0.0008308	0.46677
5026	P2RX5	1.17439	0.56081	5.023394	0.0002446	0.0008343	0.50234
105373957	LOC105373957	-0.9447	1.69749	-5.01636	0.0002477	0.0008434	0.23464
1999	ELF3	-0.7213	2.53243	-5.01161	0.0002497	0.0008496	-0.00404
633	BGN	-1.3111	0.58603	-5.00971	0.0002506	0.0008519	0.47226
1288	COL4A6	0.75694	3.07636	5.009705	0.0002506	0.0008519	-0.15472
4832	NME3	-0.7293	3.203	-5.00845	0.0002511	0.0008536	-0.19965
342892	ZNF850	1.15896	1.31745	5.002625	0.0002537	0.0008605	0.31138
7294	TXK	1.39531	0.42288	5.000507	0.0002546	0.0008627	0.49135
268	AMH	1.85638	0.62828	4.998643	0.0002554	0.0008649	0.46584
100505761	RPARP-AS1	0.66011	2.73718	4.99654	0.0002564	0.0008671	-0.08453
51277	DNAJC27	-0.7302	2.36281	-4.99508	0.000257	0.0008687	0.01438
124923	RSKR	1.03327	1.095	4.977433	0.0002651	0.0008926	0.31134
653820	FAM72B	-0.6529	2.57369	-4.97641	0.0002656	0.0008939	-0.08105
6954	TCP11	-1.6231	-0.25061	-4.97369	0.0002669	0.0008977	0.54915
1241	LTB4R	0.68648	2.43299	4.972888	0.0002672	0.0008988	-0.04081
8029	CUBN	1.19477	0.60797	4.961491	0.0002726	0.0009137	0.38421
5909	RAP1GAP	-0.7914	2.96087	-4.95941	0.0002736	0.0009168	-0.21828
6262	RYR2	1.74797	0.94951	4.958672	0.000274	0.0009174	0.32978
375190	FAM228B	-0.9489	2.13987	-4.95025	0.000278	0.0009285	0.0014
120227	CYP2R1	0.88539	1.70804	4.950248	0.000278	0.0009285	0.11671
4067	LYN	-0.6364	2.95752	-4.93861	0.0002838	0.0009447	-0.25925
56906	THAP10	0.65624	3.06955	4.936159	0.000285	0.0009483	-0.28932
1543	CYP1A1	2.06743	-0.15757	4.928583	0.0002888	0.0009595	0.47258
9	NAT1	0.73057	2.19422	4.926873	0.0002897	0.0009616	-0.05695
105377621	LOC105377621	1.92158	-0.57919	4.918948	0.0002938	0.0009731	0.50458
492311	IGIP	-0.7852	2.0388	-4.90837	0.0002993	0.0009901	-0.05276
80778	ZNF34	-0.6043	3.33595	-4.90642	0.0003003	0.0009924	-0.42631
2494	NR5A2	-1.5172	0.08108	-4.90374	0.0003017	0.0009963	0.37616
414328	IDNK	0.6464	2.6729	4.903356	0.0003019	0.0009965	-0.23637
79887	PLBD1	-2.9985	-0.82221	-4.93265	0.0003055	0.0010067	0.51642
10154	PLXNC1	-0.7148	2.26253	-4.88446	0.0003122	0.0010268	-0.15888
10557	RPP38	0.78317	3.21373	4.879865	0.0003147	0.0010333	-0.42732
23639	LRRC6	-1.2179	0.70489	-4.87725	0.0003162	0.0010375	0.21163
255324	EPGN	-0.631	3.006	-4.8758	0.000317	0.0010399	-0.3878
2323	FLT3LG	0.90632	1.55254	4.873182	0.0003184	0.0010439	0.01619
55022	PID1	-0.8374	2.37604	-4.85588	0.0003283	0.0010702	-0.239
339366	ADAMTSL5	1.03885	1.86976	4.852856	0.0003301	0.0010754	-0.09516

79582	SPAG16	-0.6482	3.57495	-4.85	0.0003318	0.00108	-0.59456
29942	PURG	-1.1741	0.65385	-4.84648	0.0003338	0.0010859	0.16587
6094	ROM1	0.91692	2.41896	4.844837	0.0003348	0.0010883	-0.25998
728743	LOC728743	0.89395	2.57237	4.82917	0.0003442	0.0011154	-0.3338
283487	PRECSIT	0.86468	1.81432	4.82897	0.0003443	0.0011155	-0.13074
2825	GPR1	0.95228	1.79468	4.81404	0.0003536	0.001142	-0.14946
353497	POLN	-1.4145	-0.07714	-4.81029	0.0003559	0.0011487	0.23309
55659	ZNF416	0.58984	3.42453	4.80764	0.0003576	0.0011529	-0.62622
167838	TXLNB	1.76112	0.49785	4.780767	0.0003751	0.0012038	0.09845
54811	ZNF562	0.60252	3.59271	4.777011	0.0003776	0.0012094	-0.72905
4487	MSX1	0.76906	2.33913	4.776461	0.000378	0.0012103	-0.36889
199800	ADM5	1.88096	-0.67081	4.774767	0.0003791	0.0012136	0.26266
87769	GGACT	1.37492	0.18719	4.773099	0.0003802	0.0012163	0.1278
145553	MDP1	-1.1146	0.81786	-4.76616	0.0003849	0.0012289	-0.01276
170961	ANKRD24	0.92771	1.57055	4.754606	0.0003929	0.0012507	-0.20295
441150	C6orf226	-1.0673	1.03818	-4.7419	0.0004019	0.0012768	-0.10432
8382	NME5	1.11872	1.10074	4.739947	0.0004033	0.0012803	-0.11457
55068	ENOX1	-1.2095	0.489	-4.73439	0.0004074	0.0012917	-0.00315
144715	RAD9B	1.02607	1.37455	4.734261	0.0004074	0.0012917	-0.18939
9638	FEZ1	-1.7663	-0.23321	-4.72866	0.0004115	0.0013021	0.11877
102723356	ZFPM2-AS1	2.36006	-0.66516	4.725406	0.0004139	0.0013087	0.18639
23037	PDZD2	-0.6506	2.96424	-4.72376	0.0004152	0.0013119	-0.65433
101927027	CHROMR	0.58126	3.2444	4.722834	0.0004158	0.0013137	-0.73278
472	ATM	-0.7282	6.28581	-5.11194	0.0004199	0.0013237	-1.23805
1180	CLCN1	-0.6015	2.80424	-4.7108	0.0004249	0.0013353	-0.63353
2633	GBP1	-1.259	1.03529	-4.70958	0.0004258	0.0013372	-0.15748
596	BCL2	0.75699	2.7403	4.708179	0.0004269	0.0013399	-0.60859
84915	FAM222A	0.60513	3.30595	4.704126	0.00043	0.0013489	-0.78322
653857	ACTR3C	2.09609	-0.65644	4.700486	0.0004328	0.0013564	0.13523
645513	SEPTIN7P14	0.87388	1.89865	4.699836	0.0004333	0.0013573	-0.38782
56122	PCDHB14	-1.1666	0.48415	-4.68263	0.0004468	0.0013952	-0.09655
2743	GLRB	0.67619	2.7951	4.677314	0.0004511	0.0014058	-0.68444
23284	ADGRL3	-0.9687	1.48553	-4.67457	0.0004533	0.0014104	-0.33183
51501	HIKESHI	0.60299	3.67606	4.672649	0.0004549	0.0014145	-0.94433
6855	SYP	-1.1289	1.03569	-4.66996	0.0004571	0.0014203	-0.23247
100507002	LOC100507002	0.69865	2.46528	4.669563	0.0004574	0.001421	-0.60323
285958	SNHG15	0.60448	3.91569	4.667415	0.0004592	0.0014257	-1.01839
8329	H2AC13	1.26894	0.23749	4.659025	0.0004661	0.0014449	-0.08829
59285	CACNG6	1.32775	-0.05637	4.653015	0.0004712	0.0014577	-0.04874
25837	RAB26	-1.5901	0.64724	-4.62981	0.0004912	0.001514	-0.21335
634	CEACAM1	1.01329	1.06954	4.62372	0.0004966	0.0015295	-0.32239
389641	LOC389641	0.61618	3.11485	4.619233	0.0005006	0.0015406	-0.88604
100506504	PRKCZ-AS1	-1.1417	0.45107	-4.61835	0.0005014	0.0015423	-0.20744
101929340	LOC101929340	-1.4518	-0.24653	-4.61587	0.0005037	0.001548	-0.08771
441389	LINC01239	1.47789	0.09043	4.615506	0.000504	0.0015487	-0.13696
23007	PLCH1	0.59816	3.52101	4.612276	0.0005069	0.001556	-1.01416
728489	DNLZ	0.73043	2.49986	4.607009	0.0005118	0.0015678	-0.7273
339483	MTMR9LP	0.86198	1.7948	4.600071	0.0005182	0.001585	-0.5449
100288637	LOC100288637	-1.2784	0.06045	-4.59238	0.0005254	0.0016013	-0.18161
100128782	LINC00476	0.81049	1.96225	4.587764	0.0005298	0.0016135	-0.61236

7350	UCP1	1.39766	-0.55857	4.585009	0.0005324	0.0016208	-0.09576
23236	PLCB1	-0.5944	2.98054	-4.58399	0.0005334	0.0016234	-0.91908
285671	RNF180	-0.6861	2.33504	-4.57964	0.0005376	0.0016338	-0.73912
55762	ZNF701	-0.5967	3.5675	-4.57271	0.0005444	0.0016526	-1.1065
23526	ARHGAP45	-0.7093	2.45918	-4.57127	0.0005458	0.0016547	-0.79013
220441	RNF152	1.0344	1.11333	4.563129	0.0005539	0.0016772	-0.44229
221908	PPP1R35	-0.5891	3.53962	-4.56282	0.0005542	0.0016776	-1.11761
91683	SYT12	-1.268	0.19343	-4.56136	0.0005556	0.0016805	-0.26077
27128	CYTH4	-1.3202	-0.11174	-4.56063	0.0005564	0.0016819	-0.20964
145853	C15orf61	0.5909	2.6794	4.557754	0.0005593	0.0016891	-0.87558
57636	ARHGAP23	1.37711	0.07347	4.555958	0.0005611	0.0016934	-0.24392
389538	CCZ1P-OR7E38F	-0.7108	2.81327	-4.55143	0.0005657	0.0017063	-0.92891
5920	PLAAT4	0.96161	1.52474	4.550181	0.000567	0.0017087	-0.56461
57530	CGN	-0.6751	2.3877	-4.54215	0.0005753	0.0017304	-0.82434
151477	LINC00471	-1.266	0.05112	-4.53902	0.0005785	0.0017394	-0.27681
55897	MESP1	0.65156	2.39935	4.530222	0.0005878	0.0017639	-0.8444
79411	GLB1L	-0.5926	2.99787	-4.52898	0.0005891	0.0017663	-1.02673
5321	PLA2G4A	-0.8783	1.51577	-4.52637	0.0005919	0.0017724	-0.61413
128178	EDARADD	-1.4733	0.38407	-4.52245	0.0005961	0.0017826	-0.36185
253559	CADM2	-0.8654	3.43439	-4.60972	0.0005977	0.0017861	-1.1136
118672	PSTK	0.83937	2.07597	4.520496	0.0005982	0.0017872	-0.76662
100506495	LIFR-AS1	-1.1436	0.78498	-4.51627	0.0006028	0.0017988	-0.45954
51057	WDPCP	-0.6441	2.5509	-4.51306	0.0006063	0.0018072	-0.92605
79635	CCDC121	-0.968	0.99734	-4.5074	0.0006126	0.0018241	-0.52563
100506334	LINC00649	0.70281	4.0026	4.547936	0.000614	0.001827	-1.31801
23208	SYT11	-1.2918	1.1268	-4.5056	0.0006146	0.0018283	-0.54958
100506469	TMEM147-AS1	0.98856	1.28237	4.499721	0.0006212	0.0018462	-0.59911
10346	TRIM22	1.89991	0.23736	4.497948	0.0006232	0.0018504	-0.3626
132864	CPEB2	0.64433	3.83912	4.489022	0.0006333	0.0018775	-1.32962
2104	ESRRG	-1.0512	1.41233	-4.48786	0.0006347	0.0018806	-0.65503
136895	C7orf31	-0.6753	2.07302	-4.48503	0.0006379	0.0018885	-0.84185
168544	ZNF467	0.64631	2.6705	4.481163	0.0006424	0.0018992	-1.01402
84536	LINC01547	0.72994	1.91477	4.478428	0.0006456	0.0019082	-0.805
54852	PAQR5	0.63066	2.80178	4.463904	0.0006629	0.0019487	-1.08518
6041	RNASEL	-0.8802	1.6997	-4.45988	0.0006677	0.0019603	-0.78362
4778	NFE2	1.65314	0.08403	4.456507	0.0006718	0.0019696	-0.41797
554203	JPX	0.61992	2.63429	4.442082	0.0006897	0.0020159	-1.07674
303	ANXA2P1	-0.609	2.70125	-4.44114	0.0006909	0.002018	-1.10412
102723701	LOC102723701	1.38496	-0.07779	4.439276	0.0006932	0.0020216	-0.42979
5800	PTPRO	0.92293	1.60275	4.434919	0.0006987	0.0020363	-0.79917
2861	GPR37	-0.592	3.0446	-4.42702	0.0007089	0.0020587	-1.23054
55137	FIGN	0.73245	3.25498	4.424655	0.0007119	0.0020666	-1.28562
55080	TAPBPL	1.25094	-0.10689	4.424177	0.0007125	0.002068	-0.45512
53832	IL20RA	-1.8085	-0.51045	-4.42403	0.0007127	0.0020681	-0.38701
160428	ALDH1L2	-0.9564	1.79279	-4.42337	0.0007136	0.0020696	-0.8739
730051	ZNF814	0.6013	2.37943	4.41807	0.0007205	0.0020865	-1.04927
283212	KLHL35	0.84911	1.62608	4.410043	0.0007311	0.0021123	-0.85359
56140	PCDHA8	-1.3086	1.0235	-4.40814	0.0007337	0.0021181	-0.70557
401207	C5orf63	-1.2688	0.59959	-4.40278	0.0007409	0.002137	-0.62703
132989	C4orf36	1.0805	0.5598	4.402666	0.000741	0.002137	-0.61944

105376114	LOC105376114	1.11982	1.08483	4.394737	0.0007518	0.0021637	-0.74468
6320	CLEC11A	-0.9183	1.18337	-4.39401	0.0007528	0.0021661	-0.77796
729234	FAHD2CP	-0.6197	2.2232	-4.3748	0.0007797	0.0022298	-1.09073
79986	ZNF702P	-0.7015	2.93998	-4.37055	0.0007858	0.0022436	-1.30318
57538	ALPK3	1.18125	0.40844	4.370108	0.0007864	0.0022449	-0.64713
3012	H2AC8	0.82919	1.89378	4.369555	0.0007872	0.0022467	-0.99882
7782	SLC30A4	-0.8861	2.29344	-4.36322	0.0007964	0.0022678	-1.12435
594842	HAS2-AS1	-1.2478	0.32929	-4.36177	0.0007985	0.0022723	-0.65033
80758	PRR7	-0.6378	2.92165	-4.35157	0.0008135	0.002313	-1.33597
26212	OR2B6	1.75099	-0.43839	4.347272	0.00082	0.0023277	-0.53255
1050	CEBPA	-1.0623	1.17555	-4.34649	0.0008211	0.0023304	-0.86122
23127	COLGALT2	0.93798	1.16414	4.345893	0.000822	0.0023309	-0.85792
100287482	SMKR1	0.58793	2.46551	4.342554	0.0008271	0.0023425	-1.2159
23366	KIAA0895	-0.6308	2.33482	-4.33891	0.0008326	0.0023547	-1.18954
2566	GABRG2	0.89436	1.25901	4.321233	0.00086	0.002425	-0.92694
1040	CDS1	0.67435	1.98595	4.319251	0.0008631	0.0024328	-1.12246
51473	DCDC2	1.62618	-0.05744	4.319241	0.0008632	0.0024328	-0.6456
7700	ZNF141	0.91273	2.00986	4.318598	0.0008642	0.0024346	-1.12251
10793	ZNF273	0.62225	3.09202	4.31797	0.0008652	0.0024369	-1.44333
29126	CD274	0.68544	2.19217	4.312286	0.0008742	0.0024559	-1.19184
22849	CPEB3	0.85423	1.60922	4.301904	0.0008911	0.0024976	-1.05059
10810	WASF3	0.65824	3.00725	4.300697	0.000893	0.0025015	-1.45011
143903	LAYN	1.33233	0.28115	4.297459	0.0008984	0.0025142	-0.75343
284371	ZNF841	0.74159	4.03607	4.409079	0.0009162	0.0025573	-1.69546
133396	IL31RA	0.91671	1.23649	4.285593	0.0009181	0.0025605	-0.98772
401145	CCSER1	-1.2408	0.89544	-4.28327	0.0009221	0.0025692	-0.91002
648791	PPP1R3G	0.91137	0.82104	4.280278	0.0009272	0.0025811	-0.90359
200879	LIPH	0.88314	1.54174	4.266655	0.0009507	0.0026362	-1.09829
54984	PINX1	0.68225	2.40842	4.262837	0.0009574	0.0026519	-1.34585
154860	FEZF1-AS1	-0.6348	2.19812	-4.25524	0.0009708	0.0026857	-1.30738
3219	HOXB9	-1.2236	-0.09458	-4.25094	0.0009786	0.0027053	-0.77817
80833	APOL3	-1.415	0.23256	-4.23585	0.0010061	0.00277	-0.86007
5890	RAD51B	0.60334	2.50412	4.226555	0.0010235	0.0028118	-1.44459
100033416	SNORD116-4	-0.9736	0.74378	-4.2189	0.0010381	0.0028456	-1.00362
57594	HOMEZ	-0.6567	2.32435	-4.21568	0.0010443	0.0028607	-1.41754
8639	AOC3	1.1558	-0.14381	4.209261	0.0010567	0.0028873	-0.84374
3679	ITGA7	-0.6109	2.64657	-4.20888	0.0010574	0.0028888	-1.52546
340591	CA5BP1	0.60081	3.26516	4.204611	0.0010658	0.0029091	-1.70711
79957	PAQR6	1.03374	0.87009	4.195471	0.0010839	0.0029523	-1.06938
100505538	RBM26-AS1	0.99603	1.03732	4.192754	0.0010894	0.0029633	-1.11274
338557	FFAR4	-1.1949	0.73879	-4.19232	0.0010903	0.0029651	-1.04774
83872	HMCN1	-0.8071	1.88379	-4.18763	0.0010998	0.0029854	-1.34305
146664	MGAT5B	-0.6063	3.26032	-4.17555	0.0011246	0.003044	-1.76645
26468	LHX6	-1.0879	1.81836	-4.17222	0.0011315	0.003061	-1.34745
55876	GSDMB	0.67808	1.99348	4.167326	0.0011418	0.0030841	-1.41008
10239	AP3S2	1.42757	-0.28038	4.164349	0.0011481	0.0030985	-0.89778
8372	HYAL3	0.59498	3.16213	4.16129	0.0011546	0.0031141	-1.76019
100507347	VIM-AS1	0.73695	1.71516	4.15426	0.0011698	0.0031475	-1.35783
728927	ZNF736	-0.9392	1.68701	-4.15407	0.0011702	0.003148	-1.35083
5191	PEX7	-0.8072	2.5851	-4.14627	0.0011872	0.003189	-1.62022

57162	PELI1	-0.6377	2.61191	-4.14579	0.0011882	0.0031912	-1.63352
344148	NCKAP5	0.86024	1.34215	4.143961	0.0011923	0.0031997	-1.27963
102723390	FRG1EP	1.16199	0.45803	4.143069	0.0011943	0.0032033	-1.07679
126075	CCDC159	-0.6695	2.1409	-4.14087	0.0011991	0.0032157	-1.50603
92558	BICDL1	-0.7894	1.67003	-4.13659	0.0012087	0.0032351	-1.38271
79633	FAT4	0.68427	3.3517	4.135887	0.0012103	0.0032373	-1.86002
157	GRK3	-1.0284	0.42797	-4.12174	0.0012424	0.0033129	-1.11757
7556	ZNF10	0.84919	1.32374	4.108372	0.0012736	0.0033825	-1.34188
57549	IGSF9	-1.3281	0.3275	-4.08806	0.0013226	0.0034921	-1.15411
389792	IER5L	-0.8012	1.51196	-4.08291	0.0013353	0.0035198	-1.44293
137994	LETM2	0.70546	1.73386	4.081556	0.0013386	0.0035272	-1.50078
401027	C2orf66	-1.1821	-0.29653	-4.07998	0.0013426	0.0035346	-1.05871
6876	TAGLN	-0.9188	1.18649	-4.07679	0.0013505	0.0035527	-1.37145
5166	PDK4	-0.6984	1.67096	-4.06823	0.0013722	0.003603	-1.51384
4828	NMB	-0.8006	1.86858	-4.0607	0.0013916	0.0036456	-1.5789
8348	H2BC17	1.07647	0.40041	4.053095	0.0014114	0.0036929	-1.23489
83700	JAM3	-1.2721	0.36705	-4.04449	0.0014342	0.0037418	-1.24389
219539	YPEL4	-1.2812	0.4075	-4.04397	0.0014356	0.0037447	-1.25315
7425	VGF	0.84151	1.57148	4.039226	0.0014483	0.0037771	-1.53468
158219	TTC39B	-1.001	0.98608	-4.0319	0.0014682	0.0038251	-1.40612
101928378	PTOV1-AS2	0.93207	1.29214	4.024533	0.0014885	0.0038716	-1.4902
3690	ITGB3	-0.6198	2.82781	-4.01415	0.0015176	0.003936	-1.94676
101927279	EP300-AS1	-1.1711	0.08169	-4.01347	0.0015195	0.0039402	-1.24881
730268	ANAPC1P4	1.72326	-0.05711	4.012382	0.0015226	0.0039458	-1.2069
50489	CD207	-1.1615	-0.12298	-4.00148	0.0015539	0.004013	-1.23449
10319	LAMC3	-0.6194	2.27029	-3.99867	0.0015621	0.0040308	-1.81383
389084	SNORC	-0.8126	1.93083	-3.99807	0.0015639	0.0040345	-1.71502
100506713	ALOX12-AS1	0.90057	0.75027	3.996331	0.0015689	0.0040445	-1.41865
2100	ESR2	1.25586	0.66655	3.981876	0.0016119	0.0041406	-1.41871
314	AOC2	0.79549	1.11093	3.975208	0.0016321	0.0041866	-1.54275
55765	INAVA	-0.7425	1.64513	-3.97303	0.0016387	0.0042011	-1.6861
9824	ARHGAP11A	-0.6368	1.99894	-3.97284	0.0016393	0.0042018	-1.78493
6457	SH3GL3	0.92183	1.87617	3.970402	0.0016468	0.0042167	-1.74224
1513	CTSK	0.77822	1.37965	3.968297	0.0016533	0.0042325	-1.62166
374462	PTPRQ	2.80358	-0.60516	4.084322	0.0016674	0.004266	-1.1191
57337	SENP7	-0.6362	3.84889	-3.98229	0.0017052	0.0043495	-2.33803
79823	CAMKMT	-0.7863	1.82308	-3.94549	0.0017253	0.0043974	-1.78559
140686	WFDC3	-0.9074	1.72017	-3.94016	0.0017426	0.0044344	-1.76431
4645	MYO5B	0.64504	1.80917	3.935975	0.0017563	0.0044621	-1.79823
64901	RANBP17	-0.6151	2.22382	-3.93294	0.0017663	0.004484	-1.92562
401588	ZNF674-AS1	0.72671	1.73327	3.921437	0.0018048	0.004567	-1.80336
23600	AMACR	-0.663	1.86313	-3.91302	0.0018335	0.0046267	-1.86009
3600	IL15	0.89135	1.06136	3.906671	0.0018555	0.004671	-1.65794
642366	LOC642366	-1.432	-0.01373	-3.90228	0.0018708	0.0047068	-1.43272
84059	ADGRV1	-0.7309	4.62919	-4.07383	0.0018761	0.0047193	-2.54539
79025	FNDC11	-1.063	0.2984	-3.89317	0.001903	0.0047756	-1.51765
9022	CLIC3	-1.0701	0.29071	-3.89217	0.0019066	0.0047827	-1.51812
2625	GATA3	-1.4035	0.04185	-3.88482	0.0019331	0.0048415	-1.47576
3931	LCAT	0.7315	2.08618	3.88189	0.0019437	0.0048605	-1.97544
79805	VASH2	-0.585	2.44888	-3.86998	0.0019877	0.0049539	-2.11162

9754	STARD8	0.82926	2.22862	3.863656	0.0020114	0.0050101	-2.0461
124220	ZG16B	-0.6922	2.6623	-3.86214	0.0020172	0.0050234	-2.18637
80063	ATF7IP2	-0.5929	2.20066	-3.86181	0.0020184	0.0050255	-2.05469
7318	UBA7	1.04603	0.64583	3.85895	0.0020293	0.0050476	-1.65082
60686	C14orf93	-0.6144	2.35426	-3.85534	0.0020431	0.005079	-2.11145
144110	TMEM86A	-0.7648	1.5802	-3.84887	0.0020681	0.005132	-1.90373
201625	DNAH12	-0.9763	0.7283	-3.84847	0.0020697	0.0051349	-1.69363
9985	REC8	1.16938	-0.00874	3.844709	0.0020843	0.0051655	-1.54255
5651	TMPRSS15	-1.221	-0.13209	-3.83403	0.0021266	0.0052567	-1.5427
100652730	LINC00659	-0.7223	1.74209	-3.83228	0.0021336	0.0052731	-1.97984
269	AMHR2	1.14771	0.38414	3.826064	0.0021587	0.0053299	-1.65508
344787	ZNF860	0.978	0.64669	3.819758	0.0021845	0.005383	-1.72613
647024	C6orf132	1.12458	0.25141	3.819095	0.0021872	0.0053877	-1.64201
57047	PLSCR2	0.96036	0.37323	3.814487	0.0022063	0.0054304	-1.67852
652	BMP4	-0.7082	2.09948	-3.80875	0.0022302	0.0054851	-2.12381
101928607	TRPM2-AS	0.77926	1.70563	3.80405	0.0022501	0.0055275	-2.018
100379224	LOC100379224	0.9295	0.93403	3.801322	0.0022616	0.0055516	-1.82656
79661	NEIL1	0.76529	1.16719	3.793552	0.002295	0.0056259	-1.90004
729440	CCDC61	-0.8018	1.31147	-3.78841	0.0023174	0.005673	-1.9492
84812	PLCD4	0.66031	2.54062	3.785026	0.0023322	0.0057027	-2.29217
4565	TRNI	-0.7944	1.35205	-3.77819	0.0023624	0.0057689	-1.97862
163071	ZNF114	-1.2758	-0.27329	-3.76372	0.0024278	0.0059114	-1.64746
346007	EYS	0.88024	1.5093	3.762486	0.0024334	0.0059229	-2.04251
729603	CHP1P2	1.03799	0.28602	3.760714	0.0024416	0.0059393	-1.76015
57576	KIF17	0.71402	1.41903	3.757002	0.0024587	0.0059742	-2.03376
147699	PPM1N	-1.1152	0.18862	-3.75043	0.0024894	0.0060407	-1.76189
9856	KIAA0319	0.98099	0.91233	3.747073	0.0025052	0.0060709	-1.92217
100302640	LINC00882	1.03918	0.45853	3.746514	0.0025079	0.006075	-1.82187
340037	PRR7-AS1	-1.1024	0.53688	-3.74052	0.0025364	0.0061278	-1.8532
23066	CAND2	-0.6059	2.26797	-3.73571	0.0025595	0.0061766	-2.31469
222234	FAM185A	0.67368	2.15925	3.732231	0.0025764	0.0062126	-2.28299
259217	HSPA12A	-0.645	1.95929	-3.72754	0.0025993	0.0062632	-2.24037
103689912	TBX2-AS1	1.44396	0.05002	3.726508	0.0026044	0.0062742	-1.76793
353088	ZNF429	0.97853	0.62704	3.723548	0.002619	0.0063022	-1.90268
100125288	ZGLP1	1.25563	0.21994	3.72009	0.0026362	0.0063363	-1.81745
100192378	ZFHX4-AS1	-0.7853	1.33957	-3.71708	0.0026512	0.0063652	-2.09202
9737	GPRASP1	1.02401	0.5509	3.705565	0.0027096	0.0064875	-1.91976
3689	ITGB2	1.32209	-0.01424	3.695966	0.0027592	0.0065958	-1.81577
64284	RAB17	1.1434	0.24302	3.685118	0.0028164	0.0067135	-1.89065
3215	HOXB5	0.77851	1.25592	3.682297	0.0028315	0.0067444	-2.13311
9424	KCNK6	-0.6378	1.72731	-3.67865	0.0028511	0.0067835	-2.26959
151295	SLC23A3	1.0953	-0.06615	3.673427	0.0028794	0.0068406	-1.85358
124093	CCDC78	1.01579	0.24916	3.67191	0.0028877	0.0068526	-1.91994
101927797	MIR548XHG	1.14838	-0.18211	3.670332	0.0028963	0.0068709	-1.83728
493900	TMEM9B-AS1	-0.755	1.22252	-3.66784	0.00291	0.0068966	-2.15656
5414	SEPTIN4	-1.1467	-0.3276	-3.66672	0.0029162	0.0069086	-1.82133
374819	LRRC37A3	0.73812	1.79203	3.656026	0.0029758	0.0070303	-2.32442
10309	CCNO	0.86832	0.48249	3.634799	0.0030979	0.0072806	-2.04174
26047	CNTNAP2	-1.0136	0.39294	-3.63078	0.0031216	0.0073297	-2.03143
57801	HES4	-0.8092	0.81322	-3.62857	0.0031347	0.0073564	-2.13205

103695436	LINC01269	1.28258	0.09454	3.617586	0.0032007	0.0075057	-1.98549
6286	S100P	0.78538	1.29593	3.61117	0.0032399	0.0075863	-2.27798
7480	WNT10B	0.93515	1.14681	3.611025	0.0032407	0.007587	-2.23686
79930	DOK3	0.67689	1.97803	3.602598	0.003293	0.0076979	-2.47921
163087	ZNF383	0.60293	2.06519	3.598366	0.0033195	0.0077477	-2.51415
399726	MIR1915HG	-1.0183	0.9339	-3.59833	0.0033197	0.0077477	-2.21305
100289341	MAN1B1-DT	0.79262	0.94653	3.594391	0.0033446	0.0078016	-2.22457
339451	KLHL17	-0.6283	2.86317	-3.59425	0.0033455	0.0078022	-2.76067
8209	GATD3A	-0.9478	0.904	-3.59009	0.003372	0.0078553	-2.22375
91409	CCDC74B	0.63619	1.40926	3.58785	0.0033864	0.0078816	-2.35452
340277	FAM221A	0.70068	1.44028	3.586631	0.0033943	0.007894	-2.36347
388327	C17orf100	-0.8614	1.0026	-3.58359	0.0034139	0.007934	-2.26077
284467	TAFA3	1.11185	-0.15035	3.577031	0.0034567	0.0080231	-2.01834
56063	TMEM234	-0.6432	2.07422	-3.56774	0.0035182	0.008148	-2.57875
283518	KCNRG	1.01468	0.11447	3.566056	0.0035295	0.0081726	-2.0921
374666	WASH3P	1.01337	0.79403	3.565101	0.0035359	0.0081849	-2.23935
360	AQP3	1.09233	0.14428	3.560384	0.0035677	0.0082446	-2.10627
112817	HOGA1	-1.277	-0.26681	-3.54757	0.0036556	0.0084217	-2.05163
255809	C19orf38	1.28471	-0.39962	3.536703	0.0037319	0.0085819	-2.04426
10814	CPLX2	-1.2917	0.2167	-3.52821	0.0037926	0.0087011	-2.18207
84807	NFKBID	0.83625	1.12725	3.523998	0.0038231	0.008762	-2.40065
389906	LOC389906	0.64357	1.86599	3.513291	0.0039018	0.0089212	-2.61929
64137	ABCG4	-0.7888	0.8792	-3.51117	0.0039176	0.0089522	-2.37001
10071	MUC12	-1.7741	0.53478	-3.60057	0.0039183	0.0089522	-2.21005
728609	SDHAP3	-0.7376	1.50091	-3.50885	0.0039349	0.0089771	-2.53038
51131	PHF11	0.65721	2.37996	3.508571	0.003937	0.0089787	-2.77445
100874048	DGUOK-AS1	0.65822	1.80984	3.50817	0.00394	0.0089839	-2.61265
4636	MYL5	0.7676	1.58738	3.502859	0.00398	0.0090573	-2.55933
84125	LRRIQ1	1.03737	0.44076	3.495097	0.0040392	0.0091854	-2.29241
2353	FOS	0.85928	1.17539	3.48476	0.0041194	0.0093411	-2.48667
4824	NKX3-1	-0.6017	1.6935	-3.48169	0.0041435	0.0093808	-2.63659
144811	LACC1	0.84475	1.49924	3.472436	0.0042172	0.0095187	-2.59252
2706	GJB2	-0.6931	1.71627	-3.47096	0.0042291	0.0095422	-2.66201
143662	MUC15	-0.6798	1.81307	-3.46539	0.0042742	0.0096284	-2.69966
30008	EFEMP2	-0.6473	1.85838	-3.46165	0.0043047	0.0096801	-2.71991
10736	SIX2	0.72728	2.54249	3.456453	0.0043475	0.0097677	-2.91873
387841	RPL13AP20	1.067	0.26646	3.455084	0.0043589	0.009788	-2.33025
100130238	LOC100130238	-0.9103	0.0849	-3.45495	0.00436	0.0097889	-2.30035
10060	ABCC9	0.90972	0.39428	3.452733	0.0043784	0.0098198	-2.36558
104355135	LINC00431	0.98991	0.75467	3.450832	0.0043943	0.0098502	-2.44718
122970	ACOT4	-0.9568	0.04956	-3.44983	0.0044027	0.0098656	-2.3021
119016	AGAP4	-0.9832	0.06752	-3.43955	0.0044898	0.0100324	-2.32434
100128398	LOC100128398	-0.9864	0.00076	-3.43215	0.0045536	0.0101588	-2.3247
2027	ENO3	1.04511	0.58768	3.42429	0.0046223	0.0103012	-2.45749
353324	SPATA12	1.07308	0.04814	3.41953	0.0046644	0.0103878	-2.35213
102724378	LOC102724378	-0.7551	0.77894	-3.41392	0.0047145	0.0104902	-2.53115
56605	ERO1B	-0.5973	1.86682	-3.41155	0.0047359	0.0105286	-2.81882
100131551	LINC00887	-0.7889	0.49538	-3.40948	0.0047546	0.0105609	-2.47462
2069	EREG	0.83353	0.44285	3.397105	0.0048681	0.0107832	-2.48215
2151	F2RL2	-1.2952	-0.23359	-3.39706	0.0048685	0.0107832	-2.33924

348926	FAM86EP	0.66104	1.66548	3.382298	0.0050076	0.0110513	-2.81341
101060264	FOXP4-AS1	1.05784	-0.23544	3.375184	0.005076	0.0111809	-2.38064
80765	STARD5	0.89089	1.25059	3.370822	0.0051184	0.0112607	-2.72083
55056	GABPB1-IT1	-0.7082	1.52907	-3.36912	0.0051351	0.0112895	-2.80468
23180	RFTN1	0.68589	1.80697	3.35051	0.0053207	0.0116492	-2.91196
55287	TMEM40	0.7252	0.92638	3.336692	0.0054629	0.0119276	-2.70922
101927532	LINC01736	1.00529	0.51284	3.320489	0.0056345	0.0122497	-2.63803
54894	RNF43	-1.0319	-0.20441	-3.31654	0.0056772	0.0123277	-2.50073
441733	PRKXP1	0.93044	-0.00643	3.316538	0.0056772	0.0123277	-2.53757
2073	ERCC5	0.76857	0.51296	3.304208	0.0058124	0.0125696	-2.67421
8796	SCEL	1.63681	-0.6133	3.299867	0.0058608	0.0126616	-2.43936
101927612	RNF139-AS1	-0.9563	0.0342	-3.29331	0.0059347	0.0127994	-2.59283
11141	IL1RAPL1	-1.016	0.16536	-3.29068	0.0059646	0.0128435	-2.62292
10324	KLHL41	0.8439	0.31105	3.280484	0.0060818	0.0130635	-2.67322
57835	SLC4A5	0.77495	0.97105	3.278712	0.0061025	0.0131034	-2.82912
79873	NUDT18	-0.5942	1.66283	-3.27855	0.0061044	0.0131053	-3.01549
400566	C17orf97	0.61664	1.37719	3.272848	0.0061712	0.0132353	-2.94564
5383	PMS2P5	1.00356	-0.09963	3.268545	0.0062222	0.0133333	-2.60738
56114	PCDHGA1	-0.7627	0.54845	-3.25792	0.0063497	0.013577	-2.7728
728024	LOC728024	0.74314	0.78815	3.256838	0.0063629	0.0136028	-2.82706
3075	CFH	0.92316	-0.31753	3.255689	0.0063769	0.0136304	-2.59085
11170	FAM107A	-0.7418	1.4501	-3.25398	0.0063978	0.0136705	-3.00142
84071	ARMC2	-0.5803	1.52791	-3.23437	0.006642	0.0141257	-3.06266
340075	ARSI	-0.6483	1.46833	-3.22986	0.0066995	0.0142315	-3.0544
255374	MBLAC1	-0.984	0.04486	-3.2271	0.0067349	0.0142947	-2.71839
353091	RAET1G	0.69824	1.26523	3.226616	0.0067412	0.0143056	-3.00214
3750	KCND1	-0.592	1.86061	-3.22163	0.0068057	0.0144304	-3.17874
100506639	LOC100506639	-1.021	1.46352	-3.2256	0.0068556	0.0145144	-3.06062
26815	SNORD36A	1.07541	-0.13564	3.203537	0.0070452	0.0148713	-2.71993
100379571	BACE1-AS	-0.9555	0.50065	-3.20168	0.0070703	0.0149168	-2.86392
2892	GRIA3	0.62824	1.27166	3.200302	0.0070889	0.0149455	-3.05533
283229	CRACR2B	0.62551	1.76242	3.186566	0.0072775	0.0153132	-3.21325
170082	TCEANC	0.61029	1.35415	3.185588	0.0072911	0.0153368	-3.10493
10002	NR2E3	1.15028	0.63621	3.182688	0.0073317	0.0154017	-2.92083
257396	LOC257396	-0.6236	1.88484	-3.17491	0.0074415	0.015604	-3.27408
158572	USP27X-AS1	0.60939	1.45944	3.171283	0.0074932	0.0156978	-3.16016
254013	ETFBKMT	0.84449	0.91605	3.164331	0.0075935	0.0158776	-3.02933
3751	KCND2	0.72209	1.20217	3.148244	0.0078307	0.0163151	-3.13401
647946	MIR924HG	-0.7854	1.08322	-3.1434	0.0079035	0.0164534	-3.11619
23562	CLDN14	0.78216	0.78394	3.1391	0.0079687	0.0165674	-3.04694
200316	APOBEC3F	-0.5932	1.53019	-3.13542	0.0080251	0.0166736	-3.25069
84206	MEX3B	0.95219	0.6305	3.129886	0.0081104	0.0168315	-3.02493
5630	PRPH	0.83218	0.2529	3.126435	0.008164	0.0169236	-2.9498
100505636	MALINC1	-0.7465	1.06734	-3.11958	0.0082717	0.0171217	-3.15731
144406	CFAP251	0.90612	-0.03426	3.115424	0.0083377	0.0172358	-2.90851
29114	TAGLN3	-0.7664	0.61573	-3.11458	0.0083512	0.017258	-3.05752
51276	ZNF571	0.70681	0.98208	3.104844	0.0085081	0.0175508	-3.1614
342909	ZNF284	-0.7023	0.84674	-3.09665	0.0086423	0.017773	-3.14696
3892	KRT86	0.66934	2.6085	3.09518	0.0087138	0.0179112	-3.634
27152	INTU	0.58007	1.91629	3.077085	0.0089717	0.018376	-3.46547

10170	DHRS9	-0.7085	1.06035	-3.07608	0.008989	0.0184055	-3.23838
103344932	PGM5P4-AS1	-0.7275	0.59513	-3.07226	0.0090549	0.0185253	-3.13305
18	ABAT	-1.0763	-0.34954	-3.06853	0.0091197	0.0186399	-2.93279
150946	GAREM2	0.67709	1.66502	3.060227	0.0092655	0.0189046	-3.42358
4610	MYCL	-0.6158	1.13941	-3.05797	0.0093056	0.0189741	-3.29388
101927245	LOC101927245	1.0509	-0.16962	3.056224	0.0093367	0.0190162	-2.98852
3908	LAMA2	0.63752	1.15275	3.055671	0.0093466	0.0190302	-3.29755
285613	RELL2	-0.8128	0.86016	-3.04736	0.0094962	0.0192854	-3.24074
728833	FAM72D	-0.6872	1.64034	-3.04143	0.0096044	0.0194647	-3.45663
643072	LOC643072	0.77354	0.79811	3.034351	0.0097353	0.0196828	-3.24726
253190	SERHL2	-0.7856	0.27426	-3.03237	0.0097722	0.019745	-3.13435
4515	MTCP1	0.76884	0.70012	3.026664	0.0098793	0.0199376	-3.23871
9348	NDST3	0.80308	0.34719	3.023958	0.0099305	0.0200234	-3.16227
55615	PRR5	0.8408	1.04814	3.016506	0.0100729	0.0202687	-3.34017
684	BST2	0.91098	0.51345	3.012571	0.0101489	0.0204023	-3.21807
25802	LMOD1	-1.076	-0.29442	-3.00834	0.0102313	0.0205451	-3.05594
119385	AGAP11	0.62679	1.41494	2.98814	0.0106338	0.0212643	-3.49293
100996763	NOTCH2NLB	0.90516	0.03619	2.983171	0.0107352	0.0214418	-3.16888
833	CARS1	0.72845	1.40938	2.976326	0.0108765	0.0216728	-3.51078
387849	REP15	0.75841	0.61693	2.976155	0.01088	0.0216765	-3.31337
84190	METTL25	0.65519	1.26319	2.973252	0.0109405	0.0217765	-3.48047
83401	ELOVL3	-0.8039	0.42557	-2.97249	0.0109565	0.0218014	-3.27888
347744	C6orf52	0.7571	0.78326	2.969583	0.0110175	0.0218919	-3.36491
7273	TTN	-0.6983	4.91144	-3.15415	0.0110212	0.0218959	-4.35467
84989	JMJD1C-AS1	-0.6105	2.02445	-2.96772	0.0110568	0.0219494	-3.70594
79819	DNAI4	-0.8349	-0.06157	-2.96435	0.0111281	0.0220773	-3.1881
643314	KIAA0754	-0.6248	5.10593	-3.11077	0.0112139	0.0222157	-4.43911
402778	IFITM10	0.65389	1.02691	2.960174	0.0112172	0.0222157	-3.44468
692086	SNORD17	-0.8504	0.47477	-2.95653	0.0112955	0.0223395	-3.31892
283970	PDXDC2P-NPIPE	0.63553	1.67045	2.953075	0.0113702	0.0224664	-3.6285
7775	ZNF232	-0.6188	1.975	-2.95035	0.0114296	0.0225696	-3.72472
150590	C2orf15	0.6346	0.8488	2.949739	0.0114429	0.0225888	-3.42055
2313	FLI1	-0.6646	1.1192	-2.9385	0.0116911	0.0230215	-3.5116
9957	HS3ST1	-0.8778	0.05759	-2.93159	0.0118463	0.023291	-3.27323
3955	LFNG	1.07201	-0.13009	2.927549	0.0119379	0.023453	-3.23357
257313	UTS2B	-1.0619	-0.3156	-2.92609	0.0119713	0.0235041	-3.20272
5639	PRRG2	0.7755	0.1028	2.922152	0.0120615	0.0236556	-3.29869
349667	RTN4RL2	-0.6913	0.92786	-2.91685	0.0121841	0.0238591	-3.50391
26059	ERC2	-0.8536	0.22479	-2.90173	0.0125407	0.0244669	-3.3646
79081	LBHD1	0.70892	0.98148	2.895876	0.0126815	0.0247189	-3.55238
6029	RN7SL1	-0.5934	2.11526	-2.88298	0.0129972	0.02528	-3.89248
100506286	TBC1D8-AS1	-0.6322	0.81972	-2.8437	0.0140074	0.0269559	-3.61366
92737	DNER	-0.7815	0.65074	-2.84112	0.0140765	0.0270642	-3.57513
2086	ERV3-1	0.59348	1.85018	2.830435	0.0143658	0.0275662	-3.90948
100506881	MKLN1-AS	0.96616	0.59313	2.822377	0.0145879	0.0279333	-3.5887
729178	STXBP5-AS1	1.11938	-0.1321	2.81174	0.0148862	0.0283845	-3.44475
57639	CCDC146	-0.7583	0.44186	-2.80496	0.0150796	0.0287019	-3.5937
55640	FLVCR2	0.65781	0.98623	2.803634	0.0151176	0.0287699	-3.72557
257169	C9orf43	0.86023	0.11653	2.789008	0.0155441	0.0294536	-3.54542
285175	UNC80	-0.6526	0.94388	-2.78265	0.0157331	0.0297452	-3.75679

101928673	PRDX6-AS1	0.62892	1.30888	2.774354	0.0159833	0.0301642	-3.86289
1308	COL17A1	0.66228	0.68017	2.774138	0.0159898	0.0301722	-3.70463
100616668	TPTE2P5	-0.5902	1.02539	-2.75275	0.0166529	0.0312331	-3.83365
1608	DGKG	-0.6769	2.03324	-2.75207	0.0166747	0.0312648	-4.10921
25999	CLIP3	0.82523	0.28198	2.736749	0.0171668	0.032055	-3.67811
6948	TCN2	0.59478	1.27619	2.727766	0.017462	0.0325535	-3.94079
3953	LEPR	-0.6686	0.57874	-2.70173	0.0183459	0.0339275	-3.81607
7051	TGM1	0.87061	-0.00999	2.69989	0.0184101	0.0340263	-3.68041
728621	CCDC30	-0.7358	0.66794	-2.69704	0.0185099	0.034186	-3.84463
100130733	LRRC70	-0.9308	-0.04231	-2.67952	0.0191347	0.035166	-3.7128
9628	RGS6	-0.7125	0.0332	-2.67608	0.0192597	0.0353754	-3.7384
400932	LINC00898	-0.8275	0.54263	-2.67546	0.0192823	0.0354117	-3.85255
100133234	SBF1P1	0.67493	0.39021	2.672496	0.0193907	0.0355852	-3.82213
222643	UNC5CL	0.69595	0.7467	2.66805	0.0195546	0.0358445	-3.91444
101928578	RAP2C-AS1	0.63237	1.0193	2.664729	0.019678	0.0360602	-3.98932
2921	CXCL3	0.78692	0.01984	2.664323	0.0196931	0.0360827	-3.75253
339456	TMEM52	-0.5884	1.56816	-2.6556	0.0200207	0.0365985	-4.15629
146429	SLC22A31	-0.6985	0.3481	-2.65341	0.0201038	0.0367293	-3.8498
442582	STAG3L2	0.67601	1.62847	2.640469	0.020602	0.0375585	-4.19498
100507250	LOC100507250	0.85978	0.01844	2.63555	0.0207945	0.0378442	-3.80258
100506606	LOC100506606	0.82142	0.12706	2.631176	0.0209671	0.0380875	-3.83498
101060200	ZNF891	0.88503	0.0579	2.628831	0.0210603	0.0382074	-3.82305
84222	TMEM191A	-0.8414	0.35522	-2.62705	0.0211313	0.0383145	-3.89658
116039	OSR2	-0.5923	0.97114	-2.61697	0.0215374	0.0389613	-4.06802
2306	FOXD2	-0.9591	0.12457	-2.61185	0.0217465	0.0392841	-3.87081
25806	VAX2	-0.899	-0.24963	-2.60936	0.0218493	0.0394417	-3.79551
80763	SPX	0.80953	0.00071	2.604655	0.022044	0.0397594	-3.85556
729020	RPEL1	-0.6239	0.77937	-2.60215	0.0221483	0.0399247	-4.04629
4092	SMAD7	-0.6426	0.45197	-2.59625	0.0223965	0.0402922	-3.97809
201294	UNC13D	0.68129	0.29408	2.574819	0.0233198	0.0417698	-3.97675
728039	SSR4P1	0.77684	0.43659	2.571712	0.0234567	0.0419677	-4.01317
100144602	EPHA5-AS1	-0.7318	0.9274	-2.57157	0.0234628	0.0419727	-4.13727
100750247	HIF1A-AS2	0.70353	0.31742	2.568949	0.0235791	0.042151	-3.99237
100996702	LINC01356	-0.7385	0.35769	-2.56709	0.0236618	0.0422751	-4.0076
3233	HOXD4	0.61669	0.62862	2.562526	0.023866	0.042592	-4.07811
202020	TAPT1-AS1	-0.7451	0.32985	-2.55777	0.0240808	0.0428971	-4.01765
8605	PLA2G4C	0.63155	0.61526	2.557644	0.0240864	0.042901	-4.0832
28984	RGCC	0.76278	0.49053	2.541775	0.0248164	0.0439669	-4.08023
100506990	LOC100506990	0.74278	0.1796	2.537308	0.0250257	0.0442933	-4.01689
79917	MAGIX	0.70197	0.32822	2.514198	0.0261362	0.0460356	-4.09269
101243545	LINC02067	0.73852	0.06372	2.513632	0.026164	0.0460718	-4.03299
57615	ZNF492	0.78176	0.43105	2.510091	0.0263385	0.0463154	-4.12215
201191	SAMD14	0.6095	0.75626	2.503801	0.0266512	0.046787	-4.21449
154791	FMC1	0.58723	0.58401	2.502892	0.0266967	0.0468345	-4.1749
158401	SHOC1	0.74208	0.376	2.498655	0.0269098	0.0471366	-4.13093
51750	RTEL1	-0.7498	0.52219	-2.49471	0.0271095	0.0474341	-4.17545
2650	GCNT1	0.87999	0.17547	2.49103	0.0272973	0.047743	-4.09598
9235	IL32	0.69409	0.67524	2.47379	0.0281933	0.049067	-4.24633
